# Supplementary material for: Multisystemic resilience to shocks: a temporal analysis of health, fundamental rights and freedoms, and economic resilience during the first wave of the COVID-19 pandemic in 22 European countries
Source: BMJ Open. 2023 Jul 7;13(7):e065445. doi: 10.1136/bmjopen-2022-065445 (PMC10335565; doi:10.1136/bmjopen-2022-065445)
Supplement: Supplementary data [file bmjopen-2022-065445supp001.pdf]

15.06.2023

# Multisystemic resilience to shocks: a temporal analysis of health, fundamental rights and freedoms, and economic resilience during the first wave of COVID-19's in 22 European countries

Mia Clausin, Alicia Rieckhoff, Fabrizio Tediosi, Chantal Morel, Yuliya Kaspiarovich, Nicolas Levrat, Didier Wernli

## Supplementary material

**Supplementary material 1 – Method used to derive metrics about multisystemic resilience**

We calculated several Z-scores to compare the overall balance in terms of resilience in different systems. Regarding excess mortality (1), the Z-score for 2020 was calculated as the value for 2020 (x) minus the mean for the period 2015 – 2020 (μ). The difference was then divided by the standard deviation (σ). Regarding stringency (2), we calculated a Z-score for the 2020 period to provide information about the stringency of a country's response compared to other countries. Finally, a Z-score was calculated for the Liberal Democracy Index (3) from V-Dem (https://v-dem.net/) from 2015 to 2020 to evaluate how liberal countries were before the pandemic.

Two different multisystemic resilience metrics were derived from these three Z-scores. To avoid computing metrics with negative Z-score values, the lowest value of each Z-score was set to 0 and all scores were modified accordingly. For example, regarding excess mortality, the lowest excess mortality of -1.350 for Iceland was set to zero. For the liberal democracy score, we also inverted the sign of each value meaning that a low z-score corresponds to a more liberal democracy than a high score and then put the lowest value to zero (Denmark and Sweden). The numbers are reported in supplementary material 4.

The first metric is the *difference between z-scores for stringency of the response and for excess mortality* which gives an indication of the adequation between the response and the problem based on a comparison between countries during the first wave of the COVID-19 pandemic in Europe. A negative score suggests that overall, the response may have been insufficient compared to the magnitude of the problem while a highly positive score suggests that the response may have been overtly stringent to the magnitude of the problem experienced by the country. It relies on the assumption that a higher stringency index is politically more justifiable in case of higher excess mortality (it must however be noted that the precautionary principle may impose to take some measures to protect public health early on).

The second metric is the *difference between z-scores for the state of (il)liberal democracy and for the stringency of the response* which gives an idea about the departure from the traditional way of governing during the first wave of the COVID-19 pandemic in Europe in early 2020. This score assumes that a liberal country may be more likely to protect fundamental rights and freedoms even in times of emergency. A negative score suggests that a country that is traditionally liberal was more stringent than one would have expected to be compared to other European countries during the first wave of the COVID-19. Supplementary material 5 shows a plot of the two metrics against each other.



Clausin M, *et al. BMJ Open* 2023; 13:e065445. doi: 10.1136/bmjopen-2022-065445

15.06.2023

**Supplementary material 2 – Box plots and whisker for robustness in health systems in 22 countries based on excess mortality during week 3 to 35 for the years 2015 – 2020.**

We used the 1.5 interquartile range method to detect outliers (lower bound: (Q1 - 1.5 * IQR) and upper bound: (Q3 + 1.5 * IQR). The outlier value of excess mortality observed for Denmark and Germany was not in 2020. This method for the detection of outliers has an important limitation as it depends on the variability of excess mortality in the previous years. For example, the Netherlands had an excess mortality of 10,05% in 2020 but this value was not an outlier according to the 1.5 IQR rule due to variability of excess mortality during the 2015 – 2020 period.

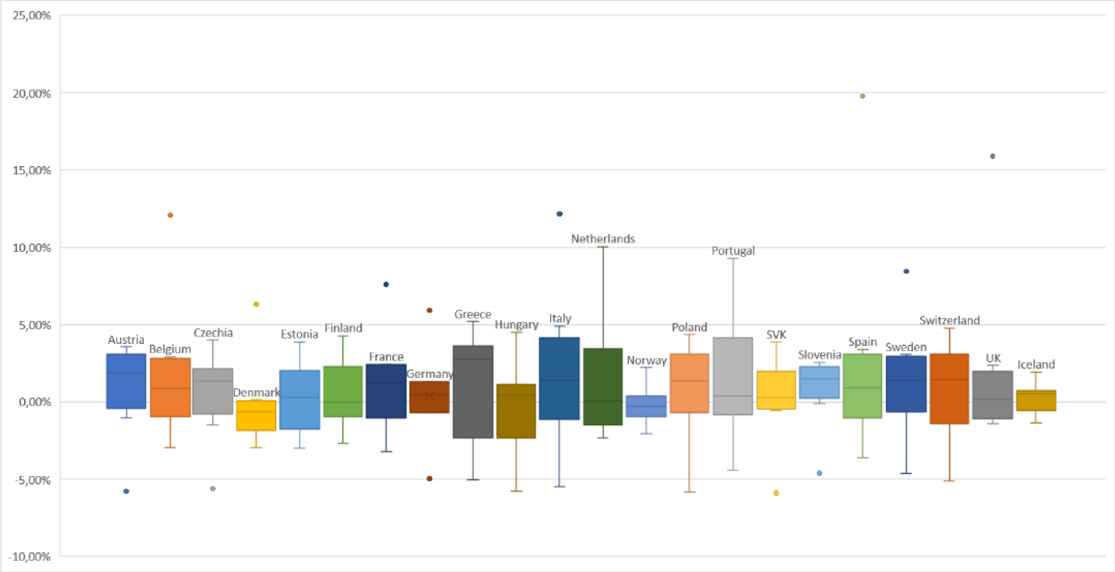


**Supplementary material 3 - Health system resilience, resistance, recovery and robustness**

| Countries | Resilience (cumulative excess mortality) | Resistance rate | Recovery rate | Robustness |
|---|---|---|---|---|
| Austria | 109.00% | 3.16% (>0.05) | -4.86% (<0.01) | Yes |
| Belgium | 398.35% | 29.89% (<0.01) | -14.67% (<0.01) | No |
| Czechia | 45.39% | 2.84% (<0.05) | -2.23% (>0.05) | Yes |
| Germany | 43.68% | 4,96% (<0.05) | -1.73% (<0.01) | Yes |
| Denmark | -3.49% | 5.33% (n/a) | -9.18% (n/a) | Yes |
| Spain | 653.18% | 45.28% (<0.05) | -26.23% (<0.001) | No |
| Estonia | 24.27% | 4,94% (>0.05) | -5,37 (>0.05) | Yes |
| Finland | 99.68% | 2,5% (>0.05) | -9.34% (n/a) | Yes |
| France | 251.33% | 17.29% (<0.01) | -8.30% (<0.01) | No |
| Greece | 119.68% | 7.99% (>0.05) | -8.22% (>0.05) | Yes |
| Hungary | -100.28% | 10.23% (n/a) | -6.22 (n/a) | Yes |
| Iceland | -43.83% | 9.45% (>0.05) | 44.63% (n/a) | Yes |
| Italy | 401.12% | 23.68% (<0.01) | -10.77% (<0.001) | No |
| Netherlands | 331.56% | 20.52% (<0.01) | -10.73% (<0.001) | Yes |
| Norway | -37.99% | 10,30% (n/a) | -5,37% (n/a) | Yes |
| Poland | 103.42% | 2.47% (>0.05) | -11.68% (n/a) | Yes |
| Portugal | 307.00% | 5.11% (<0,01) | -3.35% (<0.01) | Yes |
| Slovakia | -7.74% | 5.64% (>0.05) | -9.68% (n/a) | Yes |
| Slovenia | 84.56% | 19.55% (n/a) | -5.69% (<0.01) | Yes |
| Sweden | 278.98% | 13.80% (<0.05) | -3.74% (<0.001) | No |
| Switzerland | 108.57% | 14.50% (<0.01) | -9.60% (<0.001) | Yes |
| United Kingdom | 523.97% | 28.23% (<0.01) | -12.63% (<0.001) | No |

15.06.2023

**Supplementary material 4 – Resilience, resistance, recovery and robustness of fundamental rights and freedoms**

| Countries | Cumulative resilience | Resistance | Recovery | Robustness |
|---|---|---|---|---|
| Austria | 1346,44 | 19.47 (<0.05) | -3.75 (<0.001) | No |
| Belgium | 1665,90 | 7.68 (<0.01) | -2.54 (<0.001) | No |
| Czechia | 1356,05 | 7.51 (<0.001) | -3.51 (<0.001) | No |
| Germany | 1638,86 | 6.61 (<0.001) | -1.05 (<0.01) | No |
| Denmark | 1531,12 | 16.53 (<0.001) | -1.17 (<0.001) | No |
| Spain | 1748,22 | 8.64 (<0.001) | -5.42 (<0.05) | No |
| Estonia | 1082,11 | 20.24 (<0.05) | -4.14 (<0.001) | No |
| Finland | 1234,17 | 6.54 (<0.001) | -2.51 (<0.001) | No |
| France | 1694,83 | 8.52 (<0.001) | -4.98 (<0.001) | No |
| Greece | 1590,63 | 17.06 (<0.001) | -4.59 (<0.001) | No |
| Hungary | 1533,83 | 15.08 (<0.001) | -1.33 (<0.001) | No |
| Iceland | 1137,53 | 4.25 (<.001) | -2.79 (0.001) | No |
| Italy | 1899,23 | 8.65 (<0.001) | -5.96 (<0.05) | No |
| Netherlands | 1478,14 | 15.59 (<0.01) | -3.60 (<0.001) | No |
| Norway | 1286,62 | 8.02 (<0.001) | -2.22 (<0.001) | No |
| Poland | 1517,30 | 7.31 (<0.001) | -4.63 (<0.01) | No |
| Portugal | 1842,75 | 7.66 (<0.001) | -1.80 (<0.001) | No |
| Slovakia | 1396,71 | 9.03 (<0.001) | -2.87 (<0.001) | No |
| Slovenia | 1370,70 | 19.91 (<0.001) | -6.47 (<0.001) | No |
| Sweden | 991,859 | 9.10 (<0.001) | -0.62 (<0.01) | No |
| Switzerland | 1417,79 | 15.54 (<0.01) | -3.64 (<0.001) | No |
| United Kingdom | 1771,10 | 6.09 (<0.01) | -0.76 (<0.05) | No |

**Supplementary material 5 – Box plots and whisker for robustness in economic system in 22 European countries based on GDP growth (annual %) from 2015 – 2020**

Data were collected from https://data.worldbank.org/indicator/NY.GDP.MKTP.KD.ZG. The method used for the detection of outliers is the same as for supplementary material 2. All countries had an outlier value for 2020 based on the 1,5 interquartile range method.

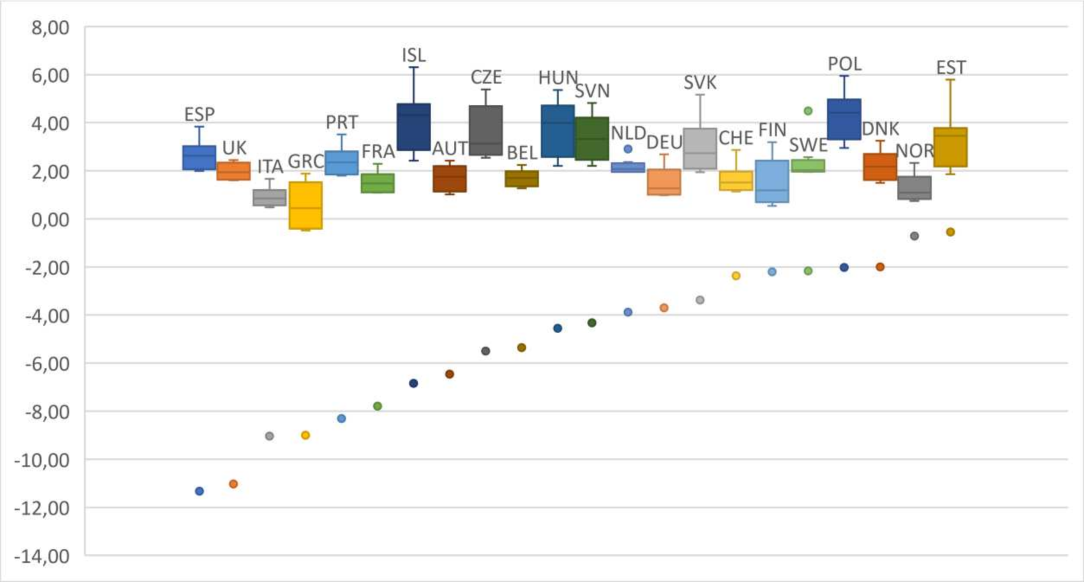


Abbreviations: Austria AUT, Belgium BEL, Czechia CZE, Denmark DNK, Estonia EST, Finland FIN, France FRA, Germany DEU, Greece GRC, Hungary HUN, Iceland ISL, Italy ITA, Netherlands NLD, Norway NOR, Poland POL, Portugal PRT, Slovak Republic SVK, Slovenia SVN, Spain ESP, Sweden SWE, Switzerland CHE, United Kingdom UK.

15.06.2023

## Supplementary material 6 - Economic system resilience, resistance, recovery and robustness

| Economic system (points/week) | | | | |
|---|---|---|---|---|
| **Countries** | **Cumulative resilience** | **Resistance rate** | **Recovery rate** | **Robustness** |
| Austria | -223,93 | -0.84 (<0.001) | 0.69 (<0.001) | No |
| Belgium | -221,94 | -0.91 (<0.001) | 0.82 (<0.001) | No |
| Czechia | -217,82 | -1.15 (<0.001) | 0.43 (<0.001) | No |
| Germany | -189,65 | -0.94 (<0.001) | 0.59 (<0.001) | No |
| Denmark | -93,66 | -0.30 (<0.001) | 0.27 (<0.001) | No |
| Spain | -450,65 | -1.37 (<0.01) | 0.94 (<0.001) | No |
| Estonia | -144,06 | -0.61 (<0.001) | 0.26 (<0.001) | No |
| Finland | -105,27 | -0.44 (<0.001) | 0.29 (<0.001) | No |
| France | -342,62 | -1.64 (<0.001) | 1.13 (<0.001) | No |
| Greece | -246,33 | -1.00 (<0.001) | 0.30 (<0.001) | No |
| Hungary | -251,55 | -1.51 (<0.001) | 0.66 (<0.001) | No |
| Iceland | -153,08 | -0.76 (<0.001) | 0.33 (<0.001) | No |
| Italy | -341,61 | -1.57 (<0.001) | 0.98 (<0.001) | No |
| Netherlands | -205,86 | -0.85 (<0.001) | 0.46 (<0.001) | No |
| Norway | -95,82 | -0.40 (<0.01) | 0.32 (<0.001) | No |
| Poland | -127,16 | -0.76 (<0.001) | 0.47 (<0.001) | No |
| Portugal | -325,42 | -1.21 (<0.05) | 0.89 (<0.001) | No |
| Slovakia | -284,89 | -13.76 (n/a) | 0.37 (<0.001) | No |
| Slovenia | -269,03 | -17.17 (n/a) | 0.55 (<0.001) | No |
| Sweden | -172,21 | -0.71 (<0.001) | 0.44 (<0.001) | No |
| Switzerland | -116,29 | -0.68 (<0.001) | 0.44 (<0.001) | No |
| United Kingdom | -388,33 | -1.68 (<0.001) | 1.01 (<0.001) | No |



Clausin M, et al. BMJ Open 2023; 13:e065445. doi: 10.1136/bmjopen-2022-065445

15.06.2023

**Supplementary material 7 – Table of Z-scores for liberal democracy index, stringency of the response, and excess mortality and two metrics based on these three Z-scores**

The methodology used to produce this table is described in supplementary material 1.

| Country | Excess-mortality | Stringency index | Liberal democracy index | Difference between stringency index and excess mortality | Difference between liberal democracy and stringency index |
|---|---|---|---|---|---|
| Austria | 2.097 | 1.449 | 1.086 | -0.648 | -0.363 |
| Belgium | 3.205 | 2.755 | 0.545 | -0.450 | -2.210 |
| Czechia | 1.654 | 1.489 | 1.426 | -0.166 | -0.062 |
| Denmark | 1.307 | 2.204 | 0.000 | 0.898 | -2.204 |
| Estonia | 1.550 | 0.369 | 0.404 | -1.181 | 0.035 |
| Finland | 2.292 | 0.991 | 0.559 | -1.302 | -0.432 |
| France | 3.013 | 2.874 | 0.712 | -0.140 | -2.161 |
| Germany | 1.582 | 2.645 | 0.471 | 1.063 | -2.173 |
| Greece | 1.990 | 2.448 | 1.196 | 0.458 | -1.252 |
| Hungary | 0.610 | 2.215 | 4.526 | 1.606 | 2.310 |
| Iceland | 0.000 | 0.595 | 0.967 | 0.595 | 0.371 |
| Italy | 3.012 | 3.709 | 0.899 | 0.697 | -2.810 |
| Netherlands | 3.117 | 1.988 | 0.567 | -1.129 | -1.421 |
| Norway | 0.674 | 1.205 | 0.280 | 0.531 | -0.924 |
| Poland | 2.026 | 2.148 | 2.815 | 0.122 | 0.667 |
| Portugal | 2.921 | 3.478 | 0.426 | 0.557 | -3.053 |
| Slovak Republic | 1.260 | 1.655 | 1.615 | 0.395 | -0.040 |
| Slovenia | 2.091 | 1.549 | 1.060 | -0.543 | -0.488 |
| Spain | 3.301 | 3.092 | 1.090 | -0.209 | -2.002 |
| Sweden | 2.940 | 0.000 | 0.000 | -2.940 | 0.000 |
| Switzerland | 2.073 | 1.741 | 0.306 | -0.332 | -1.435 |
| United Kingdom | 3.343 | 3.185 | 0.883 | -0.158 | -2.302 |

15.06.2023

**Supplementary material 8 – Difference between liberal democracy and stringency index (y axis) as a function of the difference between stringency index and excess mortality (x axis) for 22 European countries during the first wave of the COVID-19 pandemic in early 2020.**

The methodology used to produce this figure is covered in supplementary materials 1 and 4.

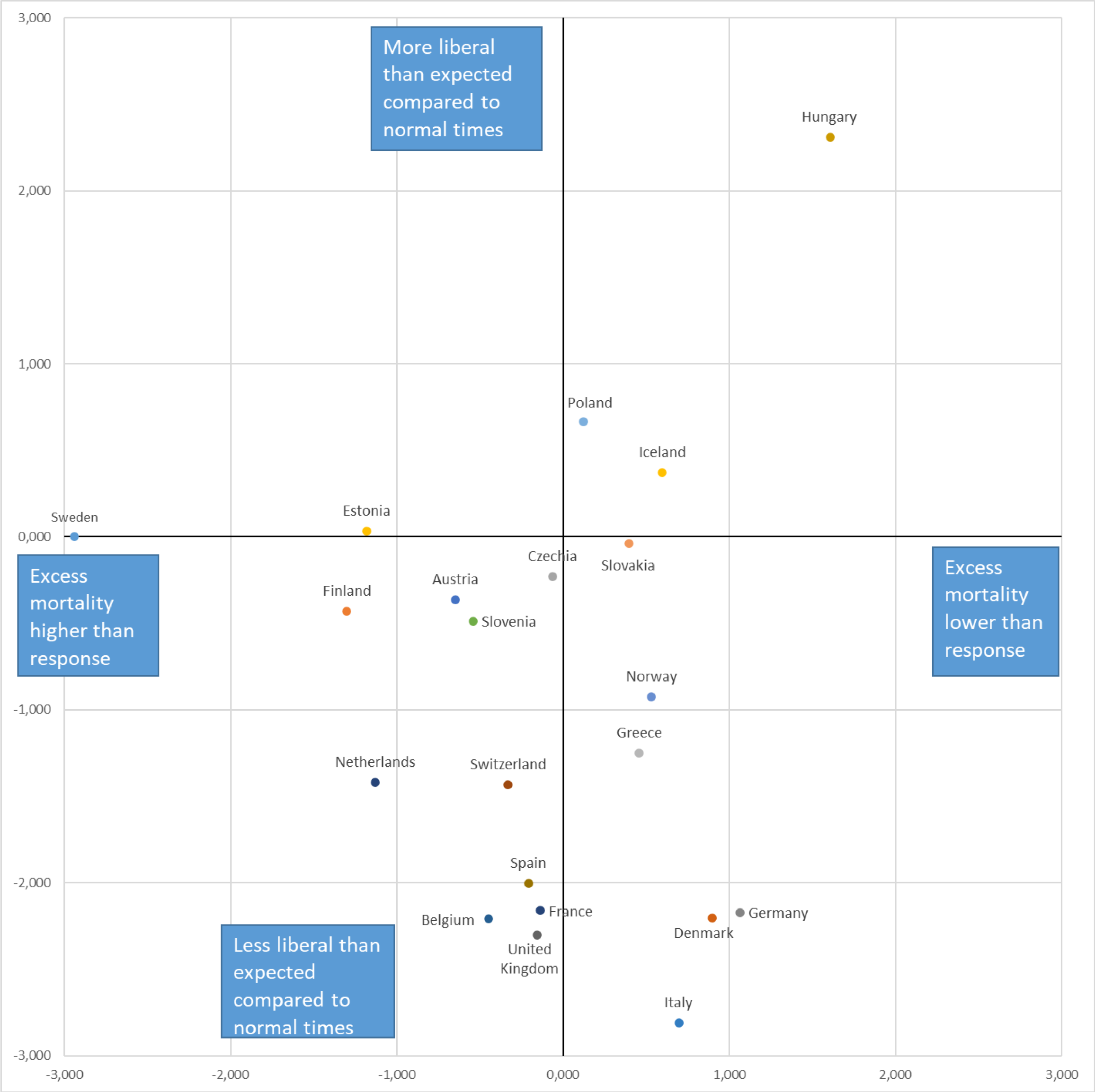




Clausin M, *et al. BMJ Open* 2023; 13:e065445. doi: 10.1136/bmjopen-2022-065445
