# Supplementary material for: Multisystemic resilience to shocks: a temporal analysis of health, fundamental rights and freedoms, and economic resilience during the first wave of the COVID-19 pandemic in 22 European countries
Source: BMJ Open. 2023 Jul 7;13(7):e065445. doi: 10.1136/bmjopen-2022-065445 (PMC10335565; doi:10.1136/bmjopen-2022-065445)
Supplement: Author's manuscript [file bmjopen-2022-065445.draft_revisions.pdf]

BMJ Open

BMJ Open is committed to open peer review. As part of this commitment we make the peer review history of every article we publish publicly available.

When an article is published we post the peer reviewers' comments and the authors' responses online. We also post the versions of the paper that were used during peer review. These are the versions that the peer review comments apply to.

The versions of the paper that follow are the versions that were submitted during the peer review process. They are not the versions of record or the final published versions. They should not be cited or distributed as the published version of this manuscript.

BMJ Open is an open access journal and the full, final, typeset and author-corrected version of record of the manuscript is available on our site with no access controls, subscription charges or pay-per-view fees (http://bmjopen.bmj.com).

If you have any questions on BMJ Open's open peer review process please email info.bmjopen@bmj.com

BMJ Open

# Measuring multisystemic resilience to shocks: health, fundamental rights and freedoms, and economic resilience during the COVID-19's first wave in Europe.



| | |
|---|---|
| Journal: | *BMJ Open* |
| Manuscript ID | bmjopen-2022-065445 |
| Article Type: | Original research |
| Date Submitted by the Author: | 24-Jun-2022 |



| | |
|---|---|
| Complete List of Authors: | Clausin, Mia; University of Geneva, Global Studies Institute<br>Rieckhoff, Alicia; University of Geneva, Global STudies Institute<br>Tediosi, Fabrizio; Swiss Tropical and Public Health Institute, Household Economics & Health Systems Research Unit<br>Morel, Chantal; University of Geneva, Global Studies Institute<br>Kaspiarovich, Yuliya; University of Geneva, Global Studies Institute<br>Levrat, Nicolas; University of Geneva, Global Studies Institute; Faculty of Law, University of Geneva<br>Wernli, Didier; University of Geneva |



| | |
|---|---|
| Keywords: | COVID-19, PUBLIC HEALTH, INFECTIOUS DISEASES |
| | |


SCHOLARONE™
Manuscripts

*I, the Submitting Author has the right to grant and does grant on behalf of all authors of the Work (as defined in the below author licence), an exclusive licence and/or a non-exclusive licence for contributions from authors who are: i) UK Crown employees; ii) where BMJ has agreed a CC-BY licence shall apply, and/or iii) in accordance with the terms applicable for US Federal Government officers or employees acting as part of their official duties; on a worldwide, perpetual, irrevocable, royalty-free basis to BMJ Publishing Group Ltd ("BMJ") its licensees and where the relevant Journal is co-owned by BMJ to the co-owners of the Journal, to publish the Work in this journal and any other BMJ products and to exploit all rights, as set out in our licence.*

*The Submitting Author accepts and understands that any supply made under these terms is made by BMJ to the Submitting Author unless you are acting as an employee on behalf of your employer or a postgraduate student of an affiliated institution which is paying any applicable article publishing charge ("APC") for Open Access articles. Where the Submitting Author wishes to make the Work available on an Open Access basis (and intends to pay the relevant APC), the terms of reuse of such Open Access shall be governed by a Creative Commons licence – details of these licences and which Creative Commons licence will apply to this Work are set out in our licence referred to above.*

*Other than as permitted in any relevant BMJ Author's Self Archiving Policies, I confirm this Work has not been accepted for publication elsewhere, is not being considered for publication elsewhere and does not duplicate material already published. I confirm all authors consent to publication of this Work and authorise the granting of this licence.*

For peer review only

24.06.2022

# Measuring multisystemic resilience to shocks: health, fundamental rights and freedoms, and economic resilience during the COVID-19's first wave in Europe.

Mia Clausin[1*], Alicia Rieckhoff[1*], Fabrizio Tediosi[2-3], Chantal Morel[1], Yuliya Kaspiarovich[1], Nicolas Levrat[1], Didier Wernli[1]

[1] Geneva Transformative Governance Lab, Global Studies Institute, University of Geneva, Switzerland
[2] Swiss Tropical and Public Health Institute, Basel, Switzerland
[3] University of Basel, Basel, Switzerland

* These authors contributed equally.

Keywords: resilience, robustness, recovery multisystemic, health systems, governance, complexity, systemic risks, systemic crisis, COVID-19

Corresponding author: Didier.Wernli@unige.ch

Word count: 6848 in the main text. 3 tables, 3 figures, 1 supplementary material. 25 references.

Abstract:

**Objectives:** While resilience research on COVID-19 has primarily focused on health system resilience, the purpose of this paper is to measure 'multisystemic' resilience (in the health, economic, and fundamental rights and freedoms dimensions) of 22 European countries and resilience dynamics during the first wave of the COVID-19 pandemic by assessing different components of resilience.

**Methods/Design:** Multisystemic resilience was assessed through time-series analyses in 22 European countries of the effects generated by the first wave of the COVID-19 pandemic in three sectors: health, economy, and fundamental rights and freedoms. An overall (cumulative) resilience was estimated, as well as three of its sub-components: robustness, resistance, and recovery.

**Participants:** This study is based on an online search of publicly available data. No patient, nor the public were involved in the design of the study.

**Results:** While only 9 countries exceeded their excess mortality (P-score) of previous years (2015-2019) during the first wave of the COVID-19 pandemic, all countries crossed their previous economic minimum thresholds and implemented various restrictions levels. The main findings suggest that the resilience of one system influence other sectors' resilience. Overall health resilience was associated with economic resilience ($r(22) = .69$, $p < .001$); Health resilience was associated with overall fundamental rights and freedoms resilience but to a lesser extent ($r(22) = .56$, $p = .007$). Economic resilience was found to be positively linked to both health as well as fundamental rights and freedoms resilience ($r(22) = .67$, $p < .001$).

**Conclusions:** This paper provides a novel approach for the systematic assessment of multisystemic resilience to gauge the strengths and shortcomings of countries' capacities to tackle systemic crises.



For peer review only - http://bmjopen.bmj.com/site/about/guidelines.xhtml

24.06.2022

Strengths and limitations of this study:

- First study to operationalize the resilience of 22 European countries to the first wave of the COVID-19 pandemic in three sectors – health, economy, and fundamental rights and freedoms.
- Three resilience components - robustness, resistance, and recovery - in addition to an overall measure of resilience have been measured, and different measurements methods have been tested.
- The primary limitation when assessing resilience lies in country comparability (countries were not exposed to COVID-19 in the same way) and the interpretation of resilience.
- Selected indicators only partially capture the resilience, overlooking other factors influencing countries' ability to absorb and cope with the shock.
- While measurement of resilience remains in need of further research, the various measurement methods tested showed significant variations in results.

## INTRODUCTION

The COVID-19 pandemic has tested the resilience of countries to deal with a macro-shock affecting health, economic, social, environmental, and governance systems worldwide. In an early 21st century increasingly characterized by uncertainty, complexity, and systemic disruptions, resilience thinking has gained traction in various fields including disaster management, the environment, and more recently global health [1, 2]. While research on resilience to COVID-19 primarily focuses on health systems, [3-5], several studies have called for the operationalization of resilience to go beyond the functions of the health system in response to shocks [6]. Considering resilience as a multi-objective societal problem, the concepts of 'systemic resilience' [7] and 'multisystemic resilience' [8] have emerged to better understand the impact of shocks across highly interconnected systems.

There are currently different definitions of resilience across fields ranging from the capacity of a system to come back to its initial state to the capacity *"to absorb disturbance and reorganise while undergoing change so as to still retain essentially the same function, structure, identity, and feedbacks"* [9]. Resilience thinking therefore draws attention to both the vulnerability of systems and the capacity to respond. However, with no universally accepted definition bridging sectoral divides and limited methodological development across disciplines, the measurement of resilience remains underdeveloped [10]. Stemming from recent works, resilience as a property of a complex system can be measured according to three characteristics: robustness, resistance, and recovery [11, 12]. The main idea is that resilience can be expressed in different ways according to the stage of an endogenous disruption or exogenous shock.

While the policy literature has also sought to operationalize measurement of multisystemic resilience [13], this paper aims to operationalize multisystemic resilience and provide a practical application of how to measure resilience and its dynamics over the first wave of the COVID-19 pandemic by assessing three components of resilience – robustness, resistance, and recovery [11]. This is a cross-country comparative study depicting the first wave of the COVID-19 pandemic in 22 European countries through multi-component time-series analyses, measuring health resilience, economic resilience and resilience of fundamental rights and freedoms. While the pandemic is not over, this study serves as a methodological contribution to better understand how different systems are affected by the COVID-19 crisis and impact each other, to mitigate undesirable knock-on effects in the long-run. Understanding resilience as the ability of societies to maintain their core governance functions while



For peer review only - http://bmjopen.bmj.com/site/about/guidelines.xhtml

24.06.2022

minimising the undesirable societal effects can serve as the foundation for an integrated approach to building societal resilience to future pandemics [14].

# METHODS

## Public and Patient Involvement

This study is based on online search of publicly available webpages and documents. No patient, nor the public were involved in the design of the study. No ethical approval was sought as per our university requirements.

## Study design for time-series analyses

The proxy measures for health, fundamental rights and freedoms, and economic resilience were selected using two criteria: first, the availability of weekly or monthly indicators which would allow for a detailed time-series analysis; second, their relevance to capture the type of resilience at hand. Table 1 outlines the definitions we used for resilience and its 3 components across the different sectors. Data was collected between week 3 when no measures were implemented, to week 35 when COVID-19 death near 0 for all European countries (13.01.2020-31.08.2020). Twenty-two European countries were chosen according to the availability of data across the selected dimensions of resilience.

The indicator selected for measuring health resilience was weekly excess mortality (P-score), sourced from Our World in Data COVID-19 dataset [15]. A death is recorded by week of occurrence, apart from the UK, where death by week of registration is used. Excess mortality was chosen as it helps overcome several issues related to the reporting of COVID-19 related deaths such as miscounting from misdiagnosis or under-reporting. It also includes 'collateral damage' from other health conditions left untreated if the health system is overwhelmed [5]. Excess mortality was defined as the number of deaths registered in excess of the five-year average from 2015 to 2019. The calculations for Germany and Greece were based on the four-year average (2016-2019) due to unavailable data. A weekly P-score, which was chosen as it allows for robust country comparisons, was calculated as the percentage difference between the reported and projected number of deaths. Health resilience was interpreted as the inverse of excess mortality. The lower the excess mortality, the larger health resilience.

The indicator selected for the resilience of fundamental rights and freedoms was the Oxford Coronavirus Government Response Tracker's (OxCGRT) stringency index [16]. The stringency index records the strictness of "lockdown style" policies that primarily restrict people's behaviour. The index is a composite measure of categorical variables based on nine indicators: school closing, workplace closing, cancelling of public events, restrictions on the size of gatherings, closing of public transport, stay-at-home requirements, restrictions on internal movement, restrictions on international travel and public information campaigns. It therefore provides a proxy indicator to measure restrictions in fundamental rights and freedoms, considering that the latter (e.g., freedom of movement; right to assembly and demonstration) are intrinsic to the functioning of liberal democracies. While the indicator is a daily value between 1 and 100 (100 = strictest), a weekly average was derived for this study. Resilience of fundamental rights and freedoms was measured as the inverse of the stringency index. This means that the more stringent was the response, the less resilient were fundamental rights and freedoms.

The economic indicator selected was the OECD's short-term main economic indicator: GDP ratio to trend [17]. This indicator is a monthly derived indicator where GDP is divided by the long-term GDP



For peer review only - http://bmjopen.bmj.com/site/about/guidelines.xhtml

24.06.2022

trend to give a ratio-to-trend. It is notably useful to visualise economic cycles. If the GDP ratio to trend = 100, GDP is equal to long-term GDP trends. If the GDP ratio to trend is higher than 100, GDP is higher than long-term GDP trends which signified economic expansion. If the GDP ratio to trend is below 100, the GDP is lower than the long-term GDP trend signalling a contraction. GDP ratio to trend data were compared to quarterly GDP, i.e., the percentage change from the same quarter in previous years, to check for its accuracy in representing the magnitude of the shock. Economic resilience to the shock was interpreted as the inverse of the decline of the GDP ratio.

*Table 1 Resilience to COVID-19 and its applications to health, fundamental rights and freedoms, and the economy based on three core subcomponents of resilience (inspired by Grafton et al. [11])*

| Definitions | Health system | Fundamental rights and freedoms | Economic system |
|---|---|---|---|
| **Resilience** is the capacity of a system to withstand, absorb, and recover from disturbance. It can be sub-divided into its three components: robustness, resistance, and recovery. | Capacity to meet significant increases in demand for both public health and healthcare services and adapt to long-standing changes. | Capacity to preserve and protect individual rights and freedoms while maintaining the respect for the rule of law. | Capacity to limit the magnitude of economic losses, recovery quickly, and forge new developmental paths for prosperity. |
| ***Robustness*** is probability of a system to maintain its identity and not cross an undesirable threshold following the adverse event. | Capacity to maintain the population health within the range of normal variation. | Capacity to maintain respect for individual rights and freedoms within the range of normal variation. | Capacity to maintain economic indicators performance within the range of normal variation. |
| ***Resistance*** is the capacity to absorb disruption with minimal damage to system functionality. | Capacity to slow down COVID-19 transmission and excess mortality. | Capacity to minimize an escalation of stringent measures violating fundamental rights and freedoms. | Capacity to minimize economic disruption and impact of the shock. |
| ***Recovery*** is capacity to 'bounce-back' following disruption. | Capacity to recover to a scenario of pre-pandemic excess mortality rates. | Capacity to end emergency measures that impact fundamental rights and freedoms. | Capacity to recover to a scenario with pre-pandemic GDP trends and avoid a long-term recession. |

## Measuring resilience, robustness, resistance, and recovery

The conceptual framework to measure resilience and its sub-components is visualised in Figure 1. Table 2 outlines the criteria used to measure health, fundamental rights and freedoms, and economic resilience, robustness, resistance, and recovery. Overall resilience in health, fundamental rights and freedoms and the economy was estimated using the inverse of the area under the curve during the study period. It was calculated by adding the area of data points until the horizontal axis (when x=0 for excess mortality and stringency index, or x=100 for GDP ratio to trend) and subtracting the area of data points beyond the horizontal axis (when x<0 for excess mortality and x>100 for GDP ratio to trend). Data points used to calculate the area under the GDP ratio to trend curve were computed as the point difference to trend (if x=97, then 100-97=3 was coded). Results were then used to conduct a comparative analysis across sectors and identify general trends and potential trade-offs between them. This allowed us to analyse the resilience level relative to other sectors' results and minimise bias caused by different time exposure to the virus from one country population to another. The greater the difference between two sectors, the more resilient it was considered in that domain.



For peer review only - http://bmjopen.bmj.com/site/about/guidelines.xhtml

24.06.2022

***Table 2*** *Measurements of resilience, robustness, resistance, and recovery across the health - fundamental rights and freedoms, and economic spectrum*

| | Resilience | Robustness | Resistance | | Recovery | |
|---|---|---|---|---|---|---|
| | | | rate | time | rate | time |
| Health | Inverse of the area under the excess mortality curve using the trapezoid rule between 13.01.20 – 31.08.20 | date of crossing the threshold of 38% excess mortality | Inverse of slope of linear regression between (a) min to max and (b) 0 to max | time in weeks between 0 to max | slope of linear regression between (a) max to min and (b) max to 0 | time in weeks between peak and 0 |
| Fundamental rights and freedoms | Inverse of the area under the stringency index curve using the trapezoid rule between 13.01.20 – 31.08.20 | date of crossing the threshold of 9.4 on the stringency index | Inverse of the slope of linear regression between min (0) to max | time in weeks between min (0) to max | slope of linear regression between max and end of period (31.08.20) | n/a |
| Economic | Inverse of the area above GDP ratio-to-trend curve using the trapezoid rule between 13.01.20 – 31.08.20 | date of crossing the threshold of -1.25 of GDP ratio to trend | Inverse of the slope of linear regression between -1.25 to min | time in weeks between -1.25 to min | slope of linear regression between min and end of period (31.08.20) | n/a |

We defined robustness thresholds as the maximum or minimum value from previous years 2015-2019, based on the assumption that countries were 'robust' in all three sectors before the COVID-19 first wave. Using the definition of health robustness as the capacity to maintain all health services [5], the maximum value of 38%, which reflects the level of excess mortality in Germany in 2018) was set as the health robustness threshold (representing 24% of the 2020 maximum). Based on the same approach, a threshold of -1.25 was defined as the economic robustness threshold (representing 5.5% of the 2020 maximum). For the covid-19 specific stringency index, the value of 9.4 (or 10% of the 2020 maximum) was arbitrarily set as our fundamental rights and freedoms robustness threshold. Dates of crossing the threshold in all three dimensions were collected and compared with each other. Finally, for comparative purposes between the three sectors within the same country, robustness was estimated as the time required to cross a specific threshold to measure temporal lags between sectors. The goal was to limit biases caused by timing differences in virus occurrence between countries.

Resistance was estimated using the slope of linear regressions from the interval of data from a minimum to the next maximum or peak [3]. A higher slope value (in %) represents a lower level of resistance. For the health resistance, the slope of the data interval from 0 to the next maximum was also calculated (instead of the minimum) to limit the effect of non-pandemic related fluctuations regarding excess mortality. For economic resistance, the interval was calculated from the threshold of -1.25 instead of the maximum, to prevent the inclusion of economic fluctuations due to other factors (the determination of the threshold is explained under the robustness calculation). For all countries, except for Slovenia and Slovakia (1 week), this interval was 10 weeks. Due to this large interval discrepancy, Slovenia and Slovakia have therefore been excluded from the correlation coefficient analysis using economic resistance results.

Recovery was measured using the slope of the linear regression over the data interval between the maximum and minimum or the end of the time series if earlier. A higher negative slope value (in %) represents a faster and higher level of recovery. For the health recovery estimation, we also calculated the slope between the maximum and 0 (instead of the minimum). This allowed us to avoid including



For peer review only - http://bmjopen.bmj.com/site/about/guidelines.xhtml

24.06.2022

time intervals when countries slowly stabilized near their minimum but continued to decline. For the economic and fundamental rights and freedoms recovery, the interval ranged from the maximum to the end of the time series (31.08.20).

Twelve countries were removed from the calculation of health resistance, recovery, and robustness due to no sizeable mortality peak/maximum, too many minor peaks, or where the study period did not cover the entire peak. The ten countries kept were Sweden, Spain, Portugal, Italy, Belgium, Switzerland, Germany, France, Great Britain, and the Netherlands. Correlation coefficients were calculated between the different sectors as well as between resilience's components to evaluate potential association trends. Various correlation tables were computed and tested: (1) with all countries (n=22) for the association among overall health resilience, resilience of fundamental rights and freedom and economic resilience, (2) with only 9 countries showing a mortality peak for correlations between health resilience and health resistance and recovery, and (3) without SVK and SVN for correlations between economic resilience and economic resistance and recovery.

## RESULTS

We present the results by systems and then from a multisystemic perspective. Table 3 synthesizes the results of the measurements of the three components of resilience (i.e., robustness, resistance, and recovery) for health, fundamental rights and freedoms, and economic dimensions.

**Table 3** Overall results regarding the measurments of resilience (darker shade of colours indicates countries that had the lowest expressed capacities)

| COUNTRY | **AREA UNDER THE CURVE (Overall resilience corresponds to the inverse of these values)** | | | **RESISTANCE** slope from 0 - max *(from min - max)* | **RECOVERY** slope from 0 - max (from min - max) | **ROBUSTNESS** date of crossing the threshold 38% | **ROBUSTNESS** temporal lag between sectors *(health as reference point)* | **RESISTANCE** slope from min - max | **RECOVERY** slope max - min | **ROBUSTNESS** date of crossing the threshold 9,4 | **ROBUSTNESS** temporal lag between sectors *(health as reference point)* | **RESISTANCE** slope from -1,25 - min | **RECOVERY** slope from min - max | **ROBUSTNESS** date of crossing the threshold -1,25 | **ROBUSTNESS** temporal lag between sectors *(health as reference point)* |
|---|---|---|---|---|---|---|---|---|---|---|---|---|---|---|---|
| | **HEALTH** | **FUND. RIGHTS & FREEDOMS** | **ECONOMIC** | **HEALTH** | | | | **FUND. RIGHTS & FREEDOMS** | | | | **ECONOMIC** | | | |
| **CZE** | 0,46 | 1337,99 | 216,23 | n/a | n/a | n/a* | n/a | 7,51 | -2,10 | 27.01.2020 | n/a | -6,55 | 4,08 | 24.02.2020 | n/a |
| **EST** | 0,23 | 1072,39 | 142,02 | n/a | n/a | n/a* | n/a | 20,24 | -3,47 | 09.03.2020 | n/a | -6,07 | 2,14 | 24.02.2020 | n/a |
| **HUN** | -0,92 | 1509,30 | 248,35 | n/a | n/a | n/a* | n/a | 15,08 | -1,47 | 02.03.2020 | n/a | -15,12 | 6,23 | 24.02.2020 | n/a |
| **POL** | 1,04 | 1497,40 | 126,14 | n/a | n/a | n/a* | n/a | 7,14 | -2,98 | 02.03.2020 | n/a | -10,09 | 4,35 | 24.02.2020 | n/a |
| **SVK** | -0,07 | 1382,10 | 281,83 | n/a | n/a | n/a* | n/a | 9,03 | -2,87 | 02.03.2020 | n/a | -137,62 | 2,77 | 24.02.2020 | n/a |
| **SVN** | 0,91 | 1347,55 | 268,03 | n/a | n/a | n/a* | n/a | 19,91 | -2,34 | 02.03.2020 | n/a | -171,66 | 3,37 | 24.02.2020 | n/a |
| **DNK** | -0,03 | 1505,65 | 92,58 | n/a | n/a | n/a* | n/a | 16,53 | -1,09 | 27.02.2020 | n/a | -3,84 | 2,53 | 24.02.2020 | n/a |
| **FIN** | 1,03 | 1217,97 | 103,78 | n/a | n/a | n/a* | n/a | 6,54 | -1,72 | 03.02.2020 | n/a | -6,61 | 2,75 | 23.03.2020 | n/a |
| **ISL** | -0,41 | 1117,62 | 150,88 | n/a | n/a | n/a* | n/a | 4,25 | -0,93 | 27.01.2020 | n/a | -11,22 | 3,19 | 23.03.2020 | n/a |
| **NOR** | -0,32 | 1270,88 | 95,36 | n/a | n/a | n/a* | n/a | 8,02 | -2,46 | 03.02.2020 | n/a | -4,00 | 3,00 | 24.02.2020 | n/a |
| **SWE** | 2,84 | 973,34 | 169,98 | 13,8% *(7,90%)* | -3,74% *(-3,52%)* | 03.04.2020 | 0 | 9,10 | -0,62 | 09.03.2020 | -4,5 | -7,82 | 4,13 | 24.02.2020 | -6,5 |
| **ESP** | 6,49 | 1716,97 | 445,61 | 45,3% *(30,73%)* | -26,23% *(-14,35%)* | 13.03.2020 | 0 | 8,64 | -1,49 | 03.02.2020 | -5,5 | -13,72 | 8,98 | 24.02.2020 | -2,5 |
| **GRC** | 1,16 | 1562,62 | 241,37 | n/a | n/a | n/a* | n/a | 17,06 | -1,83 | 24.02.2020 | n/a | -15,39 | 2,81 | 23.03.2020 | n/a |
| **ITA** | 4,02 | 1871,92 | 339,49 | 23,68% *(8,64%)* | -10,77% *(-8,02%)* | 09.03.2020 | 0 | 9,32 | -1,82 | 27.01.2020 | -6 | -8,53 | 9,35 | 24.02.2020 | -2 |
| **PRT** | 3,01 | 1814,42 | 323,36 | 10,01% *(10,01%)* | -7,99% *(-7,99%)* | 10.07.2020 | 0 | 7,66 | -1,00 | 27.01.2020 | | -12,08 | 8,46 | 24.02.2020 | |
| **AUT** | 1,06 | 1330,68 | 221,38 | n/a | n/a | n/a* | n/a | 19,47 | -2,87 | 24.02.2020 | n/a | -9,19 | 6,52 | 24.02.2020 | n/a |
| **BEL** | 4,01 | 1639,25 | 221,81 | 29,89% *(19,48%)* | -12,92% *(-12,92%)* | 20.03.2020 | 0 | 7,68 | -1,69 | 03.02.2020 | -6,5 | -9,05 | 7,81 | 24.02.2020 | -3,5 |
| **CHE** | 1,11 | 1395,38 | 116,10 | 12,81% *(11,23%)* | -9,59% *(-6,23%)* | 27.03.2020 | 0 | 15,54 | -1,82 | 24.02.2020 | -4,5 | -5,20 | 4,14 | 24.02.2020 | -4,5 |
| **DEU** | 0,44 | 1609,00 | 188,16 | 6,9% *(6,98%)* | -9,21% *(not cross. 0)* | n/a* | n/a | 6,61 | -1,05 | 17.02.2020 | n/a | -8,97 | 5,60 | 24.02.2020 | n/a |
| **FRA** | 2,53 | 1674,69 | 341,09 | 17,3% *(11,95%)* | -2,24% *(-2,24%)* | 23.03.2020 | 0 | 8,52 | -2,99 | 27.01.2020 | -8 | -9,39 | 10,76 | 24.02.2020 | -4 |
| **GBR** | 5,20 | 1738,00 | 383,63 | 23,5% (10,26%) | -12,63% *(-11,52%)* | 27.03.2020 | 0 | 6,09 | -0,76 | 03.02.2020 | -7,5 | -18,13 | 9,55 | 24.02.2020 | -4,5 |
| **NLD** | 3,31 | 1454,53 | 203,32 | 18,86% *(14,24%)* | -6,45% *(-6,45%)* | 20.03.2020 | 0 | 15,59 | -2,40 | 04.03.2020 | -2,3 | -7,65 | 4,39 | 24.02.2020 | -3,5 |

24.06.2022

## Health resilience

Up to 24 August 2020, 9 countries - Spain (max/peak = 158%), Great Britain (108%), Belgium (105%), Italy (85%), the Netherlands (75%), France (65%), Sweden (47%), Switzerland (45%), and Portugal (44%) - had exhibited at least one excess mortality peak and crossed the 38% of excess mortality robustness threshold during the first wave. The remaining countries (maximum values from 6% to 25%) did not experience a peak in excess mortality nor exceeded the excess mortality of previous years (2015-2019). Eight of the nine countries with a mortality peak - Spain (649), Great Britain (520), Italy (402), Belgium (401), the Netherlands (331), Portugal (301), Sweden (284), and France (253) - also had the highest cumulative excess mortality over the study period (area under the curve), suggesting a low overall health resilience to the first wave of the COVID-19 pandemic in spring 2020. Switzerland scored a much lower cumulative excess mortality (111). Five countries registered negative cumulative excess mortality - Hungary (-92), Iceland (-41), Norway (-32), and Slovakia (-7). The cumulative resilience average for the 22 countries was 168.64.

For most countries, the peak of excess mortality was reached in 4 or 5 weeks. However, recovery times differed greatly. Sweden (14 weeks), Great Britain (10 weeks), and Italy (7 weeks) took the longest time to recover. Spain and Belgium recovered relatively quickly despite their lower resistance (i.e., high value of the slope) to curtain their excess mortality increase. Health resistance and recovery were negatively correlated ($r(22) = -0.84$, $p < .001$) the lower the health resistance (higher peak), the faster the recovery. Spain showed the lowest resistance (45,28%) with excess mortality rates increasing by 6.0-fold in the week of the 9$^{th}$ of March and then almost tripling to 158.25% within two weeks. Yet, the country also showed the highest recovery rate (-26,23%). This pattern of low resistance/and quick recovery related to excess mortality was consistent across countries, in contrast to the economic and fundamental rights and freedoms dimensions.

For the 9 countries with a peak, both low level of resistance ($r(9) = .85$, $p < .001$), and high level of recovery ($r(9) = .78$, $p < .005$), were associated with overall cumulative excess mortality and faster recovery (since only peaking countries showed high recovery rates), thus highlighting the importance of recovery capacity for the overall country's health resilience during the first wave. Spain, with the lowest resistance and highest recovery, also had the lowest cumulative health resilience (649). Yet, low resistance does not necessarily imply low overall resilience. Switzerland exhibited a mortality peak and rather low resistance of 12.81% but with a cumulative excess mortality of only 111, similar to other non-peaking countries like Austria (106) or Greece (116). This can be explained by the country's high recovery rate (-9.60%). With a resistance close to Switzerland's, Sweden (13.8%) had a lower recovery rate (-3.74%) as well as significantly cumulative excel mortality (284), compatible with the implementation of less stringent government intervention. This further illustrates the importance of the recovery capacity on overall health resilience. Most excess mortality data were positively skewed (mean>median), testifying that high excess mortality occurred earlier in the period (usually March-April), rather than the summer where in most countries excess mortality declined apart from some countries which experienced a second peak (notably Belgium, Germany, The Netherlands, and Portugal).

## Resilience of fundamental rights and freedoms

All 22 countries implemented varying levels of restrictions and crossed the 9.4 robustness threshold during the first wave. The highest values of the stringency index were recorded by Italy (93.5), Slovenia (89.8) and France (88), while the lowest by Sweden (46.3), Iceland (53.5) and Finland (63). As of 24



For peer review only - http://bmjopen.bmj.com/site/about/guidelines.xhtml

24.06.2022

August 2020, no country had returned to its pre-pandemic situation. Italy (1871.9), Portugal (1814.4), Great Britain (1738), and Spain (1717) exhibited the highest cumulative stringency over the study period (area under the curve), suggesting a low resilience of fundamental rights and freedoms, while Sweden (973.3), Estonia (1072.4), Iceland (117.6), and Finland (1218) exhibited the lowest cumulative stringency, indicating higher fundamental rights and freedoms resilience. The average cumulative fundamental rights and freedoms resilience for all 22 countries was 1456.3. We observed that countries often maintained a high stringency level regardless of their epidemiological situation during the first wave, reflected in the low fundamental rights and freedoms recovery average of -1.9%, lower than health (-9.4%) and economic (5.3%) recovery rates.

Various countries reacted strongly at first, suggesting weak resistance, but were also quick to remove their restrictions, such as Estonia which had the lowest resistance rates (20.24%) (the fastest escalation of strict measures), as well as the highest recovery (-3.47%), and the second-highest overall fundamental rights and freedoms resilience (1072.4). This was reflected by positively skewed data, evident for France, Norway, Estonia, and the Czech Republic who adopted more stringent measures earlier on but were quick to remove them by the summer. In contrast, Sweden which recorded the highest cumulative resilience of fundamental rights and freedoms of all countries showed a moderate resistance (9.1%) principally due to late measure introduction but low recovery (-0.6%). Negatively skewed data was evident for Great Britain, Germany, Spain, Greece, and Sweden who imposed and maintained more stringent measures later in the period. Other countries escalated quickly (low resistance) and maintained severe government interventions during the whole period, indicating a low recovery, such as Hungary, Germany, Portugal, or Denmark. These mixed results reflect the different strategies adopted by countries to deal with the COVID-19 during the first wave.

## Economic resilience

All countries experienced an economic decline and crossed the robustness threshold of -1.25, followed by recovery during the first wave, although to varying extents. Spain (445.61), Great Britain (383.62), France (341.1), and Italy (339.5) recorded the greatest losses in terms of cumulative GDP to trend over the study period (area under the curve), while Denmark (93), Norway (95.4), Finland (103.8), and Switzerland (116.1) exhibited the lowest cumulative GDP losses. The cumulative resilience average for all 22 countries is 223.66. All countries started their economic downturn in late February, apart from Finland, Greece, and Iceland a month after. Most of them had positively skewed data - abrupt economic decline (low resistance) and slower recovery rates. For most countries, except Slovenia and Slovakia, it took 10 weeks to reach the economic trough. Slovenia (17.17%) and Slovakia (13.76%) experienced the quickest downturn (lowest resistance rates), yet they reached their minimum in just one week and thus were already starting to recover while other countries were still in economic decline. This points out the limitation of the maximum/minimum method, i.e., a country with a slope similar to Slovenia's during this week will have a lower overall slope (higher resistance) if it continues to decline slowly and thus if its maximum occurs later instead of recovering straight after this one-week decline like Slovenia did. Therefore, Slovenia and Slovakia were taken out of the correlation coefficient calculation. This explains why Slovakia recorded both the lowest economic resistance (-13.76%) and recovery (0.28%) but registered rather low cumulative economic losses (281.83); while Spain had the largest cumulative losses (445.6) although higher resistance (-1.37) and faster recovery (0.9%).



For peer review only - http://bmjopen.bmj.com/site/about/guidelines.xhtml

24.06.2022

Economic resistance ($r(20) = .69$, $p < .001$) and recovery ($r(20) = .78$, $p < .001$) were found to be associated with overall economic resilience: lower resistance (faster economic decline) and faster recovery resulted in higher cumulative GDP losses, thus demonstrating the importance of the recovery capacity in coping with the economic decline during the first wave. Countries with the highest resistance such as Denmark (-0.38%), Norway (-0.4%) and Switzerland (-0.52%) also had some of the highest overall economic resilience, highlighting the important role of resistance on overall economic resilience. Like the fundamental rights and freedoms dimension, no relationship was found between resistance and recovery; in other words, low economic resistance corresponded to a wide range of recovery rates. Greece registered low economic resistance (-1.54%) and had one of the slowest recovery rates (0.28%), while Hungary with a similar resistance (1.51%) had a faster recovery (0.62%), yet both had similar cumulative economic resilience. This may be related to biases in the resistance calculation outlined in the discussion. These non-uniform results may suggest that economic response capacity, unlike health response capacity, differed more greatly among countries.

## Multisystemic resilience

Cross-sectoral comparisons of resilience results (mainly overall resilience) across the health, fundamental rights and freedoms, and economic dimensions allowed us to identify patterns, potential linkages and trade-offs between sectors and thereby derive a multisystemic understanding of a country's resilience (Figure 2). Some countries may exhibit a high level of resilience in one domain but weak resilience in another. The time series graphs for all 22 countries combining measurements of the health, fundamental rights and freedoms, and economic dimensions of resilience are available in Supplementary file 1. Three countries - Iceland, Estonia, and Finland - were found to have high overall resilience in all three domains, closely followed by Norway, yet with slightly lower overall fundamental rights and freedoms resilience. On the other hand, Spain and Great Britain showed the lowest overall resilience in both the health and economic sectors but were surpassed by Portugal and Italy in overall fundamental rights and freedoms resilience. The comparative analysis shows more nuanced results with a significant tendency among all 22 European countries to favor health and economic sectors over fundamental rights and freedoms during the first wave of the COVID-19 pandemic in Europe in 2020, possibly related to the novel character of the pandemic and the importance given to the precautionary principle.

Of the nine countries with an excess mortality peak (low health resilience), Sweden, Spain, Great Britain, and the Netherlands presented the highest fundamental rights and freedoms resilience when compared with their excess mortality levels. Sweden showed the highest fundamental rights and freedoms resilience among all countries. On the other hand, France, Portugal, and Italy showed low fundamental rights and freedoms resilience relative to their epidemiological situation. Of the countries without an excess mortality peak, Hungary followed by Denmark, Germany, Greece, and Poland had the lowest fundamental rights and freedoms resilience to health. Switzerland, Sweden, the Netherland, and Belgium demonstrated a high economic resilience compared to their health results suggesting stronger economic resilience. Conversely, France and Portugal exhibited low economic resilience compared to their health situation, while Spain, Great Britain and Italy showed moderate economic resilience relative to health. Among the low or no excess mortality countries, Hungary, Slovenia, and Slovakia demonstrated the weakest economic resilience (sharp economic decline), while Poland and Finland the highest economic resilience relative to health.



For peer review only - http://bmjopen.bmj.com/site/about/guidelines.xhtml

24.06.2022

When comparing the fundamental rights and freedoms and the economic dimensions, more than half of the countries had lower cumulative economic losses (higher economic resilience) than fundamental rights and freedoms resilience. Poland and Switzerland registered the highest economic resilience compared to fundamental rights and freedoms resilience. This suggests that governments prioritized economic performance and that measures implemented to curtail the transmission of COVID-19 did not hinder countries' economies substantially. On the other hand, Spain and Sweden showed the highest fundamental rights and freedoms resilience compared to their economic situation. Similar findings were found by calculating correlation coefficients between the three sectors. Overall health resilience was associated with economic resilience ($r(22) = .69$, $p < .001$); in other words, countries with higher excess mortality also suffered greater economic losses. Health resilience appeared to be positively associated with overall fundamental rights and freedoms resilience but to a lesser extent ($r(22) = .56$, $p = .007$). This may be explained by the fact that many countries implemented stringent measures independently of their excess mortality trends. Overall economic resilience was found to be positively linked to both health as well as fundamental rights and freedoms resilience ($r(22) = .67$, $p < .001$). The association of the economic domain with both the health dimension and the stringency of adopted measures may indicate its heavy reliance on, and importance to, other sectors' resilience.

Comparison of robustness across three sectors demonstrates the temporal lag between them and its potential influence on overall resilience. Only nine countries (Spain, Great Britain, Italy, Belgium, Switzerland, Netherlands, Portugal, France, and Sweden) crossed the 38% robustness health threshold during the first wave. Although several countries were robust at the health system level, none of the 22 European countries were robust across all dimensions, as all 22 countries crossed economic and fundamental rights and freedoms robustness thresholds. This aligns with the idea that when resilience is provided by intervention (and not by design), there is transfer of capacities from other sectors [18]. Most countries crossed the fundamental rights and freedoms robustness threshold before experiencing economic decline and increase in excess mortality, suggesting that their fundamental rights and freedoms robustness levels tended to be lower than their economic and health robustness, like overall resilience results. Only Sweden, the Netherlands, Slovakia, Slovenia, and Estonia experienced their economic decline before the implementation of their measures, while for Switzerland it was simultaneous. As for the time lag between maxima, the lowest point of economic downturn occurred in most cases after the health and stringency index maximums. In addition, except for Finland, Iceland and Greece, all countries began their economic decline around the same time (24.02.2020). In the case of the COVID-19 pandemic, this suggests that economic robustness (the start of economic decline) may be influenced more by regional and global economic shifts than other country parameters such as health system performance and policy implementation. However, the unavailability of weekly data for the GDP ratio-to-trend, and the nature of the indicator itself, may bias these results.

## DISCUSSION

This study is, to the best of our knowledge, the first attempt to operationalize the measurement of a multisystemic expression of resilience by assessing 22 European countries' resilience in three systems - health, economy, and fundamental rights and freedoms. Our approach helps understand how countries are affected and respond to systemic crises by managing different societal objectives between sectors. Furthermore, while the recovery time has been used by various authors as the main resilience indicator [3], our operationalization of robustness, resistance and recovery in addition to an



For peer review only - http://bmjopen.bmj.com/site/about/guidelines.xhtml

overall measure of resilience provide a more granular understanding of how countries were impacted and reacted to the first wave of the COVID-19 pandemic. For example, when considering only the recovery rate, the most resilient country would also be the one with the lowest resistance and the lowest overall resilience in our dataset (Spain).

Based on our results, we established four categories of country based primarily on health and economic resilience during the first wave: (1) high health & high economic resilience: Denmark, Finland, Iceland, Norway, Estonia, Poland, (2) high health & lower economic resilience: Hungary, Slovenia, Slovakia, Greece, Austria, Czech Republic, Germany, (3) higher economic & moderate health resilience: Switzerland, Sweden (4) low health & economic resilience, divided into two subcategories: (4.1) & higher fundamental rights and freedoms resilience: the Netherlands, Belgium, Great Britain, Spain, (4.2) & lower fundamental rights and freedoms resilience: Italy, Portugal, France. Figure 3 provides a graph for Norway, Hungary, Sweden, and Spain to illustrate the four categories. It should be noted that these categories are not prescriptive nor rigid divisions, but rather constantly evolving trends as resilience is an adaptive capacity. Countries are therefore likely to change category as the pandemic evolves.

Countries in category (1) have likely not been affected by the first wave of the Covid-19 pandemic to the same extent as countries with high peak mortality, possibly due to greater geographical isolation, as in the case of Iceland, or a less important caseload before measures were implemented which has been found to be an important factor in the literature [19]. This brings to fore one of the main limitations of our study, namely that different populations have not been similarly exposed to the first wave of COVID-19 and hence limited excess mortality, as in category (1), may not necessarily imply highest health resilience capacity. Nevertheless, all these countries still implemented moderate measures (with the most stringent restrictions for Poland) explained by fear of a new virus and as mimicry of neighboring countries where mortality rates were soaring. Estonia and Iceland, which adopted the least stringent measures also suffered slightly higher economic losses, potentially due to their greater dependence on the tourism industry [20]. Poland, however, is the only country in this group that showed weak fundamental rights and freedoms resilience supporting evidence of continued democratic backsliding during the pandemic [21, 22]. Poland's economic resilience may be attributed to its strong real economic growth rate in recent years.

All countries in category (2), although not having experienced any excess mortality peak, suffered more severe economic downturns resulting in part from the stricter measures implemented by governments. The heavier losses may also be related to greater dependence on tourism, as in Hungary, Greece, Austria, and Slovenia [20], as well as greater levels of economic dependence on foreign trade and exports to other European countries hit by less demand from abroad, as with Germany, the largest European exporter. This points to European countries' vulnerability to global economic shifts including international trade and supply chain disruptions. The pre-pandemic strength of the economy may also be an important determinant that warrants further research. All these countries showed lower fundamental rights and freedoms resilience than category 1. Hungary proved to be the least resilient in terms of measure stringency of this group (and of the comparative analysis), where governments, similar to Poland, used the crisis to introduce tighter restrictions, deteriorating civil and political rights situation as well as media freedom [23].

Countries in category (3), despite higher excess mortality, showed strong economic resilience. For Sweden, the minimal restrictions adopted have allowed businesses to continue operating. However,



For peer review only - http://bmjopen.bmj.com/site/about/guidelines.xhtml

24.06.2022

the economic losses suffered underline the country's economic dependence on exports. Sweden's unique no-lockdown model has indeed demonstrated the highest fundamental rights and freedoms resilience. Switzerland's economic resilience can be attributable to its diversified economy, substantial fiscal space available to support businesses during the crisis as well as highest economic preparedness among OECD countries [24]. Finally, Category 4 countries suffered from both health and economic downturns. Nevertheless, Italy, Portugal, and France proved to be less resilient in terms of fundamental rights and freedoms due in part to weaker crisis preparedness and potentially the balance of power towards the executive for France and Italy.

Our results provide further evidence that the COVID-19 pandemic tested not only the resilience of health systems but also economic and fundamental rights and freedom, but more interestingly that the resilience capacities of one system may depend on and impact the others. It is not surprising that highly affected health systems and the implementation of stringent containment measures notably lockdowns, border closures and travel bans, negatively impact a country's economic development, precipitating a trade decline and financial market volatility. Nevertheless, several countries enforced relatively strict measures while limiting economic consequences due to fewer cases and higher economic resilience. These non-uniform results suggest that the economic capacity to cope and recover from systemic shocks differs more widely between European countries and that countries' economic resilience does not solely relate to national measures, but also to the global and regional economic context.

The correlation between the economic domain with both the health and the fundamental rights and freedoms dimensions reflects both its heavy dependance on and importance to the other sectors' resilience. This correlation supports the need for current economic systems to be structurally transformed in ways that promote their self-organization around recovery to minimize resource requirements to better address global challenges [18]. In contrast, the effect of stringent government responses on health resilience is not as straightforward. Irrespective of their epidemiological situation, all governments adopted varying degrees of measures during the first wave of the COVID-19 pandemic in 2020, related to the novel character of the pandemic and precautionary principles. In addition, Lenton *et al.* found that prolonged stringent measures may not necessarily lead to improved health outcomes and may even reduce health resilience in the long term due to decreased trust in government over time [3].

Finally, our findings demonstrate a strong tendency among the 22 European countries to prioritize their health and economic systems over fundamental rights and freedoms in the first wave, except for Sweden. When considering strictly the health dimension, only nine countries crossed the health robustness threshold and all displayed high recovery capacities during the first wave, unlike other sectors. Among all peaking countries, recovery from excess mortality to pre-shock levels was in fact fastest when the system was less resistant. Whereas we could expect that lower resistance (higher peak) would lead to slower recovery (due to the difficulty of containing cases the more people are infected), a higher peak may result in faster and more effective measures as well as greater societal acceptance of these measures. This may also reflect the rapid depletion of people who were susceptible to the disease when a significant proportion of the population was affected. By the end of August, no government could restore pre-crisis governance operating modes. This suggests that, for fear of the virus and unpreparedness for a pandemic, countries prioritized their health and economic sectors at the expense of fundamental rights and freedoms which are key to the functioning of liberal



For peer review only - http://bmjopen.bmj.com/site/about/guidelines.xhtml

democracies in Europe. However, our indicator for fundamental rights and freedoms resilience (stringency of measures implemented), does not merely reflect the country's inability to absorb the shock and thus may limit the importance of these findings (further discussed below).

There are important limitations in the method used to assess multisystemic resilience. One of the main limitations of assessing resilience across countries is the issue of comparability; countries were not equally exposed to COVID-19, and did not start with the same context, and therefore high resilience results, as in category (1), do not necessarily imply the highest resilience capacity. In other words, our study measures in some ways how resilience capacities were expressed across different systems in relation to how they were exposed to the shock. Why and how it was expressed this way is another question that deserve more scrutiny. This depends both on the capacities of the countries and the capacities and willingness of the governance systems to mobilize these capacities. In addition to this limitation in the interpretation of resilience, several other limitations can be identified regarding the operationalization of the different measurements and the proxy indicators selected.

Besides, there were limitation regarding the method used for measurements. The use of maximum and minimum as time intervals for the calculation of resistance and recovery [3] yields noticeably different results than those obtained with 0 or 5% slope intervals. The latter measurement method enabled us to avoid accounting for the time interval of usual fluctuations and time intervals where countries slowly stabilize near their minimum but continued to decline. This problem was particularly apparent when calculating economic resilience whereby Slovakia and Slovenia experienced a sharp decrease over a one-week interval and recovered immediately thereafter, while other countries had a similar decline during this interval but continued to decrease slowly to their minimum, leading to a higher resistance level than Slovakia and Slovenia when in fact the latter recovered more quickly. To overcome this limitation, resistance and recovery should be measured over a similar time interval.

Robustness has not been often operationalized or integrated into resilience measurement within the literature. We defined a country as robust on the health dimension when able to maintain usual (pre-crisis) health indicators. With this approach, all countries were thus considered robust before the onset of the COVID-19 pandemic. A threshold was chosen based on previous years' maxima to determine which country crossed and in what time frame this threshold, and thus did not maintain its usual health performance. However, this approach involves a certain level of arbitrariness in setting a threshold. An alternative approach would have been to define health robustness as the ability of a country to maintain its usual health services and functionality without change or disruption. We were not able to implement this approach due to broad scope of the study, but the literature has started to address this question [5]. In addition, this approach does not eliminate the difficulty of setting a threshold that separates a normal from a not normal situation. In addition, our method for measuring robustness does not allow for comparisons across countries for one dimension but only comparisons between different sectors within one country. For cross-country comparison, it would be appropriate to use a method determining a probability of passing a certain threshold as proposed by Grafton et al. (2019) [11]. This shortcoming reflects the difficulty of operationalizing the robustness of complex systems, for which further research is needed.

The proxy indicators used to measure health, fundamental rights and freedoms, and economic resilience have shortcomings. Regarding the GDP ratio-to-trend, the OECD stresses that it is more suitable for qualitative purposes to visualize the fluctuations of economic activity. Excess mortality was chosen as it overcomes several issues related to the reporting of COVID-19 related deaths such



For peer review only - http://bmjopen.bmj.com/site/about/guidelines.xhtml

as miscounting from misdiagnosis or under-reporting. However, it does not only include 'direct' COVID-19 mortality as it also includes 'collateral damage' from other health conditions left untreated when the health system is overwhelmed. It also relies on the accuracy of mortality data from the five-year period before the crisis, which is more or less accurate from country to country. The OxCGRT's stringency index to measure the resilience of fundamental rights and freedoms reflects the breaches in civil liberties that are intrinsic to the values of liberal democracies. However, the indicator does not reflect the respect of democratic processes nor the rule of law, essential components to assess broader democratic resilience. Limitations also arise as this indicator is a composite of additive un-weighted indices that abstract away from nuances or any heterogeneity in the country responses. Its reliance on third party sources may also result in measurement bias [25]. Furthermore, unlike health and economic resilience, the stringency of measures does not simply reflect the country's inability to absorb and cope with the shock but also its ability to implement and modify measures quickly. This may explain the varying results between resistance, recovery, and overall resilience, as in the case of Estonia, whose resistance was the lowest but recovery and overall resilience were also the highest. A more accurate estimate of the fundamental rights and freedoms resilience would require a longer study period to track the evolution of measures throughout the pandemic and beyond to examine whether countries leveraged the pandemic to shift towards a more authoritarian system, as it seems to be the case of Poland or Hungary.

We did not compute a unique multisystemic measure of resilience encompassing all sectors as this would have required a normative judgment on whether one system should be considered more important than another. This is fundamentally a political choice. Instead, four different categories of countries were developed which enabled visualization of linkages between sectors' resilience. While stressing the multisystemic nature of resilience, we have limited our analysis to resilience in health, the economy and fundamental rights and freedoms. These systems were chosen because they were more easily discerned and measurable, but systemic effects generated by the COVID-19 crisis have also disrupted social, governance and environmental systems amongst others [14]. Finally, our approach explores only limited aspects of the multiplicity in the ways resilience can be expressed. The use of time series analysis highlights the capacity to cope and recover while overlooking the process of adaptation and transformation that is intrinsically important to the study of resilience [14].

## CONCLUSION

This study serves as a methodological contribution to better understand how the first wave of the COVID-19 pandemic tested not only the capacity of health systems but also the economy and fundamental rights and freedoms. Our findings demonstrated a strong tendency among the 22 European countries to prioritize their health and economic systems over fundamental rights and freedoms during the first wave of the COVID-19 pandemic that hit Europe in spring 2020. Overall, our assessment of multisystemic resilience to gauge expressed capacities of several European countries to tackle the first wave of the COVID-19 pandemic provides an important step for understanding the capacities of countries to cope, adapt and transform can be expressed during a shock.

By exploring the relationships between robustness, resistance, and recovery, our study draws our attention the dynamics of resilience in complex systems. With limited pandemic preparedness capacities, it appears clearly that no country was robust in all three dimensions. A lack of capacity to rapidly control the pandemic was thus compensated by strong restrictions on individual rights and freedoms. Economic resilience was also found to be significantly linked to health outcomes as well as



For peer review only - http://bmjopen.bmj.com/site/about/guidelines.xhtml

For peer review only

24.06.2022

the stringency of measures, suggesting the dependence on, and importance to, the other sectors' resilience. Furthermore, recovery varied widely both across the three dimensions and across countries. Overall, this suggests that resilience by interventions implies trade-offs between systems with different objectives.

Given the fact that the COVID-19 pandemic has not been a single event but recurring waves of different magnitude, we believe that our approach should be applied to study resilience to the different waves of the pandemic. This will lead to an understanding of whether and how countries adapt their priorities and strategies over time. Based on our approach to measure resilience, a related key issue will be to understand whether, over the duration of the COVID-19 pandemic, it makes more sense to escalate and deescalate quickly or whether it is more relevant to keep similar levels of measures when it comes to an event that is likely to have recurring patterns and last for some time. While more research is needed on multisystemic resilience, our paper ultimately sheds light on the relevance of the study of resilience to improve governance capacities to prevent, react to, and recover from the current and future systemic shocks.

For peer review only

## Ethics Statement

This study is based on online search of publicly available data. No patient, nor the public were involved in the design of the study. No ethical approval was sought as per our university requirements.

## Contributors:

Didier Wernli, Mia Clausin and Alicia Rieckhoff conceptualized the multisystemic measurement approach to resilience. Mia Clausin and Alicia Rieckhoff analysed the data and developed the successive drafts of the paper. Mia Clausin and Alicia Rieckhoff designed the figures and tables. All co-authors contributed content and comments to the paper. Didier Wernli and Nicolas Levrat are both principal investigators within the COVID-19 systemic crisis project.

## Competing Interests:

None declared

## Funding:

This research was funded by the Swiss National Science Foundation (SNSF), within the scope of the COVID-19 systemic crisis project (Grant 31CA30_196396, https://data.snf.ch/covid-19/snsf/196396)

## Data Sharing Statement

Patient consent for publication is not applicable.

# REFERENCE LIST


1. Linkov, I., et al., *Changing the resilience paradigm.* Nature Climate Change, 2014. **4**(6): p. 407-409.
2. Wernli, D., et al., *Governance in the age of complexity: building resilience to COVID-19 and future pandemics*. 2021, Geneva Science Policy Interface, University of Geneva, Switzerland.
3. Lenton, T.M., C.A. Boulton, and M. Scheffer, *Resilience of countries to COVID-19 correlated with trust.* Scientific Reports, 2022. **12**(1): p. 75.
4. Haldane, V., et al., *Health systems resilience in managing the COVID-19 pandemic: lessons from 28 countries.* Nature Medicine, 2021.
5. Arsenault, C., et al., *COVID-19 and resilience of healthcare systems in ten countries.* Nature Medicine, 2022.




For peer review only - http://bmjopen.bmj.com/site/about/guidelines.xhtml

24.06.2022

6. Saulnier, D.D., et al., *A health systems resilience research agenda: moving from concept to practice.* BMJ Global Health, 2021. **6**(8): p. e006779.
7. Helbing, D., *Globally networked risks and how to respond.* Nature, 2013. **497**(7447): p. 51-59.
8. Ungar, M., *Multisystemic resilience: Adaptation and transformation in contexts of change*. 2021: Oxford University Press, USA.
9. Folke, C., et al., *Resilience thinking: integrating resilience, adaptability and transformability.* Ecol. Soc., 2010. **15**: p. 20.
10. Pimm, S.L., et al., *Measuring resilience is essential to understand it.* Nature Sustainability, 2019. **2**(10): p. 895-897.
11. Grafton, R.Q., et al., *Realizing resilience for decision-making.* Nature Sustainability, 2019. **2**(10): p. 907-913.
12. Galaitsi, S.E., et al., *The Need to Reconcile Concepts that Characterize Systems Facing Threats.* Risk Analysis, 2021. **41**(1): p. 3-15.
13. Schiller, C., et al., *Just How Resilient are OECD and EU Countries? Sustainable Governance in the Context of the COVID-19 Crisis*. 2021, Bertelsmann Stiftung.
14. Wernli, D., et al., *Building a multisystemic understanding of societal resilience to the COVID-19 pandemic.* BMJ Global Health, 2021. **6**(7): p. e006794.
15. *Human Mortality Database [Internet]*. [cited 2021 16 August]; Available from: https://www.mortality.org/.
16. *COVID-19 Government Response Tracker [Internet]*. [cited 2021 16 August]; Available from: https://www.bsg.ox.ac.uk/research/research-projects/covid-19-government-response-tracker.
17. *Main Economic Indicators Publication [Internet]*. [cited 2021 16 August]; Available from: https://stats.oecd.org/Index.aspx?DataSetCode=MEI.
18. Hynes, W., et al., *Systemic resilience in economics.* Nature Physics, 2022. **18**(4): p. 381-384.
19. Haug, N., et al., *Ranking the effectiveness of worldwide COVID-19 government interventions.* Nature Human Behaviour, 2020.
20. OECD, *OECD Tourism Trends and Policies 2020*. 2020.
21. Lewkowicz, J., M. Woźniak, and M. Wrzesiński, *COVID-19 and erosion of democracy.* Economic modelling, 2022. **106**: p. 105682-105682.
22. International Institute for Democracy and Electoral Assistance, *The State of Democracy in Europe 2021 - Overcoming the impact of the pandemic*. 2021.
23. Bene, M. and Z. Boda, *Hungary: Crisis as Usual—Populist Governance and the Pandemic*, in *Populism and the Politicization of the COVID-19 Crisis in Europe*, G. Bobba and N. Hubé, Editors. 2021, Springer International Publishing: Cham. p. 87-100.
24. OECD, *OECD Economic Surveys: Switzerland 2022*. 2022.
25. Hale, T., et al., *Government responses and COVID-19 deaths: Global evidence across multiple pandemic waves.* PLOS ONE, 2021. **16**(7): p. e0253116.



For peer review only - http://bmjopen.bmj.com/site/about/guidelines.xhtml

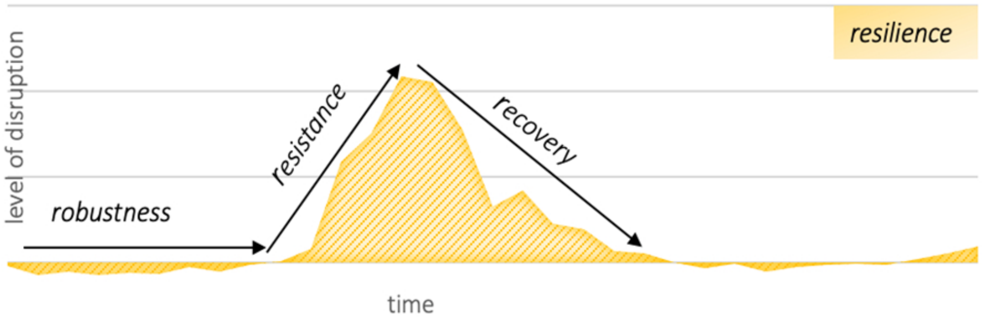


Conceptual framework of resilience and its sub-components: robustness, resistance, and recovery inspired by Grafton et al. [11]

402x139mm (57 x 57 DPI)

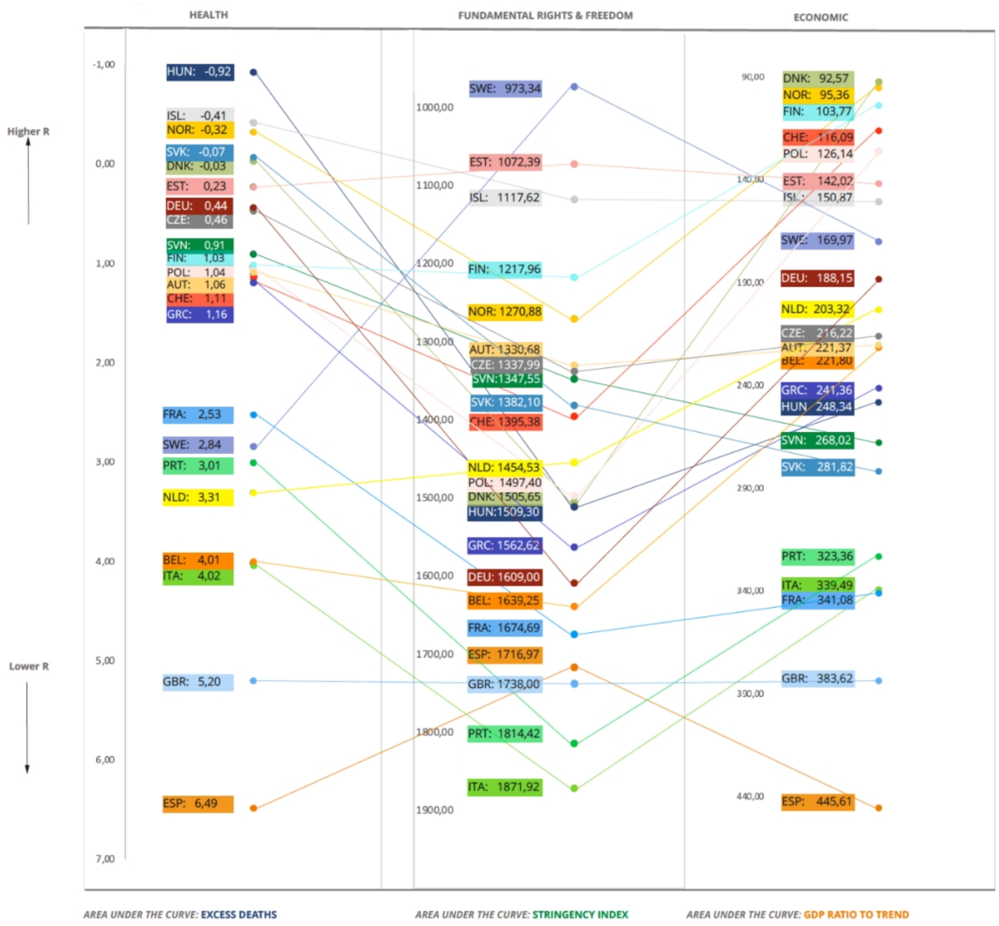


Overall comparison of resilience (area under the curve) of the health, fundamental rights and freedoms, and economic domains.

402x383mm (87 x 87 DPI)

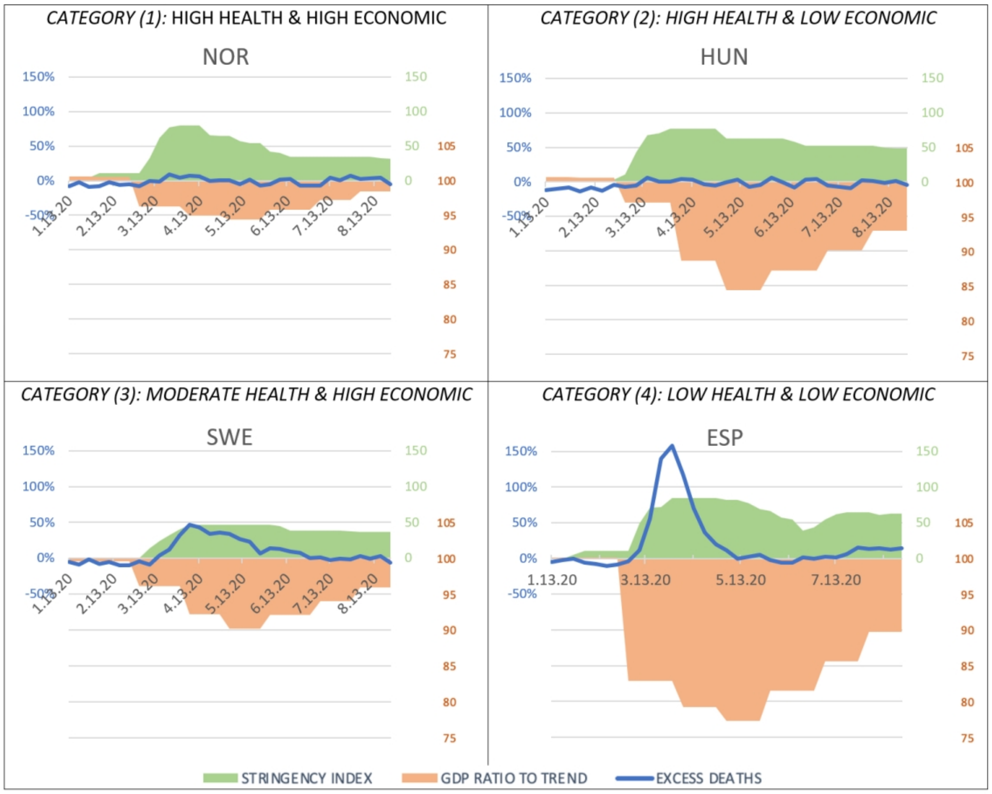


Figure 3 Time series analysis of selected countries representing the 4 categories and combining resilience measurements of the health, fundamental rights and freedoms, and economic domains.

681x551mm (57 x 57 DPI)

# SUPPLEMENTARY MATERIAL

*S1 Time series analysis for 22 countries combining measurements about the health, fundamental rights and freedoms, and economic dimensions of resilience*

For peer review only

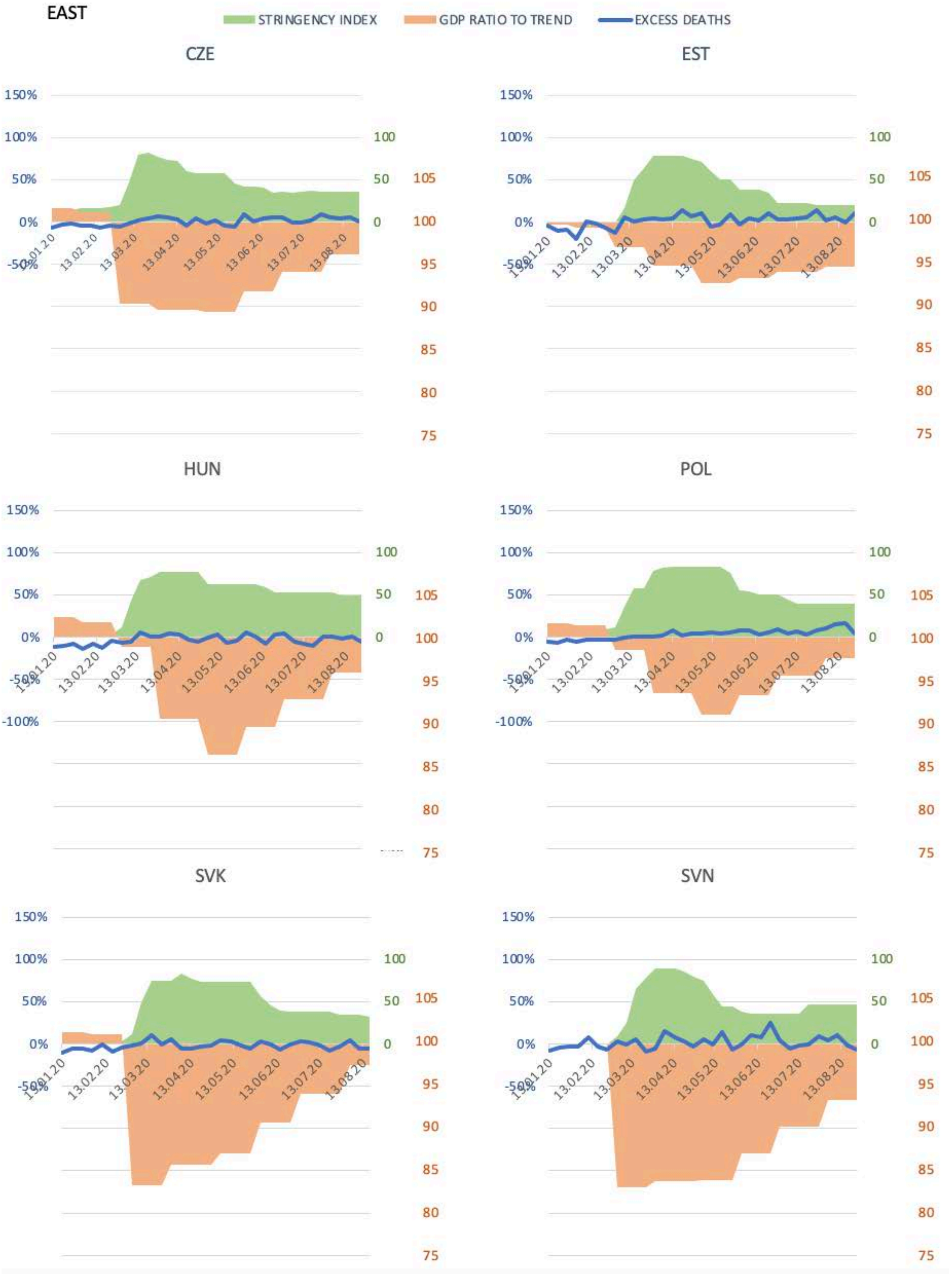
EAST
STRINGENCY INDEX
GDP RATIO TO TREND
EXCESS DEATHS
CZE
EST
HUN
POL
SVK
SVN

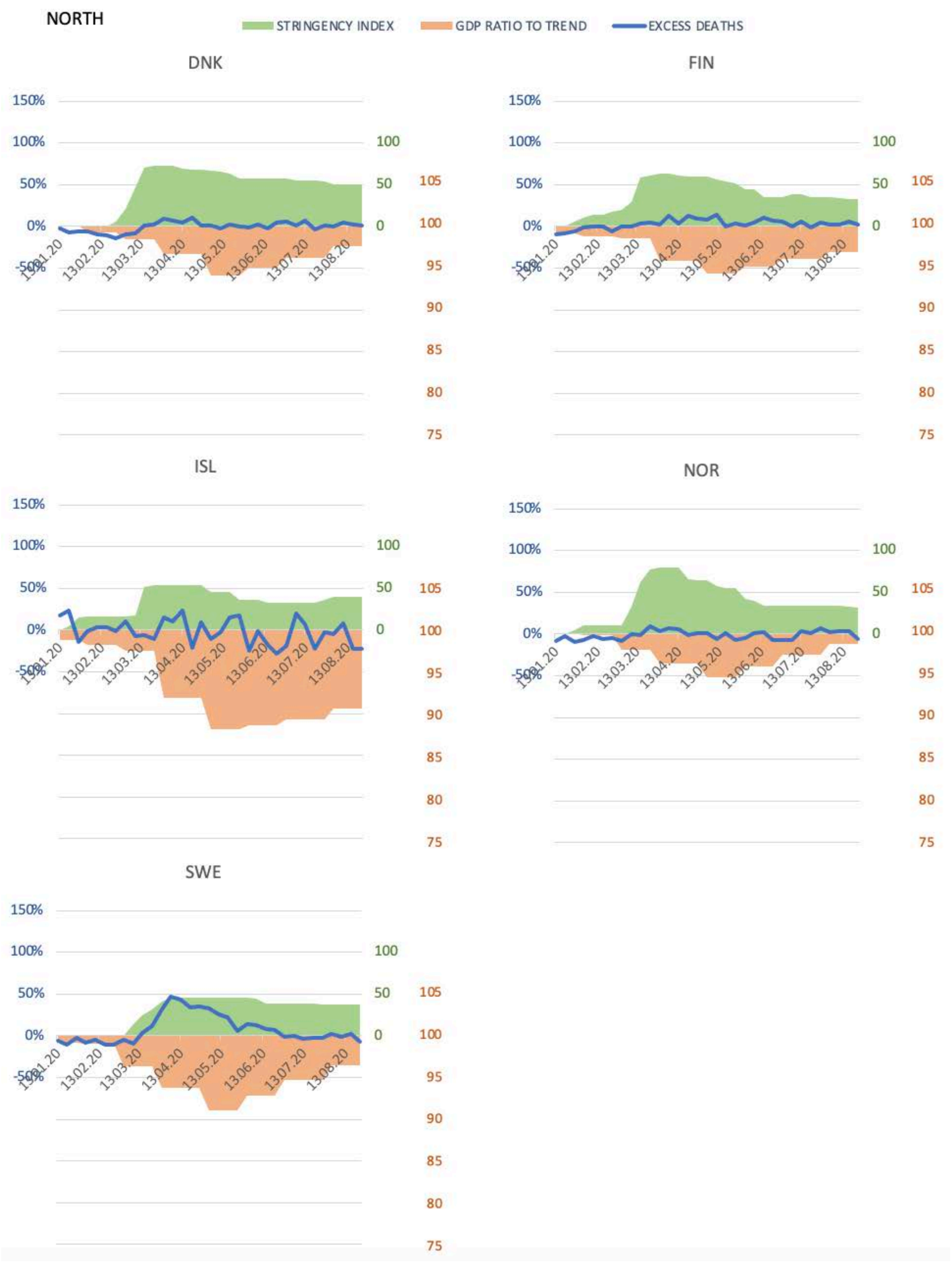
NORTH
STRINGENCY INDEX
GDP RATIO TO TREND
EXCESS DEATHS
DNK
FIN
ISL
NOR
SWE
150%
100%
50%
0%
-50%
100
50
0
105
100
95
90
85
80
75
13.01.20
13.02.20
13.03.20
13.04.20
13.05.20
13.06.20
13.07.20
13.08.20

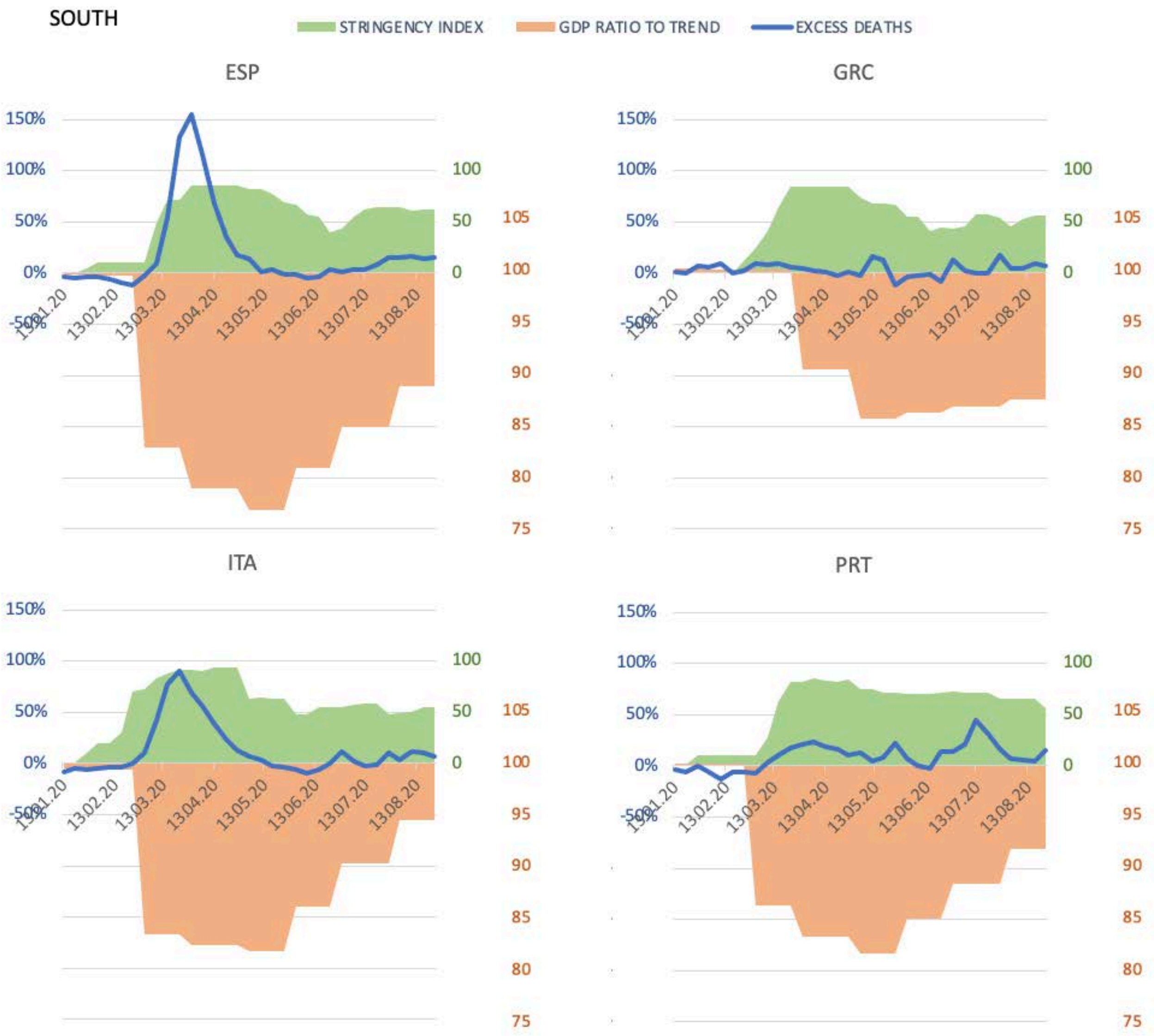
SOUTH
STRINGENCY INDEX
GDP RATIO TO TREND
EXCESS DEATHS
ESP
GRC
ITA
PRT
150%
100%
50%
0%
-50%
100
50
0
105
100
95
90
85
80
75
13.01.20
13.02.20
13.03.20
13.04.20
13.05.20
13.06.20
13.07.20
13.08.20

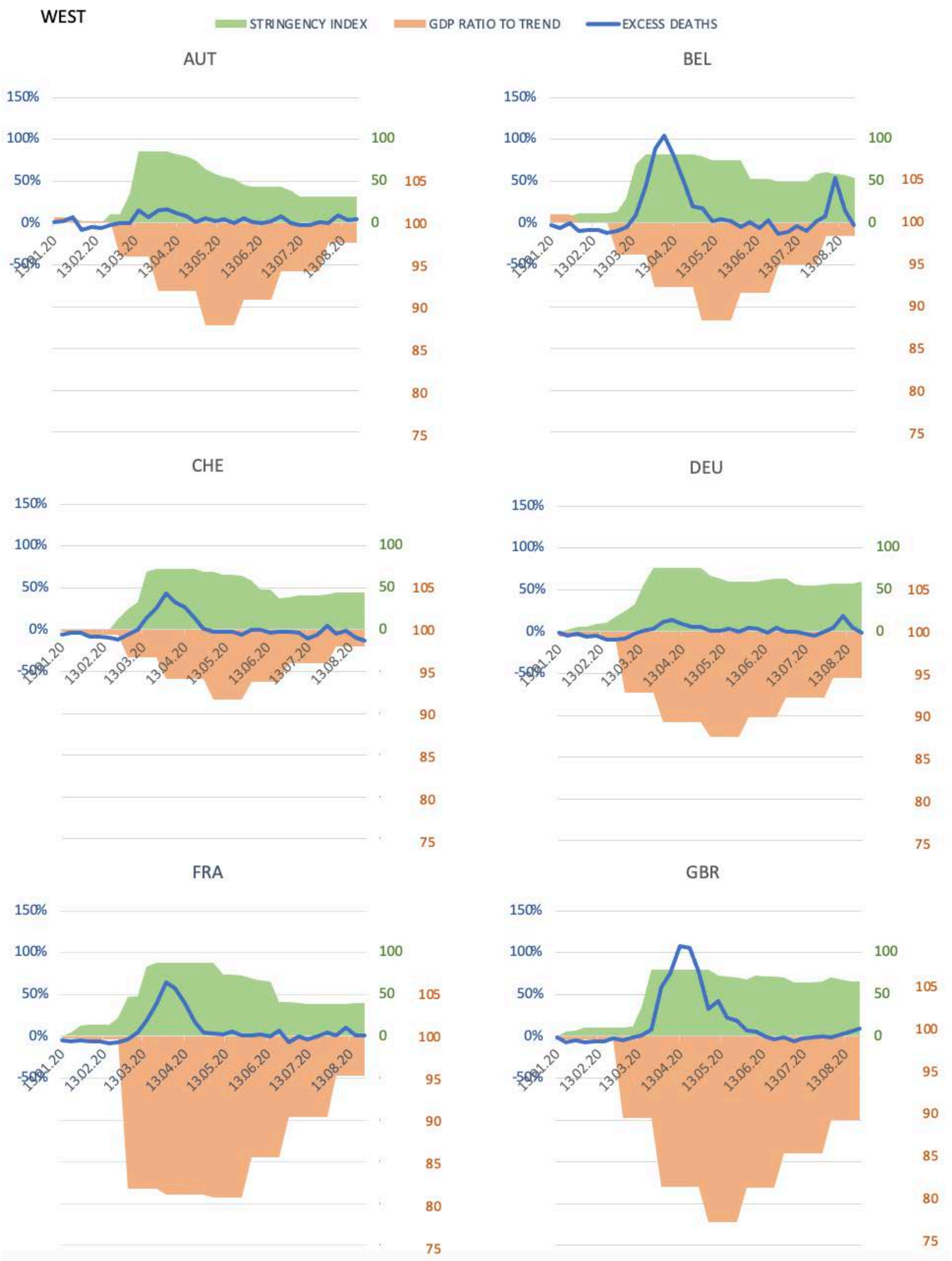
WEST
STRINGENCY INDEX
GDP RATIO TO TREND
EXCESS DEATHS
AUT
BEL
CHE
DEU
FRA
GBR
150%
100%
50%
0%
-50%
13.01.20
13.02.20
13.03.20
13.04.20
13.05.20
13.06.20
13.07.20
13.08.20
100
50
0
105
100
95
90
85
80
75

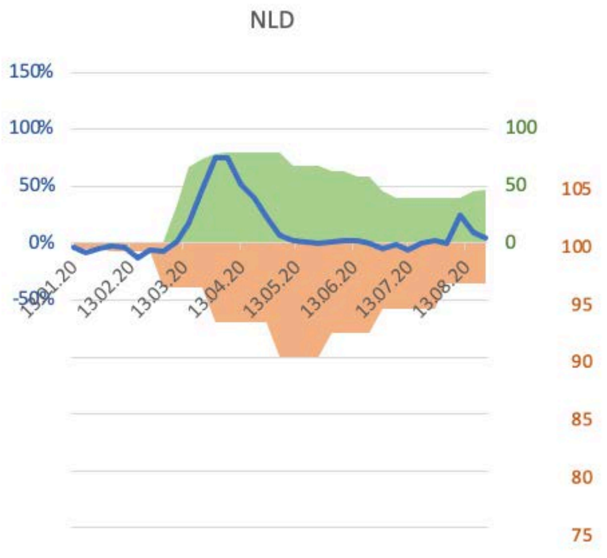
NLD
150%
100%
50%
0%
-50%
100
50
0
105
100
95
90
85
80
75
13.01.20
13.02.20
13.03.20
13.04.20
13.05.20
13.06.20
13.07.20
13.08.20

BMJ Open

# Measuring multisystemic resilience to shocks: health, fundamental rights and freedoms, and economic resilience during the COVID-19's first wave in Europe.



| | |
|---|---|
| Journal: | *BMJ Open* |
| Manuscript ID | bmjopen-2022-065445.R1 |
| Article Type: | Original research |
| Date Submitted by the Author: | 15-Mar-2023 |





| | |
|---|---|
| Complete List of Authors: | Clausin, Mia; University of Geneva, Global Studies Institute<br>Rieckhoff, Alicia; University of Geneva, Global STudies Institute<br>Tediosi, Fabrizio; Swiss Tropical and Public Health Institute, Household Economics & Health Systems Research Unit<br>Morel, Chantal; University of Geneva, Global Studies Institute<br>Kaspiarovich, Yuliya; University of Geneva, Global Studies Institute<br>Levrat, Nicolas; University of Geneva, Global Studies Institute; Faculty of Law, University of Geneva<br>Wernli, Didier; University of Geneva |





| | |
|---|---|
| <b>Primary Subject Heading</b>: | Health policy |
| Secondary Subject Heading: | Global health, Infectious diseases |
| Keywords: | COVID-19, PUBLIC HEALTH, INFECTIOUS DISEASES |



SCHOLARONE™
Manuscripts

*I, the Submitting Author has the right to grant and does grant on behalf of all authors of the Work (as defined in the below author licence), an exclusive licence and/or a non-exclusive licence for contributions from authors who are: i) UK Crown employees; ii) where BMJ has agreed a CC-BY licence shall apply, and/or iii) in accordance with the terms applicable for US Federal Government officers or employees acting as part of their official duties; on a worldwide, perpetual, irrevocable, royalty-free basis to BMJ Publishing Group Ltd ("BMJ") its licensees and where the relevant Journal is co-owned by BMJ to the co-owners of the Journal, to publish the Work in this journal and any other BMJ products and to exploit all rights, as set out in our licence.*

*The Submitting Author accepts and understands that any supply made under these terms is made by BMJ to the Submitting Author unless you are acting as an employee on behalf of your employer or a postgraduate student of an affiliated institution which is paying any applicable article publishing charge ("APC") for Open Access articles. Where the Submitting Author wishes to make the Work available on an Open Access basis (and intends to pay the relevant APC), the terms of reuse of such Open Access shall be governed by a Creative Commons licence – details of these licences and which Creative Commons licence will apply to this Work are set out in our licence referred to above.*

*Other than as permitted in any relevant BMJ Author's Self Archiving Policies, I confirm this Work has not been accepted for publication elsewhere, is not being considered for publication elsewhere and does not duplicate material already published. I confirm all authors consent to publication of this Work and authorise the granting of this licence.*

For peer review only

14.03.2023

# Measuring multisystemic resilience to shocks: health, fundamental rights and freedoms, and economic resilience during the COVID-19's first wave in Europe.

Mia Clausin[1*], Alicia Rieckhoff[1*], Fabrizio Tediosi[2-3], Chantal Morel[1], Yuliya Kaspiarovich[1], Nicolas Levrat[1], Didier Wernli[1]

[1] Geneva Transformative Governance Lab, Global Studies Institute, University of Geneva, Switzerland
[2] Swiss Tropical and Public Health Institute, Basel, Switzerland
[3] University of Basel, Basel, Switzerland

* These authors contributed equally.

Keywords: resilience, robustness, recovery multisystemic, health systems, governance, complexity, systemic risks, systemic crisis, COVID-19

Corresponding author: Didier.Wernli@unige.ch

Word count: 6769 in the main text. 2 tables, 3 figures, 7 supplementary materials. 28 references.

Abstract:
**Objectives:** While resilience research on COVID-19 has primarily focused on health system resilience, the purpose of this paper is to measure 'multisystemic' resilience (in the health, economic, and fundamental rights and freedoms dimensions) of 22 European countries and resilience dynamics during the first wave of the COVID-19 pandemic by assessing different components of resilience.
**Methods/Design:** Multisystemic resilience was assessed through time-series analyses in 22 European countries of the effects generated by the first wave of the COVID-19 pandemic in three sectors: health, economy, and fundamental rights and freedoms. An overall (cumulative) resilience was estimated, as well as three of its sub-components: robustness, resistance, and recovery.
**Participants:** This study is based on an online search of publicly available data. No patient, nor the public were involved in the design of the study.
**Results:** While only 7 countries exhibited an excess mortality peak that was an outlier compared to 2015-2019 during the first wave of the COVID-19 pandemic, all countries were impacted economically and implemented various restrictions levels. Four groups of countries are identified: 1) high health & high economic resilience, (2) high health & lower economic resilience (3) higher economic & moderate health resilience: Switzerland, Sweden (4) low health & economic resilience.
**Conclusions:** This paper provides a novel approach for the systematic assessment of multisystemic resilience to gauge the strengths and shortcomings of countries' capacities to tackle systemic crises.

Strengths and limitations of this study:

- First study to operationalize the resilience of 22 European countries to the first wave of the COVID-19 pandemic in three sectors – health, economy, and fundamental rights and freedoms.
- Three resilience components – robustness, resistance, and recovery – in addition to an overall measure of resilience have been measured, and different measurements methods have been tested.
- The primary limitation when assessing resilience lies in country comparability (countries were not exposed to COVID-19 in the same way) and the interpretation of resilience.



For peer review only - http://bmjopen.bmj.com/site/about/guidelines.xhtml

14.03.2023

- Selected indicators only partially capture the resilience, overlooking other factors influencing countries' ability to absorb and cope with the shock.
- While measurement of resilience remains in need of further research, the various measurement methods tested showed significant variations in results.

## Introduction

The COVID-19 pandemic has tested the resilience of countries to deal with a macro-shock affecting health, economic, social, environmental, and governance systems worldwide. In an early 21st century increasingly characterized by uncertainty, complexity, and systemic disruptions, resilience thinking has gained traction in various fields including disaster management, the environment, and more recently global health [1, 2]. While research on resilience to COVID-19 primarily focuses on health systems, [3-5], several studies have called for the operationalization of resilience to go beyond the functions of the health system in response to shocks [6]. Considering resilience as a multi-objective societal problem, the concepts of 'systemic resilience' [7] and 'multisystemic resilience' [8] have emerged to better understand the impact of shocks across highly interconnected systems.

There are currently different definitions of resilience across fields ranging from the capacity of a system to come back to its initial state to the capacity "*to absorb disturbance and reorganise while undergoing change so as to still retain essentially the same function, structure, identity, and feedbacks*" [9]. Resilience thinking therefore draws attention to both the vulnerability of systems and the capacity to respond. However, with no universally accepted definition bridging sectoral divides and limited methodological development across disciplines, the measurement of resilience remains underdeveloped [10]. Stemming from recent works, resilience as a property of a complex system can be measured according to three characteristics: robustness, resistance, and recovery [11, 12]. The main idea is that resilience can be expressed in different ways according to the stage of an endogenous disruption or exogenous shock.

While the policy literature has also sought to operationalize measurement of multisystemic resilience [13], this paper aims to operationalize multisystemic resilience and provide a practical application of how to measure resilience and its dynamics over the first wave of the COVID-19 pandemic by assessing three components of resilience – robustness, resistance, and recovery [11]. This is a cross-country comparative study depicting the first wave of the COVID-19 pandemic in 22 European countries through multi-component time-series analyses, measuring health resilience, economic resilience and resilience of fundamental rights and freedoms (social and environmental resilience were not covered in this study due to a lack of data). While the pandemic is not over, this study serves as a methodological contribution to better understand how different systems are affected by the COVID-19 crisis and impact each other, to mitigate undesirable knock-on effects in the long-run. Understanding resilience as the ability of societies to maintain their core governance functions while minimising the undesirable societal effects can serve as the foundation for an integrated approach to building societal resilience to future pandemics [14].



For peer review only - http://bmjopen.bmj.com/site/about/guidelines.xhtml

14.03.2023

# Methods

## Public and Patient Involvement

This study is based on online search of publicly available webpages and documents. No patient, nor the public were involved in the design of the study. No ethical approval was sought as per our university requirements.

## Study design for time-series analyses

The proxy measures for resilience of the health system, fundamental rights and freedoms, and economic system were selected using two criteria: first, the availability of weekly or monthly indicators which would allow for a detailed time-series analysis; second, their relevance to capture the type of resilience at hand. Table 1 outlines the definitions we used for resilience and its 3 components across the different sectors. Data was collected between week 3 when no measures were implemented, to week 35 when COVID-19 death near 0 for all European countries (13.01.2020-31.08.2020). Twenty-two European countries were selected according to the availability of data across the selected dimensions of resilience.

The indicator selected for measuring health system resilience was weekly excess mortality, from the Human Mortality Database [15]. A death is recorded by week of occurrence, apart from the UK, where death by week of registration is used. Excess mortality was selected as it helps overcome several issues related to the reporting of COVID-19 related deaths such as miscounting from misdiagnosis or under-reporting. It also includes 'collateral damage' from other health conditions left untreated if the health system is overwhelmed [5]. Excess mortality was defined as the number of deaths registered in excess of the five-year average from 2015 to 2019. The calculations for Germany and Greece were based on the four-year average (2016-2019) due to unavailable data. A weekly P-score, which was chosen as it allows for robust country comparisons, was calculated as the percentage difference between the reported and projected number of deaths. Health resilience was interpreted as the inverse of excess mortality. The lower the excess mortality, the larger health resilience.

The indicator selected for the resilience of fundamental rights and freedoms was the Oxford Coronavirus Government Response Tracker's (OxCGRT) stringency index [16]. The stringency index records the strictness of "lockdown style" policies that primarily restrict people's behaviour. The index is a composite measure of categorical variables based on nine indicators: school closing, workplace closing, cancelling of public events, restrictions on the size of gatherings, closing of public transport, stay-at-home requirements, restrictions on internal movement, restrictions on international travel and public information campaigns. It therefore provides a proxy indicator to measure restrictions in fundamental rights and freedoms, considering that the latter (e.g., freedom of movement; right to assembly and demonstration) are intrinsic to the functioning of liberal democracies. While the indicator is a daily value between 1 and 100 (100 = strictest), a weekly average was derived for this study. Resilience of fundamental rights and freedoms was measured as the inverse of the stringency index. This means that the more stringent was the response, the less resilient were fundamental rights and freedoms.

The economic system resilience indicator selected was the OECD's short-term main economic indicator: Gross Domestic Product (GDP) ratio to trend [17]. This indicator is a monthly derived indicator where GDP, which is a monetary measure of the market value of all goods and services produced and sold in a specific time period, is divided by the long-term GDP trend to give a ratio-to-



For peer review only - http://bmjopen.bmj.com/site/about/guidelines.xhtml

14.03.2023

trend. It is notably useful to visualise economic cycles. If the GDP ratio to trend = 100, GDP is equal to long-term GDP trends. If the GDP ratio to trend is higher than 100, GDP is higher than long-term GDP trends which signified economic expansion. If the GDP ratio to trend is below 100, the GDP is lower than the long-term GDP trend signalling that the 'growth' (if any) is less than trend. GDP ratio to trend data were compared to quarterly GDP, i.e., the percentage change from the same quarter in previous years, to check for its accuracy in representing the magnitude of the shock. Economic resilience to the shock was interpreted as the inverse of the decline of the GDP ratio.

*Table 1 Resilience to COVID-19 and its applications to health, fundamental rights and freedoms, and the economy based on resilience and three core subcomponents (inspired by Grafton et al. [11])*

| Definitions | Health system | Fundamental rights and freedoms | Economic system |
|---|---|---|---|
| **Resilience** is the capacity of a system to withstand, absorb, and recover from disturbance. | Capacity to meet significant increases in demand for both public health and healthcare services and adapt to long-standing changes. | Capacity to preserve and protect individual rights and freedoms while maintaining the respect for the rule of law. | Capacity to limit the magnitude of economic losses, recovery quickly, and forge new developmental paths for prosperity. |
| ***Robustness*** is capacity of a system to maintain its identity/performance and not cross an undesirable threshold following the adverse event. | Capacity to maintain health services and population health within the range of normal variation. | Capacity to maintain respect for individual rights and freedoms within the range of normal variation. | Capacity to maintain economic indicators performance within the range of normal variation. |
| ***Resistance*** is the capacity to absorb disruption with minimal damage to system functionality. | Capacity to slow down COVID-19 transmission and excess mortality. | Capacity to minimize an escalation of stringent measures violating fundamental rights and freedoms. | Capacity to minimize economic disruption and impact of the shock. |
| ***Recovery*** is capacity to 'bounce-back' following disruption. | Capacity to recover to a scenario of pre-pandemic excess mortality rates. | Capacity to end emergency measures that impact fundamental rights and freedoms. | Capacity to recover to a scenario with pre-pandemic GDP trends and avoid a long-term recession. |

## Measuring resilience, robustness, resistance, and recovery

The conceptual framework to measure resilience and its sub-components is visualised in Figure 1. Table 2 outlines the criteria used to measure health, fundamental rights and freedoms, and economic resilience, robustness, resistance, and recovery. Overall resilience in health, fundamental rights and freedoms and the economy was estimated using the inverse of the area under the curve during the study period. It was calculated by adding the area of data points until the horizontal axis (when x=0 for excess mortality and stringency index, or x=100 for GDP ratio to trend) and subtracting the area of data points beyond the horizontal axis (when x<0 for excess mortality and x>100 for GDP ratio to trend). Data points used to calculate the area under the GDP ratio to trend curve were computed as the point difference to trend (if x=97, then 100-97=3 was coded). Results were then used to conduct a comparative analysis across sectors and identify general trends and potential trade-offs between them. This allowed us to analyse the resilience level relative to other sectors' results and minimise bias caused by different time exposure to the virus from one country population to another. The greater the difference between two sectors, the more resilient it was considered in that domain.



For peer review only - http://bmjopen.bmj.com/site/about/guidelines.xhtml

14.03.2023

For peer review only



For peer review only - http://bmjopen.bmj.com/site/about/guidelines.xhtml

14.03.2023

***Table 2*** *Measurements of resilience, robustness, resistance, and recovery across the health – fundamental rights and freedoms, and economic spectrum*

| | **Resilience** | **Robustness** | **Resistance rate** | **Recovery rate** |
|---|---|---|---|---|
| Health system | Inverse of the area under the excess mortality curve using the trapezoid rule between 13.01.20 – 31.08.20 | The value for the period considered is not an outlier compared to 2015-2019 | Inverse of the slope of linear regression between value closest to 0 and maximum | Slope of linear regression between maximum to first value closest to zero |
| Fundamental rights and freedoms | Inverse of the area under the stringency index curve using the trapezoid rule between 13.01.20 – 31.08.20 | The value for the period considered is an outlier compared to 2015-2019 | Inverse of the slope of linear regression between value closest to 0 and minimum | Slope of linear regression between maximum and minimum value (within a decreasing trend) |
| Economic system | Inverse of the area above GDP ratio-to-trend curve using the trapezoid rule between 13.01.20 – 31.08.20 | The value for the period considered is an outlier compared to 2015-2019 | Inverse of the slope of linear regression between value closest to 0 and minimum | Slope of linear regression between minimum and maximum value within an increasing trend |

Robustness primarily means that ''a system continues to work at the same level. For example, the definition of health robustness is the capacity to maintain all health services [5]. In case of increased demand (overload), this means that the system will be able to provide the same level of services. Finding a precise threshold of disruption is challenging as it will depends on the sector affected and may involve arbitrary considerations. A suitable approach to assess robustness is to evaluate whether the system has continued to demonstrate the performance in line with previous years. In this study, we used data from previous years and looked whether the first wave of the COVID-19 pandemic was an outlier regarding our selected health and economic resilience indicators (the detail of the methodology is available in supplementary material 1-4). Finally, regarding fundamental rights and freedoms, the situation is different as the Oxford stringency index was introduced with the pandemic and there is not point of comparison with the period preceding the pandemic. In this case, we computed a z-score for each country to understand how they did regarding the average of all 22 countries.

Resistance and recovery were estimated using the slope of linear regressions leading to the peak (resistance) and away from the peak (recovery). We used F-test to assess the statistical significance of the calculated slopes. Regarding resistance, we used the interval of data from a minimum (the selected start points was the negative or positive value closest to 0 ) to the maximum or peak during the period considered from week 3 to 35) [3]. For countries that experienced two or more 'peaks', we selected the first peak (as the goal of the paper is to assess resilience at the onset of the pandemic). Overall, this means that different countries may have different starting and ending points. A higher slope value (in %) represents a lower level of resistance. For economic resistance, the slope was calculated from the value closest to 100 to the lowest value. Recovery was measured using the slope of the linear regression over the data interval between the maximum value (minimal value for the economic indicator) and the minimal subsequent value. A higher slope value (in %) represents a faster level of recovery. For all calculations, we defined a continuous trend as the presence of no more than one subsequent value that does not follow the upward or downward trend. The rationale for this strategy was to find replicable strategy and focus on the process of resistance and recovery.

We calculated Pearson correlation coefficients between the different sectors as well as between resilience's components to evaluate potential association trends. While we were able to evaluate overall resilience for each country, results about resistance and recovery that were not statistically significant were removed from those calculation. Finally, to further assess multisystemic resilience, we compared the overall resilience value for each section. In addition, we used the z-score to derive



For peer review only - http://bmjopen.bmj.com/site/about/guidelines.xhtml

two main metrics which is first the difference between excess mortality and stringency that gives an indication of the adequation between the magnitude of the problem and response. The second score is the difference between the state of liberal democracy and the stringency index which gives an idea about the departure from the traditional way of governing (the methodology is described in supplementary material 5).

# Results

We present the results by systems and then from a multisystemic perspective. Supplementary material 6 synthesizes the results of the measurements of the three components of resilience (i.e., robustness, resistance, and recovery) for health, fundamental rights and freedoms, and economic dimensions.

## Health resilience

During the first wave in Europe, up to 24 August 2020, 7 countries – Spain (max/peak = 158%), Great Britain (108%), Belgium (105%), Italy (85%), the Netherlands (75%), France (65%), Sweden (47%), – had exhibited at an excess mortality peak That was an outlier compared to 2015-2019. The remaining countries (maximum values from 6% to 45%) were not outliers compared to previous years (2015-2019) and can be considered as robust during the first wave. However, Switzerland (45%), and Portugal (44%) experienced peaks above 40%. Several countries (Austria, Germany, Finland, Iceland, Portugal, Estonia, Greece and Slovenia) reported at least two non-consecutive values above 10% of excess mortality). Among them only Portugal had two statistically significant peaks. Czechia, Hungary, and Norway did not report excess mortality above 10% during the full period. Eight of the nine countries with a mortality peak – Spain (649), Great Britain (520), Italy (402), Belgium (401), the Netherlands (331), Portugal (301), Sweden (284), and France (253) – also had the highest cumulative excess mortality over the study period (area under the curve), suggesting a low overall health resilience to the first wave of the COVID-19 pandemic in spring 2020. Switzerland scored a much lower cumulative excess mortality (111). Five countries registered negative cumulative excess mortality – Hungary (-92), Iceland (-41), Norway (-32), and Slovakia (-7). The cumulative resilience average for the 22 countries was 168.64.

For the 10 countries for which significant values of both resistance and recovery could be calculated, the peak of excess mortality was reached in 4,8 weeks on average while the average recovery time was 7.7. Health resistance and recovery were negatively correlated: the lower the health resistance (higher peak), the faster the recovery ($r(10) = .81$, $p < .001$). Spain showed the lowest resistance (45.28%) with excess mortality rates increasing by 6.0-fold in the week of the 9th of March and then almost tripling to 158.25% within two weeks. Yet, the country also showed the highest recovery rate (-26.23%). Both low level of resistance ($r(10) = .87$, $p < .001$), and high level of recovery ($r(10) = .81$, $p < .005$), were associated with overall cumulative excess mortality. Spain, with the lowest resistance and highest recovery, also had the lowest cumulative health resilience (649). Yet, low resistance does not necessarily imply low overall resilience. Switzerland exhibited a mortality peak and rather low resistance of 14.5% but with a cumulative excess mortality of only 111, similar to other non-peaking countries like Austria (106) or Greece (116). This can be explained by the country's high recovery rate (-9.60%). With a resistance close to Switzerland's, Sweden (13.8%) had a lower recovery rate (-3.74%) as well as significantly cumulative excess mortality (284), compatible with the implementation of less stringent government intervention. This further illustrates the importance of the recovery capacity on



For peer review only - http://bmjopen.bmj.com/site/about/guidelines.xhtml

14.03.2023

overall health resilience. Most excess mortality data were positively skewed (mean>median), testifying that high excess mortality occurred earlier in the period (usually March-April), rather than the summer where in most countries excess mortality declined apart from some countries which experienced a second higher peak.

## Resilience of fundamental rights and freedoms

All 22 countries implemented varying levels of restrictions and during the first wave. Given the level the unprecedented level of restrictions implemented in all countries, none can be considered as robust in terms of fundamental rights and freedoms. This is corroborated by further data which showed a further decline in democracy compared to previous years. [18, 19] The highest values of the stringency index were recorded by Italy (93.5), Slovenia (89.8) and France (88), while the lowest by Sweden (46.3), Iceland (53.5) and Finland (63). As of 24 August 2020, no country had returned to its pre-pandemic situation. Italy (1871.9), Portugal (1814.4), Great Britain (1738), and Spain (1717) exhibited the highest cumulative stringency over the study period, suggesting a low resilience of fundamental rights and freedoms, while Sweden (973.3), Estonia (1072.4), Iceland (117.6), and Finland (1218) exhibited the lowest cumulative stringency, indicating higher fundamental rights and freedoms resilience. The average cumulative fundamental rights and freedoms resilience for all 22 countries was 1456.3. Most countries in the dataset were more stringent than could be expected based on how liberal they usually are with the highest score for Portugal, Italy, Belgium, United Kingdom, Denmark, France, Germany, Spain, Switzerland, and the Netherlands (supplementary material 5). Whether this led to a pendulum effect that ultimately delayed responses during the second wave should be investigated. We observed that countries often maintained a high stringency level regardless of their epidemiological situation during the first wave, reflected in the low fundamental rights and freedoms recovery average of -1.9%, lower than health (-9.4%) and economic (5.3%) recovery rates.

No relationship was found between resilience of fundamental rights and freedom and neither resistance ($r(22) = .21$, $p = .34$) or recovery ($r(22) = .16$, $p = .47$). Furthermore, no relationship was found between resistance and recovery of fundamental rights and freedoms ($r(22) = .31$, $p = .17$). In other words, low economic resistance corresponded to a wide range of recovery rates. Various countries reacted strongly at first, suggesting weak resistance, but were also quick to remove their restrictions, such as Estonia which had the lowest resistance rates (20.24%) (the fastest escalation of strict measures), as well as the highest recovery (-3.47%), and the second-highest overall fundamental rights and freedoms resilience (1072.4). This was reflected by positively skewed data, evident for France, Norway, Estonia, and the Czech Republic who adopted more stringent measures earlier on but were quick to remove them by the summer. In contrast, Sweden which recorded the highest cumulative resilience of fundamental rights and freedoms of all countries showed a moderate resistance (9.1%) principally due to late measure introduction but low recovery (-0.6%). Negatively skewed data was evident for Great Britain, Germany, Spain, Greece, and Sweden who imposed and maintained more stringent measures later in the period. Other countries escalated quickly (low resistance) and maintained severe government interventions during the whole period, indicating a low recovery, such as Hungary, Germany, Portugal, or Denmark. These mixed results reflect the different strategies adopted by countries to deal with the COVID-19 during the first wave.

## Economic resilience

All countries experienced an economic decline, followed by recovery during the first wave, although to varying extents. Spain (445.61), Great Britain (383.62), France (341.1), and Italy (339.5) recorded



For peer review only - http://bmjopen.bmj.com/site/about/guidelines.xhtml

the greatest losses in terms of cumulative GDP to trend over the study period (area under the curve), while Denmark (93), Norway (95.4), Finland (103.8), and Switzerland (116.1) exhibited the lowest cumulative GDP losses. These losses in turn translated in negative gross rate and a departure from the previous trends. In this case, no country experienced similar values in the past five years suggesting that the robustness threshold was crossed for all 22 countries (supplementary material 3-4), even though governments usually supported their economy. The cumulative resilience average for all 22 countries is 223.66. All countries started their economic downturn in late February, apart from Finland, Greece, and Iceland a month after. For the 20 countries that had –both significant values for economic resilience and recovery (all except Slovenia and Slovakia), economic decline took on average 14 weeks to reach the economic trough. ’Economic resistance ($r(20) = .89$, $p < .001$) and recovery ($r(20) = .87$, $p < .001$) were found to be associated with overall economic resilience: lower resistance and faster recovery resulted in higher cumulative GDP losses. Countries with the highest resistance such as Denmark (-0.3%), Norway (-0.4%), Finland (-0.44%) also had some of the highest overall economic resilience, highlighting the important role of resistance on overall economic resilience. Economic resistance and economic recovery were negatively correlated meaning that lower resistance was associated with higher recovery rate ($r(20) = .85$, $p < .001$).

## Multisystemic resilience

Cross-sectoral comparisons of resilience results (mainly overall resilience) across the health, fundamental rights and freedoms, and economic dimensions allowed us to identify patterns, potential linkages and trade-offs between sectors and thereby derive a multisystemic understanding of a country’s resilience (Figure 2). Some countries may exhibit a high level of resilience in one domain but weak resilience in another. The time series graphs for all 22 countries combining measurements of the health, fundamental rights and freedoms, and economic dimensions of resilience are available in Supplementary file 7. Three countries – Iceland, Estonia, and Finland – were found to have high overall resilience in all three domains, closely followed by Norway, yet with slightly lower overall fundamental rights and freedoms resilience. On the other hand, Spain and Great Britain showed the lowest overall resilience in both the health and economic sectors and appeared strongly less liberal than usually they are in terms of the stringency of the response (supplementary material 5). They were surpassed by Portugal and Italy in overall fundamental rights and freedoms resilience. The comparative analysis shows more nuanced results with a significant tendency among all 22 European countries to favor health and economic sectors over fundamental rights and freedoms during the first wave of the COVID-19 pandemic in Europe in 2020, possibly related to the novel character of the pandemic and the importance given to the precautionary principle.

Of the nine countries with an excess mortality peak (low health resilience), Sweden, Spain, Great Britain, and the Netherlands presented the highest fundamental rights and freedoms resilience when compared with their excess mortality levels. Sweden showed the highest fundamental rights and freedoms resilience among all countries. In the case of Sweden, there was a large discrepancy between the stringency of the response and the excess mortality (supplementary material 5). On the other hand, France, Portugal, and Italy showed low fundamental rights and freedoms resilience relative to their epidemiological situation. Of the countries without an excess mortality peak, Hungary followed by Denmark, Germany, Greece, and Poland had the lowest fundamental rights and freedoms resilience to health. Because Hungary and Poland are illiberal compared to other countries, their response was not more stringent that we could expect in such countries. By contrast, Germany and Denmark were less liberal than they usually are and stringent in regard to their excess mortality.



For peer review only - http://bmjopen.bmj.com/site/about/guidelines.xhtml

Switzerland, Sweden, the Netherland, and Belgium demonstrated a high economic resilience compared to their health results suggesting stronger economic resilience. Conversely, France and Portugal exhibited low economic resilience compared to their health situation, while Spain, Great Britain and Italy showed moderate economic resilience relative to health. Among the low or no excess mortality countries, Hungary, Slovenia, and Slovakia demonstrated the weakest economic resilience (sharp economic decline), while Poland and Finland the highest economic resilience relative to health.

When comparing the fundamental rights and freedoms and the economic dimensions, more than half of the countries had lower cumulative economic losses (higher economic resilience) than fundamental rights and freedoms resilience. Poland and Switzerland registered the highest economic resilience compared to fundamental rights and freedoms resilience. This suggests that governments prioritized economic performance and that measures implemented to curtail the transmission of COVID-19 did not hinder countries' economies substantially. On the other hand, Spain and Sweden showed the highest fundamental rights and freedoms resilience compared to their economic situation. Similar findings were found by calculating correlation coefficients between the three sectors. Overall health resilience was associated with economic resilience ($r(22) = .69$, $p < .001$); in other words, countries with higher excess mortality also suffered greater economic losses. Health resilience appeared to be positively associated with overall fundamental rights and freedoms resilience but to a lesser extent ($r(22) = .56$, $p = .007$). This may be explained by the fact that many countries implemented stringent measures independently of their excess mortality trends. Overall economic resilience was found to be positively linked to fundamental rights and freedoms resilience ($r(22) = .67$, $p < .001$). The association of the economic domain with both the health dimension and the stringency of adopted measures may indicate its heavy reliance on, and importance to, other sectors' resilience.

Regarding robustness, only nine countries (Spain, Great Britain, Italy, Belgium, Switzerland, Netherlands, Portugal, France, and Sweden) had a discernable peak during the first wave. Although several countries were robust at the health system level, none of the 22 European countries were robust across all dimensions, as all 22 countries crossed economic and fundamental rights and freedoms robustness thresholds. This aligns with the idea that when resilience is provided by intervention (and not by design), there is transfer of capacities from other sectors [20]. Most countries experienced strong disruptions to fundamental rights and freedoms before experiencing economic decline and increase in excess mortality, suggesting that their fundamental rights and freedoms robustness levels tended to be lower than their economic and health robustness, like overall resilience results. Only Sweden, the Netherlands, Slovakia, Slovenia, and Estonia experienced their economic decline before the implementation of their measures, while for Switzerland it was simultaneous. As for the time lag between maxima, the lowest point of economic downturn occurred in most cases after the health and stringency index maximums. In addition, except for Finland, Iceland and Greece, all countries began their economic decline around the same time (24.02.2020). In the case of the COVID-19 pandemic, this suggests that economic robustness (the start of economic decline) may be influenced more by regional and global economic shifts than other country parameters such as health system performance and policy implementation. However, the unavailability of weekly data for the GDP ratio-to-trend, and the nature of the indicator itself, may bias these results.

## Discussion

This study is, to the best of our knowledge, the first attempt to operationalize the measurement of a multisystemic expression of resilience by assessing 22 European countries' resilience in three systems



For peer review only - http://bmjopen.bmj.com/site/about/guidelines.xhtml

– health, economy, and fundamental rights and freedoms. Our approach helps understand how countries are affected and respond to systemic crises by managing different societal objectives between sectors. Furthermore, while the recovery time has been used by various authors as the main resilience indicator [3], our operationalization of robustness, resistance and recovery in addition to an overall measure of resilience provide a more granular understanding of how countries were impacted and reacted to the first wave of the COVID-19 pandemic. For example, when considering only the recovery rate, the most resilient country would also be the one with the lowest resistance and the lowest overall resilience in our dataset (Spain).

Based on our results, we established four categories of country based primarily on health and economic resilience during the first wave: (1) high health & high economic resilience: Denmark, Finland, Iceland, Norway, Estonia, Poland, (2) high health & lower economic resilience: Hungary, Slovenia, Slovakia, Greece, Austria, Czech Republic, Germany, (3) higher economic & moderate health resilience: Switzerland, Sweden (4) low health & economic resilience, divided into two subcategories: (4.1) & higher fundamental rights and freedoms resilience: the Netherlands, Belgium, Great Britain, Spain, (4.2) & lower fundamental rights and freedoms resilience: Italy, Portugal, France. Figure 3 provides a graph for Norway, Hungary, Sweden, and Spain to illustrate the four categories. It should be noted that these categories are not prescriptive nor rigid divisions, but rather constantly evolving trends as resilience is an adaptive capacity. Countries are therefore likely to change category as the pandemic evolves.

Countries in category (1) were not been affected by the first wave of the Covid-19 pandemic to the same extent as countries with high peak mortality, possibly due to greater geographical isolation, as in the case of Iceland, or a less important caseload before measures were implemented which has been found to be an important factor in the literature [21]. This brings to fore one of the main limitations of our study, namely that different populations have not been similarly exposed to the first wave of COVID-19 and hence limited excess mortality, as in category (1), may not necessarily imply highest health resilience capacity. Nevertheless, all these countries still implemented moderate measures (with the most stringent restrictions for Poland) explained by fear of a new virus and as mimicry of neighboring countries where mortality rates were soaring. Estonia and Iceland, which adopted the least stringent measures also suffered slightly higher economic losses, potentially due to their greater dependence on the tourism industry [22]. Poland, however, is the only country in this group that showed weak fundamental rights and freedoms resilience supporting evidence of continued democratic backsliding during the pandemic [23, 24]. Poland's economic resilience may be attributed to its strong real economic growth rate in recent years.

All countries in category (2), although not having experienced any excess mortality peak, suffered more severe economic downturns resulting in part from the stricter measures implemented by governments. The heavier losses may also be related to greater dependence on tourism, as in Hungary, Greece, Austria, and Slovenia [22], as well as greater levels of economic dependence on foreign trade and exports to other European countries hit by less demand from abroad, as with Germany, the largest European exporter. This points to European countries' vulnerability to global economic shifts including international trade and supply chain disruptions. The pre-pandemic strength of the economy may also be an important determinant that warrants further research. All these countries showed lower fundamental rights and freedoms resilience than category 1. Hungary proved to be the least resilient in terms of measure stringency of this group (and of the comparative analysis),



For peer review only - http://bmjopen.bmj.com/site/about/guidelines.xhtml

where governments, similar to Poland, used the crisis to introduce tighter restrictions, deteriorating civil and political rights situation as well as media freedom [25].

Countries in category (3), despite higher excess mortality, showed strong economic resilience. For Sweden, the minimal restrictions adopted allowed businesses to continue operating. However, the economic losses suffered underline the country's economic dependence on exports. Sweden's unique no-lockdown model has indeed demonstrated the highest fundamental rights and freedoms resilience. Switzerland's economic resilience can be attributable to its diversified economy, substantial fiscal space available to support businesses during the crisis as well as highest economic preparedness among OECD countries [26]. Finally, Category 4 countries suffered from both health and economic downturns. Nevertheless, Italy, Portugal, and France proved to be less resilient in terms of fundamental rights and freedoms due in part to weaker crisis preparedness and potentially the balance of power towards the executive for France and Italy.

Our results provide further evidence that the COVID-19 pandemic tested not only the resilience of health systems but also economic and fundamental rights and freedom, but more interestingly that the resilience capacities of one system may depend on and impact the others. It is not surprising that highly affected health systems and the implementation of stringent containment measures notably lockdowns, border closures and travel bans, negatively impact a country's economic development, precipitating a trade decline and financial market volatility. Nevertheless, several countries enforced relatively strict measures while limiting economic consequences due to fewer cases and higher economic resilience. These non-uniform results suggest that the economic capacity to cope and recover from systemic shocks differs more widely between European countries and that countries' economic resilience does not solely relate to national measures, but also to the global and regional economic context.

The correlation between the economic domain with both the health and the fundamental rights and freedoms dimensions reflects both its heavy dependance on and importance to the other sectors' resilience. This correlation supports the need for current economic systems to be structurally transformed in ways that promote their self-organization around recovery to minimize resource requirements to better address global challenges [20]. In contrast, the effect of stringent government responses on health resilience is not as straightforward. Irrespective of their epidemiological situation, all governments adopted varying degrees of measures during the first wave of the COVID-19 pandemic in 2020, related to the novel character of the pandemic and precautionary principles. In addition, Lenton *et al.* found that prolonged stringent measures may not necessarily lead to improved health outcomes and may even reduce health resilience in the long term due to decreased trust in government over time [3].

Finally, our findings demonstrate a strong tendency among the 22 European countries to prioritize their health and economic systems over fundamental rights and freedoms in the first wave, except for Sweden. When considering strictly the health dimension, only seven countries crossed the health robustness threshold and all displayed high recovery capacities during the first wave, unlike other sectors. Among all peaking countries, recovery from excess mortality to pre-shock levels was in fact fastest when the system was less resistant. Whereas we could expect that lower resistance (higher peak) would lead to slower recovery (due to the difficulty of containing cases the more people are infected), a higher peak may result in faster and more effective measures as well as greater societal acceptance of these measures. This may also reflect the rapid depletion of people who were



For peer review only - http://bmjopen.bmj.com/site/about/guidelines.xhtml

susceptible to the disease when a significant proportion of the population was affected. By the end of August, no government could restore pre-crisis governance operating modes. This suggests that, for fear of the virus and unpreparedness for a pandemic, countries prioritized their health and economic sectors at the expense of fundamental rights and freedoms which are key to the functioning of liberal democracies in Europe. However, our indicator for fundamental rights and freedoms resilience (stringency of measures implemented), does not merely reflect the country's inability to absorb the shock and thus may limit the importance of these findings (further discussed below).

There are important limitations in the method used to assess multisystemic resilience. One of the main limitations of assessing resilience across countries is the issue of comparability; countries were not equally exposed to COVID-19, and did not start with the same context, and therefore high resilience results, as in category (1), do not necessarily imply the highest resilience capacity. In other words, our study measures in some ways how resilience capacities were expressed across different systems in relation to how they were exposed to the shock. Why and how it was expressed this way is another question that deserve more scrutiny. This depends both on the capacities of the countries and the capacities and willingness of the governance systems to mobilize these capacities. While stressing the multisystemic nature of resilience, we have limited our analysis to resilience in health, the economy and fundamental rights and freedoms. These systems were selected because they were more easily discerned and measurable, but systemic effects generated by the COVID-19 crisis have also disrupted social, governance and environmental systems amongst others [14]. Finally, our approach explores only limited aspects of the multiplicity in the ways resilience can be expressed. The use of time series analysis highlights the capacity to cope and recover while overlooking the process of adaptation and transformation that is intrinsically important to the study of resilience [14]. In addition to this limitation in the conceptualization of resilience, several other limitations can be identified.

The proxy Indicators used to measure health, fundamental rights and freedoms, and economic resilience have shortcomings. Regarding the GDP ratio-to-trend, the OECD stresses that it is more suitable for qualitative purposes to visualize the fluctuations of economic activity. Excess mortality was selected' as it overcomes several issues related to the reporting of COVID-19 related deaths such as miscounting from misdiagnosis or under-reporting. However, it does not only include 'direct' COVID-19 mortality as it also includes 'collateral damage' from other health conditions left untreated when the health system is overwhelmed. It also relies on the accuracy of mortality data from the five-year period before the crisis, which is more or less accurate from country to country. The OxCGRT's stringency index to measure the resilience of fundamental rights and freedoms reflects the breaches in civil liberties that are intrinsic to the values of liberal democracies. However, the indicator does not reflect the respect of democratic processes nor the rule of law, essential components to assess broader democratic resilience. Limitations also arise as this indicator is a composite of additive un-weighted indices that abstract away from nuances or any heterogeneity in the country responses. Its reliance on third party sources may also result in measurement bias [27]. Furthermore, unlike health and economic resilience, the stringency of measures does not simply reflect the 'country's inability to absorb and cope with the shock but also its ability to implement and modify measures quickly. This may explain the varying results between resistance, recovery, and overall resilience, as in the case of Estonia, whose resistance was the lowest but overall resilience was also among the second highest after Sweden. A more accurate estimate of the fundamental rights and freedoms resilience would require a longer study period to track the evolution of measures throughout the pandemic and beyond



For peer review only - http://bmjopen.bmj.com/site/about/guidelines.xhtml

to examine whether countries leveraged the pandemic to shift towards a more authoritarian system, as it seems to be the case of Poland or Hungary.

There were additional limitations regarding the method used for measurements. First, slight changes in the method used for the calculation of resistance and recovery yields noticeably different results. Second, robustness has not been often operationalized or integrated into resilience measurement within the literature. We defined a country as robust on the health and economic dimensions when able to maintain usual (pre-crisis) performance. With this approach, all countries were thus considered robust before the onset of the COVID-19 pandemic. Measuring health robustness exclusively with excess mortality is a simplification because the reduction in the provision of healthcare services may have a time delay. This is why evidence of disruptions of health services is quite important. This shortcoming reflects the difficulty of operationalizing the robustness of complex systems, for which further research is needed. Third, we did not compute a unique multisystemic measure of resilience encompassing all sectors as this would have required a normative judgment on whether one system should be considered more important than another. This is fundamentally a political choice. While we recognize that directly comparing resilience in health, fundamental rights and freedom, and the economy can be challenging from a public health perspective, we believe that it is the role of science to look at difficult questions of trade-offs. Such assessment can help foster a democratic debate about the values in our societies and help select where investment needs to be made to increase our capacities to prevent such shocks.

Finally, there need to be some reflections about the applicability of the methodological approach. Given the fact that the COVID-19 pandemic has not been a single event but recurring waves of different magnitude, we believe that our approach should be applied to study resilience to the different waves of the pandemic. This will lead to an understanding of whether and how countries adapt their priorities and strategies over time. Ideally, assessing resilience should be based on several indicators in each sector. Because of the decoupling between excess mortality and infections thanks to immunization, one needs an indicator that reflects the decoupling between mortality and infection. Another concern will be to expand the number of countries covered in the analysis. While the selected indicators are available for applicable to all countries, interpretations of the results should also consider the variety of cultural values and political system in the world. In complement to what was done in this paper, there also opportunities to use other methodological approaches such as interrupted times series to better relate what happened during the first wave of the COVID-19 pandemic and previous trends [28]. Based on our approach to measure resilience, a related key issue will be to understand whether, over the duration of the COVID-19 pandemic, it makes more sense to escalate and deescalate quickly or whether it is more relevant to keep similar levels of measures when it comes to an event that is likely to have recurring patterns and last for some time.

## Conclusion

This study serves as a methodological contribution to better understand how the first wave of the COVID-19 pandemic tested not only the capacity of health systems but also the economy and fundamental rights and freedoms. Our findings demonstrated a strong tendency among the 22 European countries to prioritize their health and economic systems over fundamental rights and freedoms during the first wave of the COVID-19 pandemic that hit Europe in spring 2020. Overall, our assessment of multisystemic resilience to gauge expressed capacities of several European countries



For peer review only - http://bmjopen.bmj.com/site/about/guidelines.xhtml

14.03.2023

to tackle the first wave of the COVID-19 pandemic provides an important step for understanding the capacities of countries to cope, adapt and transform can be expressed during a shock.

By exploring the relationships between robustness, resistance, and recovery, our study draws our attention the dynamics of resilience in complex systems. With limited pandemic preparedness capacities, it appears clearly that no country was robust in all three dimensions. A lack of capacity to rapidly control the pandemic was thus compensated by strong restrictions on individual rights and freedoms. Economic resilience was also found to be significantly linked to health outcomes as well as the stringency of measures, suggesting the dependence on, and importance to, the other sector'' resilience. Furthermore, recovery varied widely both across the three dimensions and across countries. Overall, this suggests that resilience by interventions implies trade-offs between systems with different objectives. While more research is needed on multisystemic resilience, our paper ultimately sheds light on the relevance of the study of resilience to improve governance capacities to prevent, react to, and recover from the current and future systemic shocks.

## Ethics Statement

This study is based on online search of publicly available data. No patient, nor the public were involved in the design of the study. No ethical approval was sought as per our university requirements.

## Contributors:

DW, MC and AR conceptualized the multisystemic measurement approach to resilience. MC and AR analysed the data and developed the successive drafts of the paper. MC and AR designed the figures and tables. MC, AR, FT, CM, YK, NL and DW contributed content and comments to the paper. DW and NL are both principal investigators within the COVID-19 systemic crisis project.

## Competing Interests:

None declared

## Funding:

This research was funded by the Swiss National Science Foundation (SNSF), within the scope of the COVID-19 systemic crisis project (Grant 31CA30_196396, https://data.snf.ch/covid-19/snsf/196396)

## Data Sharing Statement

All data used for this study are available in a public, open access repository. The sources of the data are the following 1) health resilience: the Human Mortality Database [Reference 15], 2) resilience of fundamental rights and freedoms: Oxford COVID-19 Government Response Tracker, Blavatnik School of Government, University of Oxford (CC BY License) [Reference 16], 3) economic resilience: OECD Main Economic Indicators Publication [Reference 17].



For peer review only - http://bmjopen.bmj.com/site/about/guidelines.xhtml

14.03.2023

# References


1. Linkov, I., et al., *Changing the resilience paradigm.* Nature Climate Change, 2014. **4**(6): p. 407-409.
2. Wernli, D., et al., *Governance in the age of complexity: building resilience to COVID-19 and future pandemics*. 2021, Geneva Science Policy Interface, University of Geneva, Switzerland.
3. Lenton, T.M., C.A. Boulton, and M. Scheffer, *Resilience of countries to COVID-19 correlated with trust.* Scientific Reports, 2022. **12**(1): p. 75.
4. Haldane, V., et al., *Health systems resilience in managing the COVID-19 pandemic: lessons from 28 countries.* Nature Medicine, 2021.
5. Arsenault, C., et al., *COVID-19 and resilience of healthcare systems in ten countries.* Nature Medicine, 2022.
6. Saulnier, D.D., et al., *A health systems resilience research agenda: moving from concept to practice.* BMJ Global Health, 2021. **6**(8): p. e006779.
7. Helbing, D., *Globally networked risks and how to respond.* Nature, 2013. **497**(7447): p. 51-59.
8. Ungar, M., *Multisystemic resilience: Adaptation and transformation in contexts of change*. 2021: Oxford University Press, USA.
9. Folke, C., et al., *Resilience thinking: integrating resilience, adaptability and transformability.* Ecol. Soc., 2010. **15**: p. 20.
10. Pimm, S.L., et al., *Measuring resilience is essential to understand it.* Nature Sustainability, 2019. **2**(10): p. 895-897.
11. Grafton, R.Q., et al., *Realizing resilience for decision-making.* Nature Sustainability, 2019. **2**(10): p. 907-913.
12. Galaitsi, S.E., et al., *The Need to Reconcile Concepts that Characterize Systems Facing Threats.* Risk Analysis, 2021. **41**(1): p. 3-15.
13. Schiller, C., et al., *Just How Resilient are OECD and EU Countries? Sustainable Governance in the Context of the COVID-19 Crisis*. 2021, Bertelsmann Stiftung.
14. Wernli, D., et al., *Building a multisystemic understanding of societal resilience to the COVID-19 pandemic.* BMJ Glob Health, 2021. **6**(7): p. e006794.
15. [Dataset]. *Human Mortality Database [Internet]*. University of California, Berkeley (USA), and Max Planck Institute for Demographic Research (Germany) [cited 2021 16 August]; Available from: https://www.mortality.org/.
16. [Dataset]. *COVID-19 Government Response Tracker [Internet]*. [cited 2021 16 August]; Available from: https://www.bsg.ox.ac.uk/research/research-projects/covid-19-government-response-tracker.
17. [Dataset] OECD. *Main Economic Indicators Publication [Internet]*. [cited 2021 16 August]; Available from: https://stats.oecd.org/Index.aspx?DataSetCode=MEI.
18. Youngs, R., *COVID-19 and democratic resilience.* Glob Policy, 2022.
19. Lewkowicz, J., M. Woźniak, and M. Wrzesiński, *COVID-19 and erosion of democracy.* Econ Model, 2022. **106**: p. 105682.
20. Hynes, W., et al., *Systemic resilience in economics.* Nature Physics, 2022. **18**(4): p. 381-384.
21. Haug, N., et al., *Ranking the effectiveness of worldwide COVID-19 government interventions.* Nature Human Behaviour, 2020.
22. OECD, *OECD Tourism Trends and Policies 2020*. 2020.
23. Lewkowicz, J., M. Woźniak, and M. Wrzesiński, *COVID-19 and erosion of democracy.* Economic modelling, 2022. **106**: p. 105682-105682.
24. International Institute for Democracy and Electoral Assistance, *The State of Democracy in Europe 2021 - Overcoming the impact of the pandemic*. 2021.
25. Bene, M. and Z. Boda, *Hungary: Crisis as Usual—Populist Governance and the Pandemic*, in *Populism and the Politicization of the COVID-19 Crisis in Europe*, G. Bobba and N. Hubé, Editors. 2021, Springer International Publishing: Cham. p. 87-100.
26. OECD, *OECD Economic Surveys: Switzerland 2022*. 2022.




For peer review only - http://bmjopen.bmj.com/site/about/guidelines.xhtml

14.03.2023

27. Hale, T., et al., *Government responses and COVID-19 deaths: Global evidence across multiple pandemic waves.* PLOS ONE, 2021. **16**(7): p. e0253116.

28. Wambua, S., et al., *The indirect impact of COVID-19 pandemic on inpatient admissions in 204 Kenyan hospitals: An interrupted time series analysis.* PLOS Global Public Health, 2021. **1**(11): p. e0000029.

Figure legends/caption

Figure 1 Conceptual framework of resilience and its sub-components: robustness, resistance, and recovery inspired by Grafton et al. [11]

Figure 2 Overall comparison of resilience (area under the curve) of the Health system, Fundamental Rights and Freedoms, and Economic system.

Figure 3 Time series analysis of selected countries representing the 4 categories and combining resilience measurements of the Health system, Fundamental Rights and Freedoms, and Economic system

For peer review only

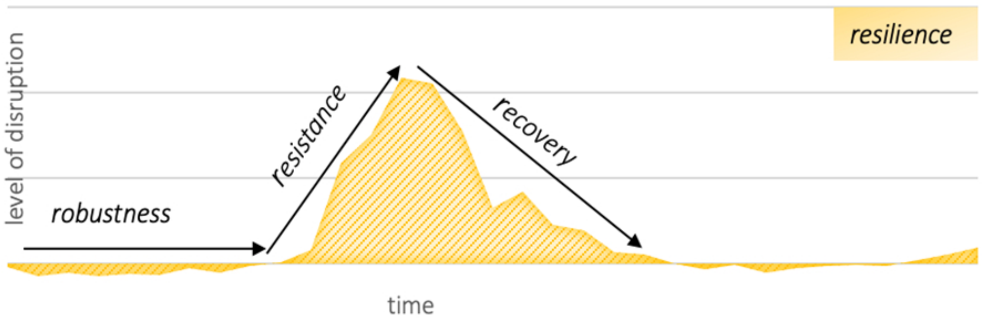


Conceptual framework of resilience and its sub-components: robustness, resistance, and recovery inspired by Grafton et al. [11]

402x139mm (57 x 57 DPI)

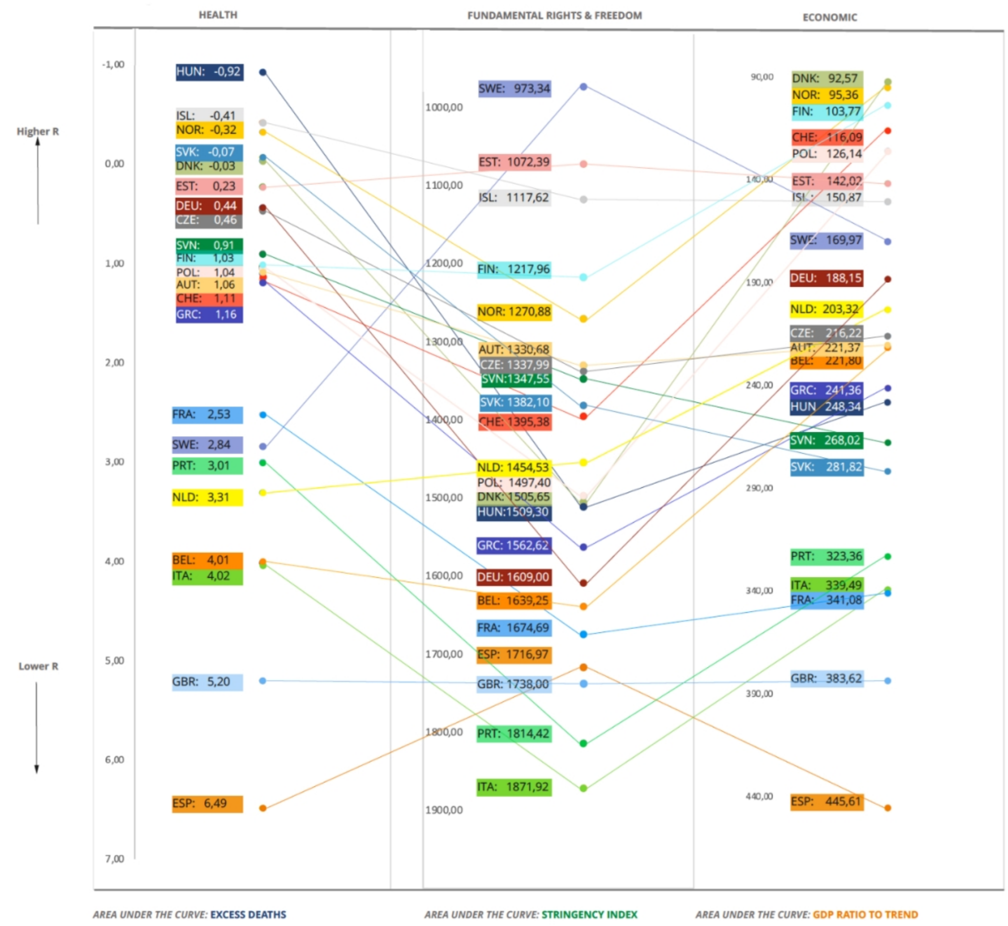


Overall comparison of resilience (area under the curve) of the health, fundamental rights and freedoms, and economic domains.

402x383mm (87 x 87 DPI)

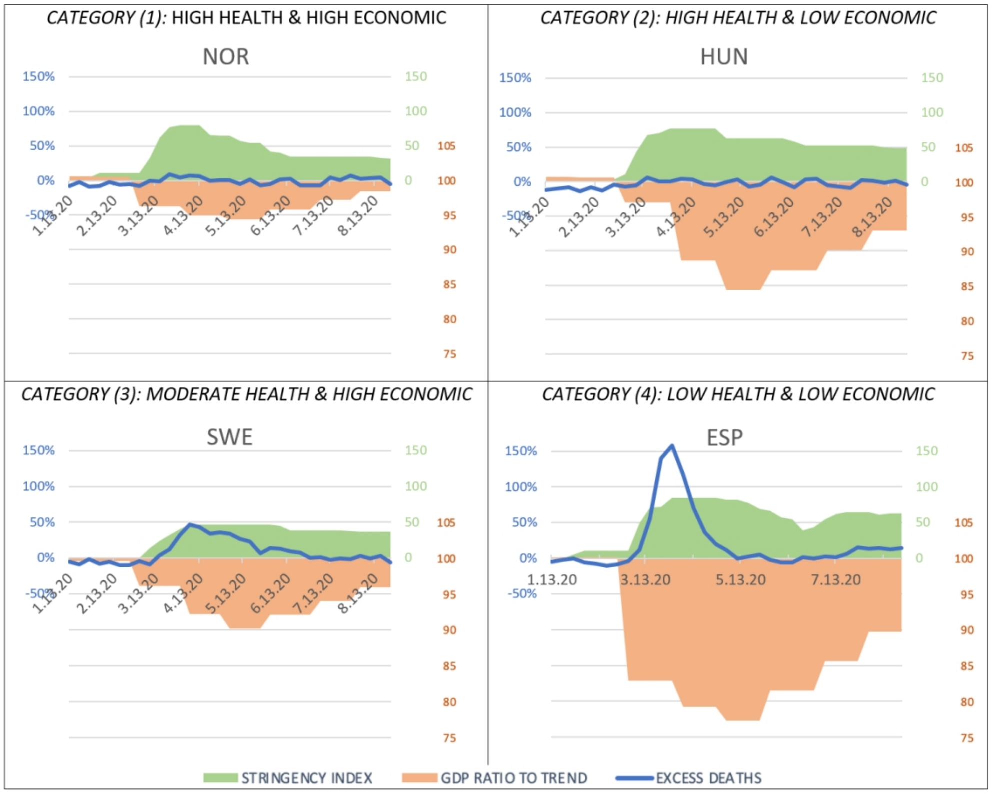


Figure 3 Time series analysis of selected countries representing the 4 categories and combining resilience measurements of the health, fundamental rights and freedoms, and economic domains.

681x551mm (57 x 57 DPI)

# Measuring multisystemic resilience to shocks: health, fundamental rights and freedoms, and economic resilience during the COVID-19's first wave in Europe

## Supplementary material

**Supplementary material 1 – Average excess mortality for week 3 – 35 from 2015 – 2020 for 22 selected countries**

| Country Name | 2015 | 2016 | 2017 | 2018 | 2019 | 2020 | 2020 Z-score |
|---|---|---|---|---|---|---|---|
| UK | 0,78% | -1,32% | -0,45% | 2,39% | -1,40% | 15,88% | 1,9932 |
| Spain | 3,39% | -3,59% | -1,28% | 2,15% | -0,32% | 19,79% | 1,9508 |
| Belgium | 2,90% | -2,95% | -0,99% | 2,51% | -0,81% | 12,07% | 1,8554 |
| Netherlands | -1,59% | -2,31% | -1,13% | 4,14% | 1,24% | 10,05% | 1,7665 |
| France | 0,14% | -3,21% | -1,43% | 2,41% | 2,42% | 7,62% | 1,6633 |
| Italy | 4,90% | -5,48% | 0,82% | -1,76% | 1,97% | 12,16% | 1,6622 |
| Sweden | 3,08% | -0,96% | 0,29% | 2,52% | -4,61% | 8,45% | 1,5899 |
| Portugal | -0,85% | -0,76% | -4,41% | 5,05% | 1,52% | 9,30% | 1,5711 |
| Finland | -2,67% | -1,21% | -0,11% | 4,27% | 0,07% | 3,02% | 0,9424 |
| Austria | 3,56% | -5,79% | -1,03% | 2,39% | 1,36% | 3,29% | 0,7471 |
| Slovenia | -0,08% | -4,60% | 1,17% | 1,85% | 2,44% | 2,56% | 0,7411 |
| Switzerland | 4,76% | -5,13% | -2,01% | 0,51% | 2,38% | 3,29% | 0,7232 |
| Poland | -0,27% | -5,80% | -0,80% | 4,35% | 3,02% | 3,13% | 0,6758 |
| Greece | | -5,00% | 2,77% | -2,30% | 5,22% | 3,63% | 0,6397 |
| Czechia | 2,40% | -5,59% | -1,46% | 4,00% | 1,32% | 1,38% | 0,3043 |
| Germany | | -4,96% | -0,67% | 5,93% | 0,45% | 1,32% | 0,2319 |
| Estonia | -1,30% | -2,99% | 1,41% | 3,90% | -0,17% | 0,74% | 0,1996 |
| Denmark | -2,03% | -2,93% | -1,18% | 6,31% | 0,16% | -0,11% | -0,0435 |
| SVK | 3,88% | -5,88% | -0,53% | 2,38% | 0,80% | -0,23% | -0,0899 |
| Norway | 0,59% | -2,06% | -0,33% | 2,24% | -0,19% | -1,15% | -0,6762 |
| Hungary | 4,50% | -5,78% | 1,22% | 0,89% | -0,08% | -3,04% | -0,7405 |
| Iceland | -0,85% | 0,77% | 1,91% | 0,41% | 0,64% | -1,33% | -1,3500 |



For peer review only - http://bmjopen.bmj.com/site/about/guidelines.xhtml

Clausin, Rieckhoff, …, Wernli

**Supplementary material 2 – Box plots and whisker (1.5 IQR) for robustness in health systems measured as the average of excess mortality during week 3 to 35 for the year 2015 – 2020.**

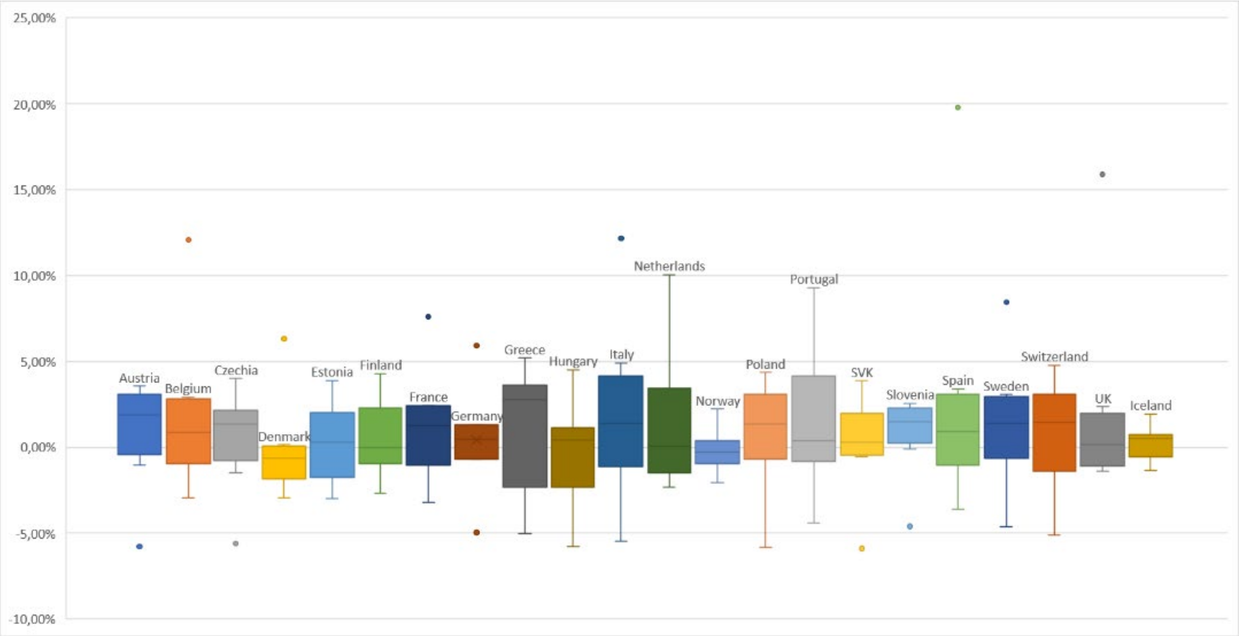


**Supplementary material 3 – Robustness in economic system based on economic growth from year 2015 to 2020 based on World Bank Data.**

| Country Name | 2015 | 2016 | 2017 | 2018 | 2019 | 2020 | 2020 Z-score |
|---|---|---|---|---|---|---|---|
| United Kingdom | 2,39 | 2,17 | 2,44 | 1,71 | 1,60 | -11,03 | -2,04 |
| Italy | 0,78 | 1,29 | 1,67 | 0,93 | 0,48 | -9,04 | -2,03 |
| Spain | 3,84 | 3,04 | 2,98 | 2,28 | 1,98 | -11,33 | -2,03 |
| Belgium | 2,04 | 1,27 | 1,62 | 1,79 | 2,24 | -5,36 | -2,03 |
| France | 1,11 | 1,10 | 2,29 | 1,87 | 1,84 | -7,78 | -2,03 |
| Portugal | 1,79 | 2,02 | 3,51 | 2,85 | 2,68 | -8,30 | -2,02 |
| Netherlands | 1,96 | 2,19 | 2,91 | 2,36 | 1,96 | -3,89 | -2,02 |
| Austria | 1,01 | 1,99 | 2,26 | 2,43 | 1,52 | -6,45 | -2,02 |
| Greece | -0,20 | -0,49 | 1,09 | 1,67 | 1,88 | -9,00 | -1,98 |
| Iceland | 4,44 | 6,30 | 4,19 | 4,89 | 2,42 | -6,84 | -1,97 |
| Slovenia | 2,21 | 3,19 | 4,82 | 4,45 | 3,45 | -4,32 | -1,96 |
| Germany | 1,49 | 2,23 | 2,68 | 0,98 | 1,06 | -3,70 | -1,95 |
| Czechia | 5,39 | 2,54 | 5,17 | 3,22 | 3,03 | -5,50 | -1,95 |
| Hungary | 3,71 | 2,20 | 4,27 | 5,36 | 4,86 | -4,55 | -1,95 |
| Denmark | 2,34 | 3,25 | 2,82 | 1,99 | 1,49 | -1,99 | -1,93 |
| Switzerland | 1,64 | 2,07 | 1,36 | 2,86 | 1,14 | -2,38 | -1,92 |
| Poland | 4,38 | 2,95 | 5,14 | 5,95 | 4,45 | -2,02 | -1,92 |
| Slovak Republic | 5,17 | 1,94 | 2,94 | 4,03 | 2,52 | -3,37 | -1,88 |
| Sweden | 4,49 | 2,07 | 2,57 | 1,95 | 1,99 | -2,17 | -1,83 |
| Finland | 0,54 | 2,81 | 3,19 | 1,14 | 1,22 | -2,21 | -1,72 |
| Norway | 1,97 | 1,07 | 2,32 | 1,12 | 0,75 | -0,72 | -1,69 |
| Estonia | 1,85 | 3,16 | 5,79 | 3,78 | 3,74 | -0,55 | -1,64 |

**Supplementary material 4 – Robustness in economic system**

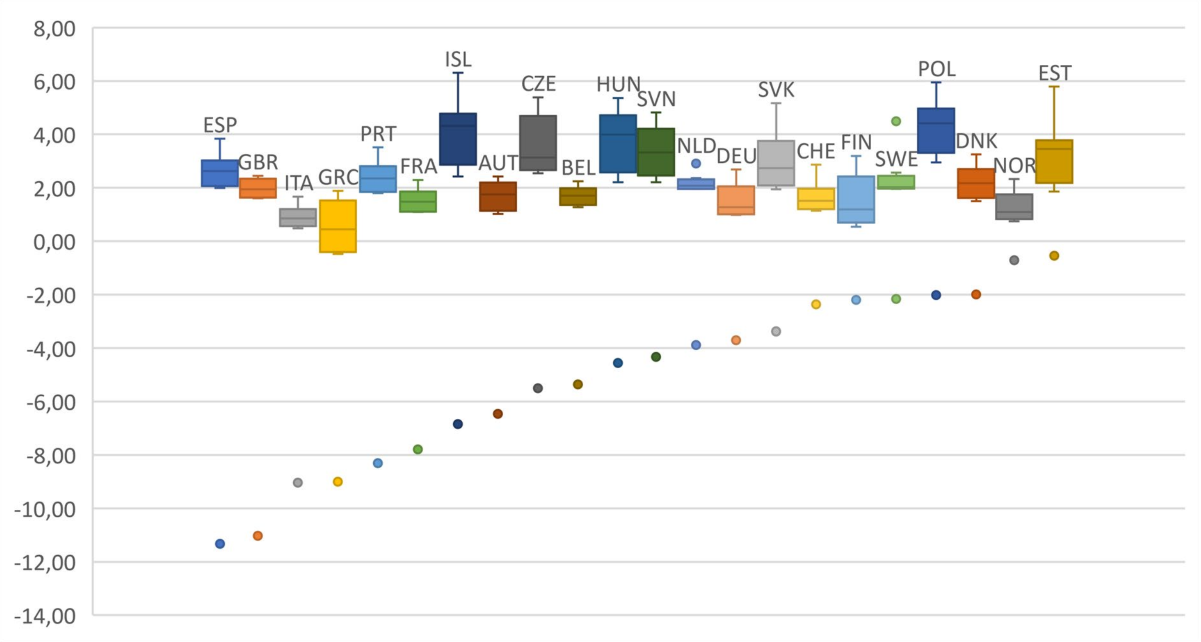


**Supplementary material 5 – Multisystemic resilience.**

Methods: to compare the overall balance in terms of resilience in different systems, we used data from the Liberal Democracy Index from V-Dem (https://v-dem.net/) from 2015 to 2020 and calculated average for each country in the dataset. We calculate the average among all countries, standard deviation and derived Z-score for each country. The same procedure was done for excess mortality. We calculated two different scores from these measurements. The first is the difference between excess mortality and stringency gives an indication of the adequation between the problem and response. It relies on the assumption that a higher stringency index is politically more justifiable in case of higher excess mortality (it must be noted that the precautionary principle may impose to take some measure to protect public health). A negative score suggests that there was not enough response compared to the magnitude of the problem (during the first wave) while a highly positive score suggest that the response was overtly stringency relative to other countries. The second score is the difference between the state of liberal democracy and the stringency index which gives an idea about the departure from the traditional way of governing. This score is based on the assumption that a liberal country may be more likely to protect fundamental rights and freedoms even in times of emergency. A negative score suggests that a country that it traditionally liberal was more stringent that one would have expected to be compared to other European countries during the first wave of the COVID-19.

Results:

| Country | Normalized Z-Score liberal democracy | Normalized Z-score stringency | Normalized Z-score-excess-mortality | EM - SI | ID - SI |
|---|---|---|---|---|---|



For peer review only - http://bmjopen.bmj.com/site/about/guidelines.xhtml

Clausin, Rieckhoff, …, Wernli

| | | | | | |
|---|---|---|---|---|---|
| Austria | 1,09 | 1,54 | 2,10 | -0,55 | -0,46 |
| Belgium | 0,55 | 2,85 | 3,21 | -0,36 | -2,30 |
| Czechia | 1,43 | 1,59 | 1,65 | -0,06 | -0,16 |
| Denmark | 0,00 | 2,24 | 1,31 | 0,93 | -2,24 |
| Estonia | 0,40 | 0,45 | 1,55 | -1,10 | -0,05 |
| Finland | 0,56 | 1,06 | 2,29 | -1,24 | -0,50 |
| France | 0,71 | 2,93 | 3,01 | -0,08 | -2,22 |
| Germany | 0,47 | 2,68 | 1,58 | 1,10 | -2,21 |
| Greece | 1,20 | 2,51 | 1,99 | 0,52 | -1,32 |
| Hungary | 4,53 | 2,23 | 0,61 | 1,62 | 2,29 |
| Iceland | 0,97 | 0,67 | 0,00 | 0,67 | 0,30 |
| Italy | 0,90 | 3,87 | 3,01 | 0,86 | -2,97 |
| Netherlands | 0,57 | 2,00 | 3,12 | -1,11 | -1,44 |
| Norway | 0,28 | 1,30 | 0,67 | 0,63 | -1,02 |
| Poland | 2,81 | 2,22 | 2,03 | 0,19 | 0,60 |
| Portugal | 0,43 | 3,50 | 2,92 | 0,58 | -3,07 |
| Slovak Republic | 1,62 | 1,75 | 1,26 | 0,49 | -0,14 |
| Slovenia | 1,06 | 1,66 | 2,09 | -0,43 | -0,60 |
| Spain | 1,09 | 3,17 | 3,30 | -0,13 | -2,08 |
| Sweden | 0,00 | 0,00 | 2,94 | -2,94 | 0,00 |
| Switzerland | 0,31 | 1,81 | 2,07 | -0,26 | -1,51 |
| United Kingdom | 0,88 | 3,17 | 3,34 | -0,17 | -2,29 |

In many, the response to the COVID-19 pandemic introduced a significant departure from previous

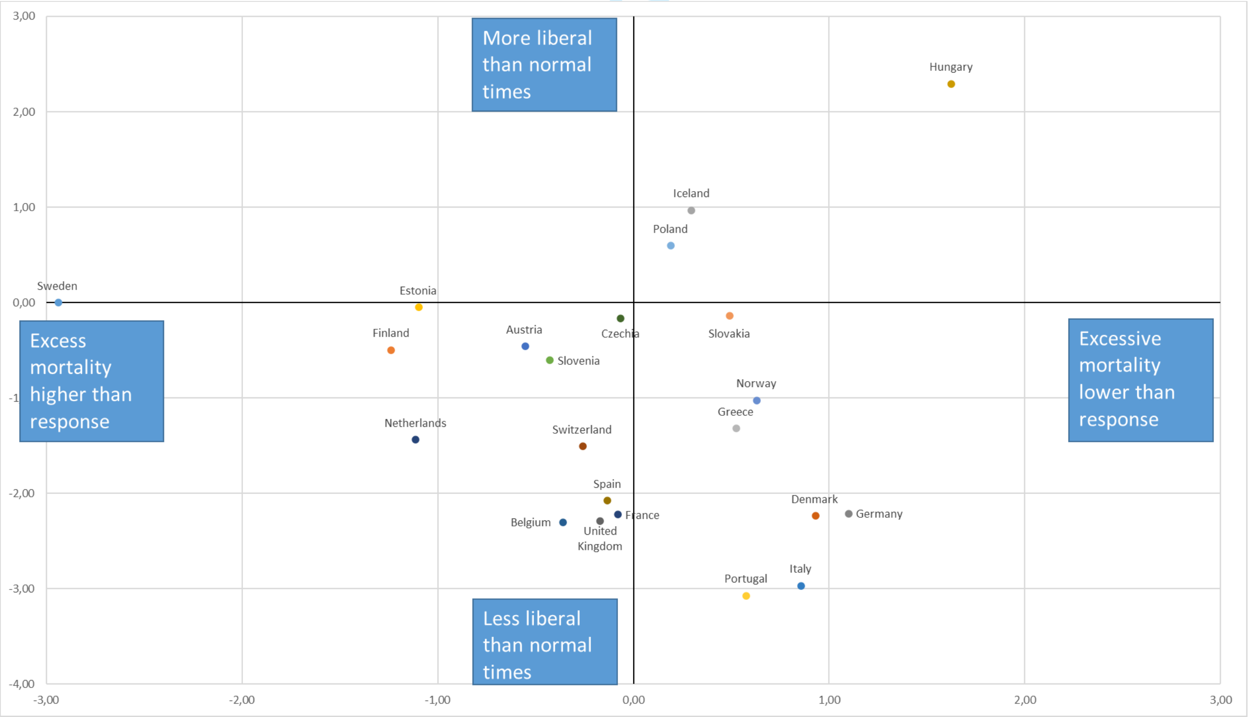




For peer review only - http://bmjopen.bmj.com/site/about/guidelines.xhtml

## Supplementary material 6 – Overall results regarding the measurements of resilience

| | Health system (%point/week) | | | | Fundamental rights and freedoms (points/week) | | | | Economic system (points/week) | | | |
|---|---|---|---|---|---|---|---|---|---|---|---|---|
| **Countries** | **Area** | **Resistance** | **Recovery** | **Robustness** | **Area** | **Resistancee** | **Recovery** | **Robustness** | **Area** | **Resistance** | **Recovery** | **Robustness** |
| **AUT** | 1.06 | 3.16% (>0.05) | -4.86% (<0.01) | Yes | 1330.68 | 19.47 (<0.05) | -3.75 (<0.001) | No | 221.38 | -0.84 (<0.001) | 0.69 (<0.001) | No |
| **BEL** | 4.01 | 29.89% (<0.01) | -14.67% (<0.01) | No | 1639.25 | 7.68 (<0.01) | -2.54 (<0.001) | No | 221.81 | -0.91 (<0.001) | 0.82 (<0.001) | No |
| **CHE** | 1.11 | 14.5% (<0.01) | -9.6% (<0.001) | Yes | 1395.38 | 15.54 (<0.01) | -3.64 (<0.001) | No | 116.10 | -0.68 (<0.001) | 0.44 (<0.001) | No |
| **CZE** | 0.46 | 2.84% (<0.05) | -2.23% (>0.05) | No | 1337.99 | 7.51 (<0.001) | -3.51 (<0.001) | No | 216.23 | -1.15 (<0.001) | 0.43 (<0.001) | No |
| **DEU** | 0.44 | 4,96% (<0.05) | -1.73% (<0.01) | No | 1609.00 | 6.61 (<0.001) | -1.05 (<0.01) | No | 188.16 | -0.94 (<0.001) | 0.59 (<0.001) | No |
| **DNK** | -0.03 | 5.33% (n/a) | -9.18% (n/a) | Yes | 1505.65 | 16.53 (<0.001) | -1.17 (<0.001) | No | 92.58 | 0.3 (<0.001) | 0.27 (<0.001) | No |
| **ESP** | 6.49 | 45.28% (<0.05) | -26.23% (<0.001) | No | 1716.97 | 8.64 (<0.001) | -5.42 (<0.05) | No | 445.61 | -1.37 (<0.01) | 0.94 (<0.001) | No |
| **EST** | 0.23 | 4,94% (>0.05) | -5,37 (>0.05) | Yes | 1072.39 | 20.24 (<0.05) | -4.14 (<0.001) | No | 142.02 | -0.61 (<0.001) | 0.26 (<0.001) | No |
| **FIN** | 1.03 | 2,5% (>0.05) | -9.34% (n/a) | Yes | 1217.97 | 6.54 (<0.001) | -2.51 (<0.001) | No | 103.78 | -0.44 (<0.001) | 0.29 (<0.001) | No |
| **FRA** | 2.53 | 17.29% (<0.01) | -8.3% (<0.01) | No | 1674.69 | 8.52 (<0.001) | -4.98 (<0.001) | No | 341.09 | -1.64 (<0.001) | 1.13 (<0.001) | No |
| **GRC** | 1.16 | 7.99% (>0.05) | -8.22% (>0.05) | Yes | 1562.62 | 17.06 (<0.001) | -4.59 (<0.001) | No | 241.37 | -1.00 (<0.001) | 0.30 (<0.001) | No |
| **HUN** | -0.92 | 10.23% (n/a) | -6.22 (n/a) | Yes | 1509.30 | 15.08 (<0.001) | -1.33 (<0.001) | No | 248.35 | -1.51 (<0.001) | 0.66 (<0.001) | No |
| **ISL** | -0.41 | 9.45% (>0.05) | 44.63% (n/a) | Yes | 1117.62 | 4.25 (<.001) | -2.79 (0.001) | No | 150.88 | -0.76 (<0.001) | 0.33 (<0.001) | No |
| **ITA** | 4.02 | 23.68% (<0.01) | -10.77% (<0.001) | No | 1871.92 | 8.65 (<0.001) | -5.96 (<0.05) | No | 339.49 | -1.57 (<0.001) | 0.98 (<0.001) | No |
| **NLD** | 3.31 | 20.52% (<0.01) | -10.73% (<0.001) | No | 1454.53 | 15.59 (<0.01) | -3.60 (<0.001) | No | 203.32 | -0.85 (<0.001) | 0.46 (<0.001) | No |
| **NOR** | -0.32 | 10,3% (n/a) | -5,37% (n/a) | Yes | 1270.88 | 8.02 (<0.001) | -2.22 (<0.001) | No | 95.36 | -0.40 (<0.01) | 0.32 (<0.001) | No |
| **POL** | 1.04 | 2.47% (>0.05) | -11.68% (n/a) | Yes | 1497.40 | 7.31 (<0.001) | -4.63 (<0.01) | No | 126.14 | -0.76 (<0.001) | 0.47 (<0.001) | No |
| **PRT** | 3.01 | 5.11% (<0,01) | -3.35% (<0.01) | No | 1814.42 | 7.66 (<0.001) | -1.80 (<0.001) | No | 323.36 | -1.21 (<0.05) | 0.89 (<0.001) | No |
| **SVK** | -0.07 | 5.64% (>0.05) | -9.68% (n/a) | Yes | 1382.10 | 9.03 (<0.001) | -2.87 (<0.001) | No | 281.83 | -13.76 (n/a) | 0.37 (<0.001) | No |
| **SVN** | 0.91 | 19.55% (n/a) | -5.69% (<0.01) | Yes | 1347.55 | 19.91 (<0.001) | -6.47 (<0.001) | No | 268.03 | -17.17 (n/a) | 0.55 (<0.001) | No |
| **SWE** | 2.84 | 13.80% (<0.05) | -3.74% (<0.001) | No | 973.34 | 9.10 (<0.001) | -0.62 (<0.01) | No | 169.98 | -0.71 (<0.001) | 0.44 (<0.001) | No |
| **UK** | 5.20 | 28.23% (<0.01) | -12.63% (<0.001) | No | 1738.00 | 6.09 (<0.01) | -0.76 (<0.05) | No | 383.63 | -1.68 (<0.001) | 1.01 (<0.001) | No |



For peer review only - http://bmjopen.bmj.com/site/about/guidelines.xhtml

Abbreviations: Austria AUT, Belgium BEL, Czechia CZE, Denmark DNK, Estonia EST, Finland FIN, France FRA, Germany DEU, Greece GRC, Hungary HUN, Iceland ISL, Italy ITA, Netherlands NLD, Norway NOR, Poland POL, Portugal PRT, Slovak Republic SVK, Slovenia SVN, Spain ESP, Sweden SWE, Switzerland CHE, United Kingdom UK.

For peer review only



For peer review only - http://bmjopen.bmj.com/site/about/guidelines.xhtml

**Supplementary material 7 – Time series analysis for 22 countries combining measurements about the health, fundamental rights and freedoms, and economic dimensions of resilience**

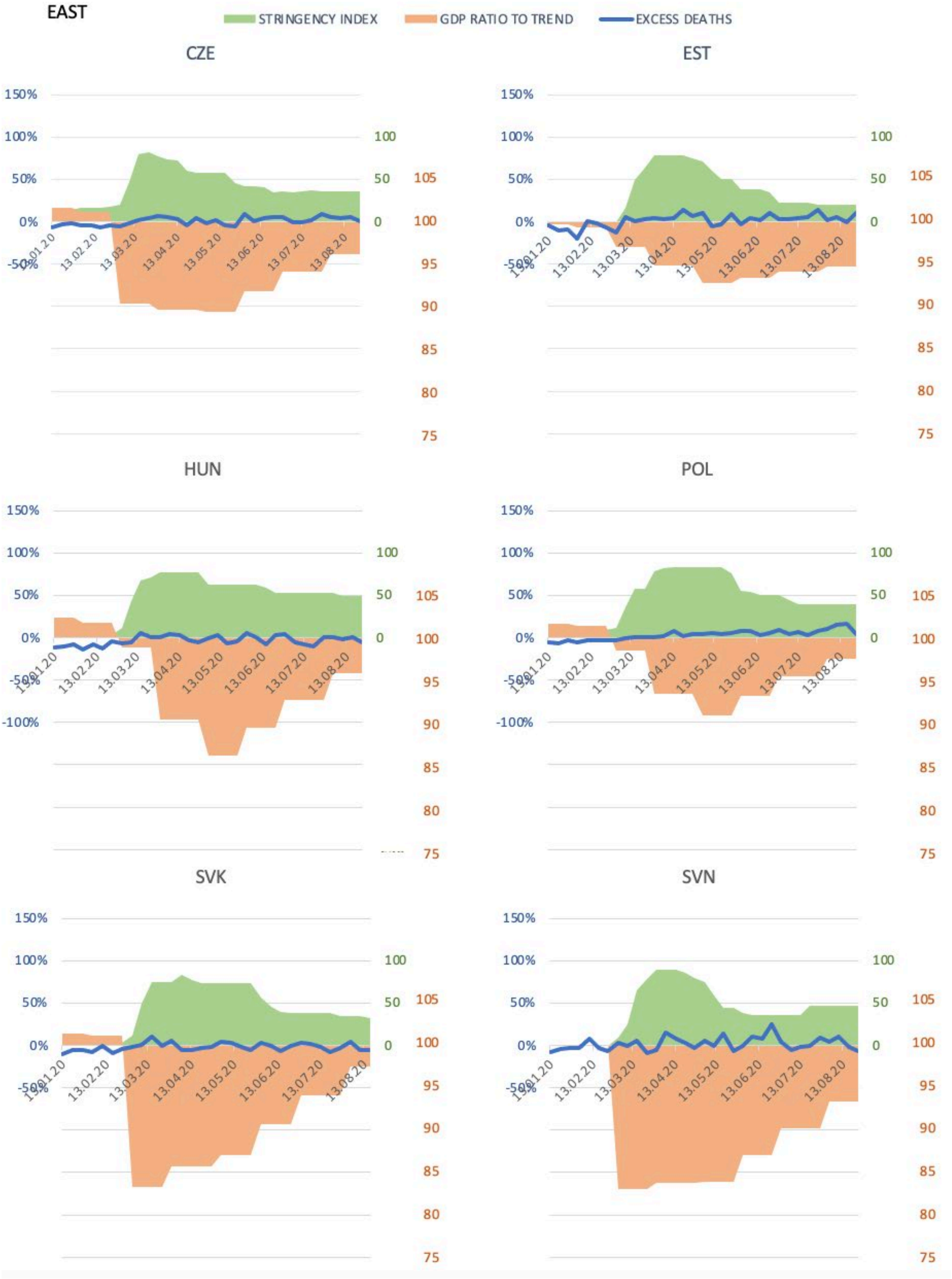

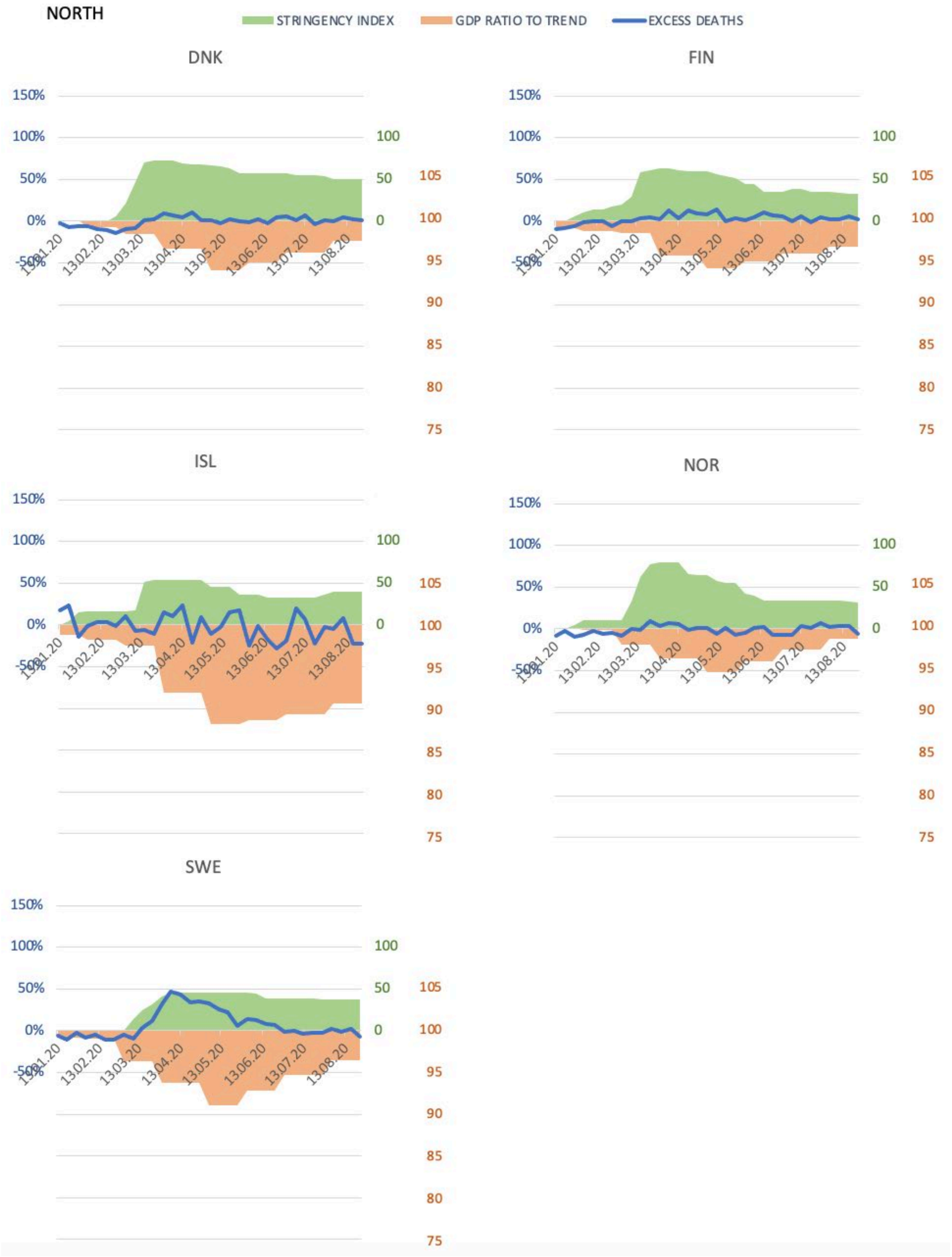

NORTH
STRINGENCY INDEX
GDP RATIO TO TREND
EXCESS DEATHS
DNK
FIN
ISL
NOR
SWE
150%
100%
50%
0%
-50%
100
50
0
105
100
95
90
85
80
75
13.01.20
13.02.20
13.03.20
13.04.20
13.05.20
13.06.20
13.07.20
13.08.20

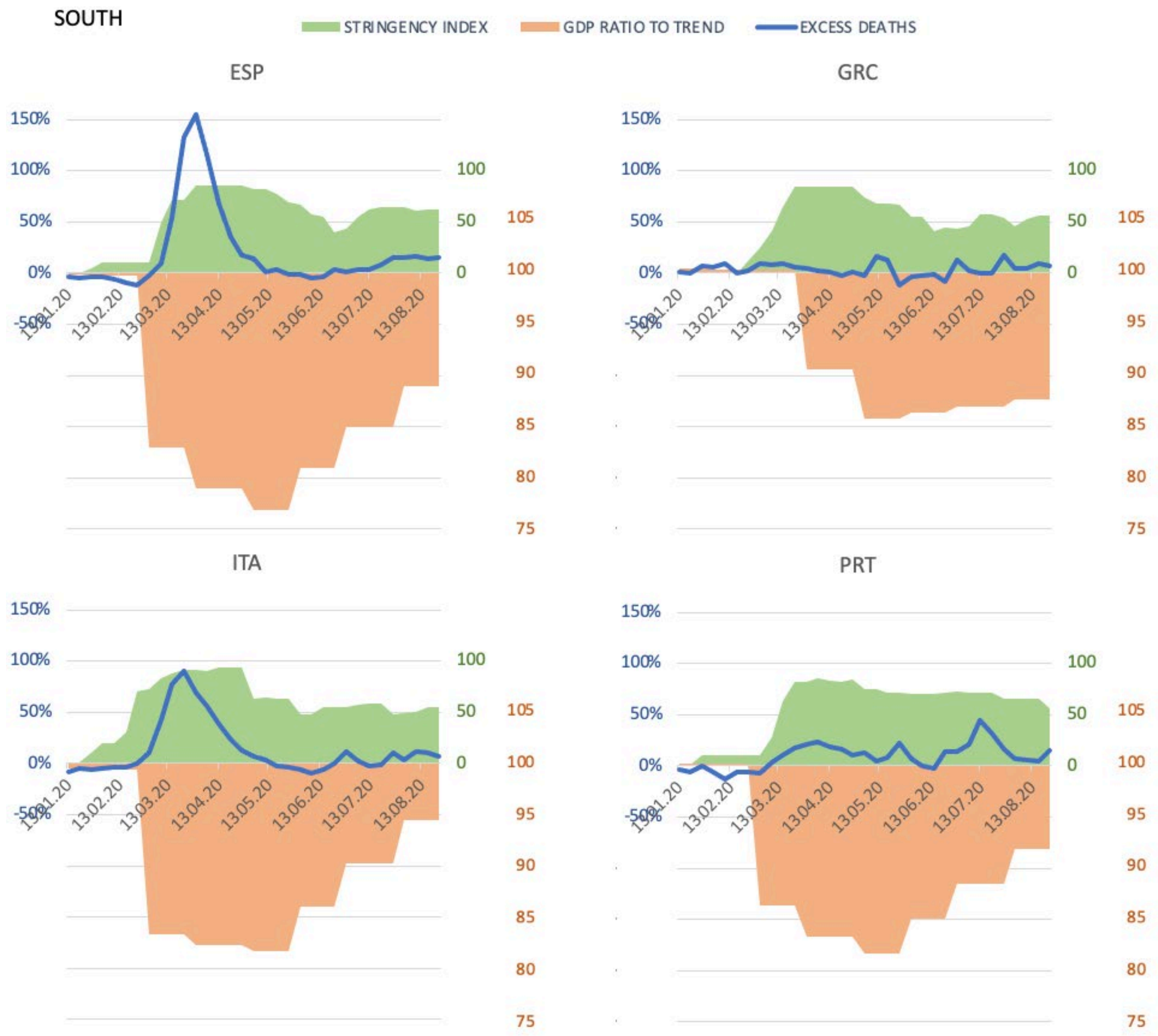
SOUTH
STRINGENCY INDEX
GDP RATIO TO TREND
EXCESS DEATHS
ESP
GRC
ITA
PRT
150%
100%
50%
0%
-50%
100
50
0
105
100
95
90
85
80
75
13.01.20
13.02.20
13.03.20
13.04.20
13.05.20
13.06.20
13.07.20
13.08.20

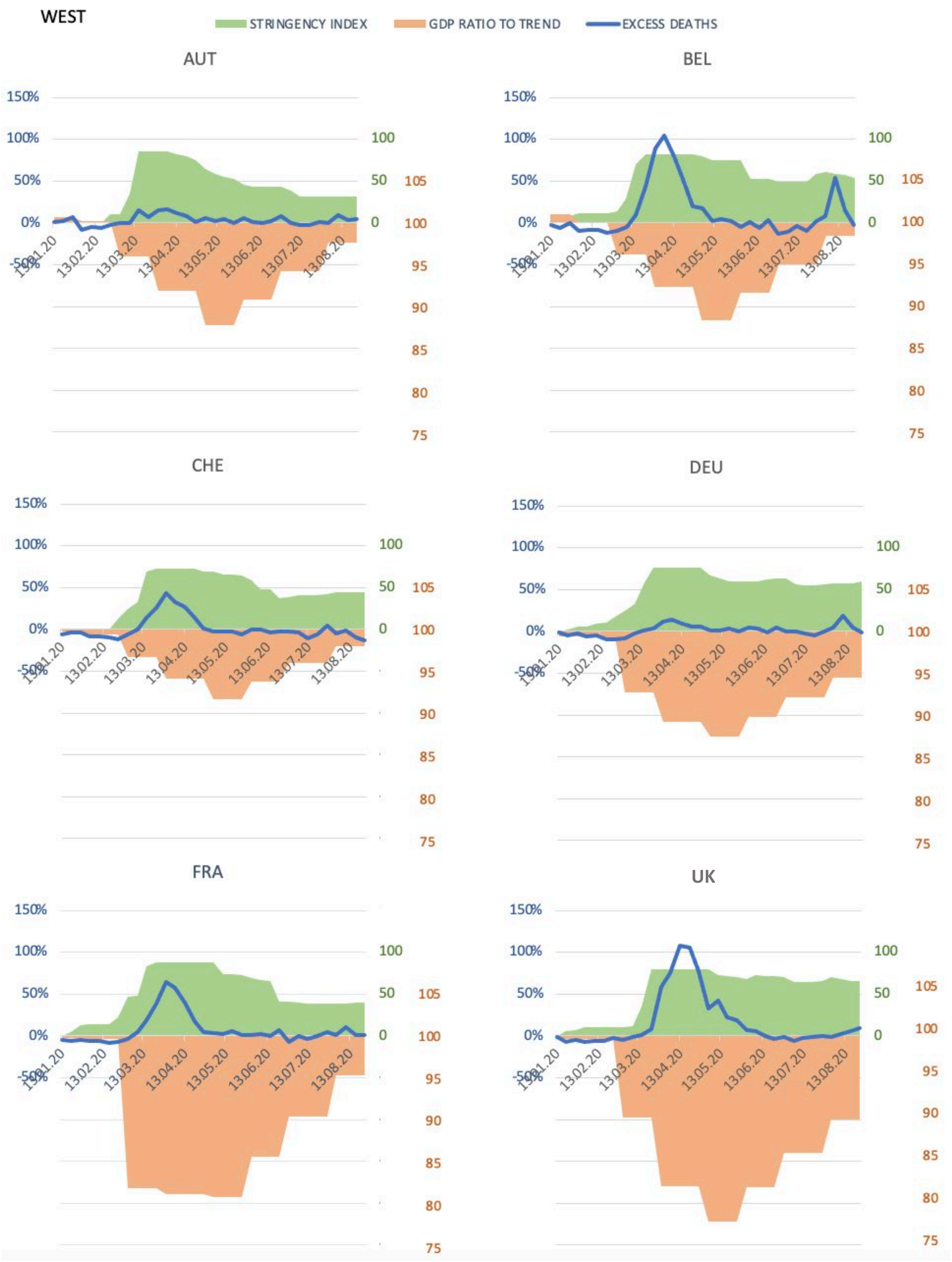




For peer review only - http://bmjopen.bmj.com/site/about/guidelines.xhtml

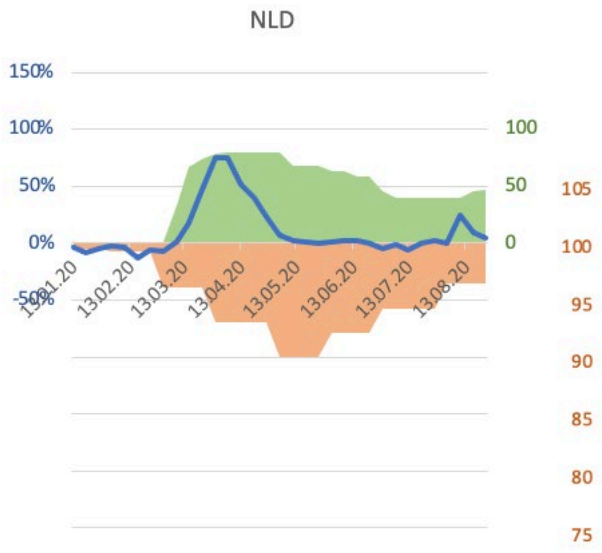


For peer review only

BMJ Open

# Multisystemic resilience to shocks: a temporal analysis of health, fundamental rights and freedoms, and economic resilience during the first wave of the COVID-19 pandemic in 22 European countries



| | |
|---|---|
| Journal: | *BMJ Open* |
| Manuscript ID | bmjopen-2022-065445.R2 |
| Article Type: | Original research |
| Date Submitted by the Author: | 15-Jun-2023 |



| | |
|---|---|
| Complete List of Authors: | Clausin, Mia; University of Geneva, Global Studies Institute<br>Rieckhoff, Alicia; University of Geneva, Global STudies Institute<br>Tediosi, Fabrizio; Swiss Tropical and Public Health Institute, Household Economics & Health Systems Research Unit<br>Morel, Chantal; University of Geneva, Global Studies Institute<br>Kaspiarovich, Yuliya; University of Geneva, Global Studies Institute<br>Levrat, Nicolas; University of Geneva, Global Studies Institute; Faculty of Law, University of Geneva<br>Wernli, Didier; University of Geneva, Global Studies Institute; University of Geneva Faculty of Science, Department of computer science |



| | |
|---|---|
| <b>Primary Subject Heading</b>: | Health policy |
| Secondary Subject Heading: | Global health, Infectious diseases |
| Keywords: | COVID-19, PUBLIC HEALTH, INFECTIOUS DISEASES |
| | |


SCHOLARONE™ Manuscripts

*I, the Submitting Author has the right to grant and does grant on behalf of all authors of the Work (as defined in the below author licence), an exclusive licence and/or a non-exclusive licence for contributions from authors who are: i) UK Crown employees; ii) where BMJ has agreed a CC-BY licence shall apply, and/or iii) in accordance with the terms applicable for US Federal Government officers or employees acting as part of their official duties; on a worldwide, perpetual, irrevocable, royalty-free basis to BMJ Publishing Group Ltd ("BMJ") its licensees and where the relevant Journal is co-owned by BMJ to the co-owners of the Journal, to publish the Work in this journal and any other BMJ products and to exploit all rights, as set out in our licence.*

*The Submitting Author accepts and understands that any supply made under these terms is made by BMJ to the Submitting Author unless you are acting as an employee on behalf of your employer or a postgraduate student of an affiliated institution which is paying any applicable article publishing charge ("APC") for Open Access articles. Where the Submitting Author wishes to make the Work available on an Open Access basis (and intends to pay the relevant APC), the terms of reuse of such Open Access shall be governed by a Creative Commons licence – details of these licences and which Creative Commons licence will apply to this Work are set out in our licence referred to above.*

*Other than as permitted in any relevant BMJ Author's Self Archiving Policies, I confirm this Work has not been accepted for publication elsewhere, is not being considered for publication elsewhere and does not duplicate material already published. I confirm all authors consent to publication of this Work and authorise the granting of this licence.*

For peer review only

15.06.2023

# Multisystemic resilience to shocks: a temporal analysis of health, fundamental rights and freedoms, and economic resilience during the first wave of the COVID-19 pandemic in 22 European countries

Mia Clausin[1*], Alicia Rieckhoff[1*], Fabrizio Tediosi[2-3], Chantal Morel[1], Yuliya Kaspiarovich[1], Nicolas Levrat[1], Didier Wernli[1]

[1] Geneva Transformative Governance Lab, Global Studies Institute, University of Geneva, Switzerland
[2] Swiss Tropical and Public Health Institute, Basel, Switzerland
[3] University of Basel, Basel, Switzerland

* These authors contributed equally.

Keywords: resilience, robustness, recovery, multisystemic, health systems, governance, complexity, systemic risks, systemic crisis, COVID-19

Corresponding author: Didier.Wernli@unige.ch

Word count: 5004 in the main text. 2 tables, 3 figures, 8 supplementary materials. 26 references.

Abstract:

**Objectives:** Research on resilience to the COVID-19 pandemic has primarily focused on health system resilience. The purpose of this paper is to 1) develop a broader understanding of societal resilience to shocks by evaluating resilience in three systems: health, economic, and fundamental rights and freedoms and 2) to further operationalize resilience in terms of robustness, resistance and recovery.

**Settings:** 22 European countries were selected based on the availability of data in the health, fundamental right and freedoms and economic systems during the first wave of the COVID-19 pandemic.

**Design:** This study uses time series data to assess resilience in health, fundamental right and freedoms and economic systems. An overall resilience was estimated, as well as three of its sub-components: robustness, resistance, and recovery.

**Results:** Six countries exhibited an outlier excess mortality peak compared to the pre-pandemic period (2015-2019). All countries experienced economic repercussions and implemented diverse measures affecting individual rights and freedoms. Three main groups of countries were identified: 1) high health and high or moderate economic and/or fundamental rights and freedoms resilience, (2) moderate health and fundamental rights and freedoms resilience, and (3) low resilience in all three systems.

**Conclusions:** The classification of countries into three groups provides valuable insights into the multifaceted nature of multisystemic resilience during the first wave of the COVID-19 pandemic. Our study highlights the importance of considering both health and economic factors when assessing resilience to shocks, as well as the necessity of safeguarding individual rights and freedoms during times of crisis. Such insights can inform policy decisions and aid in the development of targeted strategies to enhance resilience in the face of future challenges.



For peer review only - http://bmjopen.bmj.com/site/about/guidelines.xhtml

15.06.2023

For peer review only



For peer review only - http://bmjopen.bmj.com/site/about/guidelines.xhtml

15.06.2023

Strengths and limitations of this study:

- The study operationalizes measurements of resilience of 22 European countries to the first wave of the COVID-19 pandemic in three systems – health, economic, and fundamental rights and freedoms.
- Three resilience components – robustness, resistance, and recovery – in addition to an overall measure of resilience have been measured, and different measurements methods have been tested.
- The primary limitation when assessing resilience lies in country comparability (countries were not exposed to COVID-19 in the same way) and the interpretation of resilience.
- Selected indicators only partially capture resilience, overlooking other factors influencing countries' ability to absorb and adapt to a shock.
- While measurement of resilience remains in need of further research, the various measurement methods tested showed significant variations in results.

# 1 Introduction

The COVID-19 pandemic has tested the resilience of countries to deal with a shock affecting health, economic, social, environmental, and governance systems worldwide [1]. In an early 21st century characterized by uncertainty, complexity, and systemic disruptions, resilience thinking has gained traction in various fields including disaster management, the environment, and more recently global health [2, 3]. While current research on resilience to COVID-19 primarily focuses on health systems, [4-6], there is a need to assess resilience beyond health systems [7]. Considering resilience as a multi-objective societal problem, the concepts of 'systemic resilience' [8] and 'multisystemic resilience' [9] have emerged to better understand the impact of shocks across highly interconnected systems.

There are currently different definitions of resilience across fields ranging from the capacity of a system to come back to its initial state to the capacity "*to absorb disturbance and reorganise while undergoing change so as to still retain essentially the same function, structure, identity, and feedbacks*" [10]. Resilience thinking draws attention to both the vulnerability of systems and the capacity to respond. However, with no universally accepted definition across systems, the measurement of resilience remains underdeveloped [11]. To address this gap, this paper starts from the assumption that some indicators of performance of a system can be used as proxy for the expression of its resilience capacities. The second assumption is that the resilience of a system over time can be divided into different components such as robustness, resistance, and recovery [12, 13].

While the policy literature has also sought to develop measurement of multisystemic resilience [14], This is a cross-country study depicting the first wave of the COVID-19 pandemic in 22 European countries measuring health resilience, economic resilience and resilience of fundamental rights and freedoms. This study provides insights about how different systems are affected by the COVID-19 pandemic. Understanding resilience as the ability of societies to maintain their core governance functions while minimising the undesirable societal effects can serve as the foundation for an integrated approach to build societal resilience to future pandemics [1].

# 2 Methods

## 2.1 Public and Patient Involvement

This study is based on publicly available data about the impacts of the COVID-19 pandemic on health, individual rights and freedoms, and the economy (the detail of the data used is provided in the section



For peer review only - http://bmjopen.bmj.com/site/about/guidelines.xhtml

below). No patient, nor the public were involved in the design of the study. No ethical approval was sought as per our university requirements. The STROBE guidelines were used for organizing the method section [15].

## 2.2 Study design and setting

This study investigates the temporal dynamics of resilience of three systems, i.e., health, individual rights and freedoms, and the economy, during the initial wave of the COVID-19 pandemic in Europe. An extensive review of academic and intergovernmental sources was conducted to identify relevant indicators of resilience. This search encompassed sources such as the World Bank, World Health Organization, OECD, and European Union. Indicators were selected based on two key criteria: availability of weekly or monthly data capturing temporal evolution, and relevance to the specific type of resilience being examined (see the measurements section below). Data collection spanned from week 3, before the implementation of any COVID-19 measures, to week 35, when COVID-19 deaths approached their lowest levels across European countries (January 13, 2020, to August 31, 2020). The analysis focuses on twenty-two European countries chosen based on the availability of data pertaining to the three selected systems. Countries lacking data for any of the three systems at the time of data collection were excluded from the analysis. The collected data for each indicator was systematically compiled in separate spreadsheets for each country.

## 2.3 Data sources and selected indicators

The indicator selected for measuring health system resilience was weekly excess mortality, from the Human Mortality Database [16]. A death is recorded by week of occurrence, apart from the UK, where death by week of registration is used. Excess mortality was selected as it helps overcome several issues related to the reporting of COVID-19 related deaths such as miscounting from misdiagnosis or under-reporting. It also includes 'collateral damage' from other health conditions left untreated if the health system is overwhelmed [6]. Excess mortality was defined as the number of deaths registered in excess of the five-year average from 2015 to 2019. The calculations for Germany and Greece were based on the four-year average (2016-2019) due to unavailable data for 2015. A weekly P-score, which was chosen as it allows for robust country comparisons, was calculated as the percentage difference between the reported and projected number of deaths. Health system resilience was interpreted as the inverse of excess mortality. The lower the excess mortality, the larger health system resilience.

The indicator selected for fundamental rights and freedoms' resilience was the Oxford Coronavirus Government Response Tracker's (OxCGRT) stringency index [17]. The stringency index records the strictness of 'lockdown style' policies that restrict people's behaviour. The index is a composite measure of categorical variables based on nine indicators: school closing, workplace closing, cancelling of public events, restrictions on the size of gatherings, closing of public transport, stay-at-home requirements, restrictions on internal movement, restrictions on international travel and public information campaigns. It therefore provides a proxy indicator to measure restrictions in fundamental rights and freedoms, considering that the latter (e.g., freedom of movement; right to assembly and demonstration) are intrinsic to the functioning of liberal democracies. While the indicator is a daily value between 1 and 100 (100 = strictest), a weekly average was derived for this study. Resilience of fundamental rights and freedoms was measured as the inverse of the stringency index. This means that the more stringent was the response, the less resilient were fundamental rights and freedoms.



For peer review only - http://bmjopen.bmj.com/site/about/guidelines.xhtml

15.06.2023

To measure economic resilience, the OECD's short-term main economic indicator: Gross Domestic Product (GDP) ratio to trend was used [18]. It is a monthly derived indicator where GDP, which is a monetary measure of the market value of all goods and services produced and sold in a specific period, is divided by the long-term GDP trend to give a ratio-to-trend. It is notably useful to visualise economic cycles. If the GDP ratio to trend = 100, GDP is equal to long-term GDP trends. If the GDP ratio to trend is higher than 100, GDP is higher than long-term GDP trends which signified economic expansion. If the GDP ratio to trend is below 100, the GDP is lower than the long-term GDP trend signalling that the 'growth' (if any) is less than trend. GDP ratio to trend data were compared to quarterly GDP, i.e., the percentage change from the same quarter in previous years, to check for its accuracy in representing the magnitude of the shock. Economic resilience to the shock was interpreted as the inverse of the decline of the GDP ratio.

## 2.4 Measurements of resilience, robustness, resistance, and recovery

Table 1 outlines the definitions used for resilience and its 3 components across the different systems and Figure 1 provides a visual representation of these concepts. Table 2 outlines the criteria used to measure health, fundamental rights and freedoms, and economic resilience, robustness, resistance, and recovery. Overall resilience in health, fundamental rights and freedoms and economic systems was estimated using the cumulative value for the selected measurements. A highly positive value (for excess mortality and stringency index) or negative value (for GDP ratio to trend) means that a country lacks resilience. By contrast, a negative excess mortality value means that the country is exceeding normal times performance (resilience overshot). Data points used to calculate the area under the GDP ratio to trend curve were computed as the point difference to trend (if x=97, then 100-97=3 was coded). Results were then used to conduct a comparative analysis across systems and identify general trends and potential trade-offs between them. This allowed us to analyse the resilience level relative to other systems' results and minimise bias caused by different time exposure to the virus from one country population to another.

*Table 1 Resilience to COVID-19 and its applications to health, fundamental rights and freedoms, and the economy based on resilience and three core subcomponents (inspired by Grafton et al. [12])*

| Definitions | Health system | Fundamental rights and freedoms system | Economic system |
|---|---|---|---|
| **Resilience** is the capacity of a system to withstand, absorb, and recover from a disturbance | Capacity to meet significant increases in demand for both public health and healthcare services and adapt to long-standing changes | Capacity to preserve and protect individual rights and freedoms and recover quickly after a shock | Capacity to limit the magnitude of economic losses, recover quickly, and forge new developmental paths for prosperity |
| ***Robustness*** is the capacity of a system to maintain its identity/performance and not cross an undesirable threshold following an adverse event. | Capacity to maintain health services and population health within the range of normal variation | Capacity to maintain respect for individual rights and freedoms within the range of normal variation | Capacity to maintain economic indicators performance within the range of normal variation |
| ***Resistance*** is the capacity to absorb disruption with minimal damage to system functionality | Capacity to slow down COVID-19 transmission and excess mortality | Capacity to minimize an escalation of stringent measures violating fundamental rights and freedoms | Capacity to minimize economic disruption and impact of the shock |



For peer review only - http://bmjopen.bmj.com/site/about/guidelines.xhtml

15.06.2023

| | | | |
|---|---|---|---|
| ***Recovery*** is capacity to 'bounce-back' following disruption | Capacity to recover to a scenario of pre-pandemic excess mortality rates | Capacity to end emergency measures that impact fundamental rights and freedoms | Capacity to recover to a scenario with pre-pandemic GDP trends and avoid a long-term recession |

*Table 2 Measurements and interpretation of resilience, robustness, resistance, and recovery across the health – fundamental rights and freedoms, and economic systems*

| System | Resilience | Robustness | Resistance rate | Recovery rate |
|---|---|---|---|---|
| Health system | Cumulative excess mortality between 13.01.20 – 31.08.20. A low value is indicative of high level of resilience | The value for the period considered is not an outlier for the period 2015-2020 | Slope of linear regression between value closest to 0 and maximum. A low positive value is indicative of high resistance | Slope of linear regression between maximum to first value closest to zero. A high negative value is interpreted as a high level of recovery |
| Fundamental rights and freedoms system | Cumulative stringency index value between 13.01.20 – 31.08.20. A low value is indicative of high level of resilience | The value for the period considered is an outlier compared to 2015-2020 | Slope of linear regression between value closest to 0 and maximum. A low positive value is indicative of high resistance | Slope of linear regression between maximum and minimum value. A high negative value is interpreted as a high level of recovery |
| Economic system | Cumulative GDP ratio to trend losses between 13.01.20 – 31.08.20. A low value is indicative of high level of resilience | The value for the period considered is an outlier compared to 2015-2020 | Slope of linear regression between value closest to 0 and minimum. A low negative value is indicative of high resistance | Slope of linear regression between minimum and maximum value. A high positive value is interpreted as a high level of recovery |

Robustness primarily means that a system continues to work at the same level of performance despite a disturbance. For example, the definition of health robustness is the capacity to maintain the same level of health services in case of increased demand [6]. Finding a precise threshold of disruption is challenging as it depends on the system affected and may involve arbitrary considerations. A suitable approach to assess robustness is to evaluate whether the system has continued to demonstrate a level of performance that is in line with previous years. In this study, the 1.5 interquartile range method was selected to identify outliers regarding the indicators of health and economic systems resilience. Countries that had values above the 1,5 interquartile range during the first wave of the COVID-19 pandemic were considered as not robust. Regarding fundamental rights and freedoms, the situation is different as the Oxford stringency index was introduced with the pandemic and there is no point of comparison with the period preceding the pandemic.

Resistance and recovery were estimated using the slope of linear regressions leading to the peak (resistance) and away from the peak (recovery). We used F-test to assess the statistical significance of the calculated slopes. To select the relevant data for the calculation of a slope, a continuous upward or downward trend was defined as the presence of no more than one subsequent value that does not follow the upward or downward trend. Regarding resistance, we used the interval of data from a minimum (the selected start points was the negative or positive value closest to 0 ) to the maximum or peak during the period considered from week 3 to 35 [4]. For countries that experienced two or more 'peaks', we selected the first peak (as the goal of the paper is to assess resilience at the onset of the pandemic). This means that different countries may have different starting and ending points. A higher slope value (in %) represents a lower level of resistance. For economic resistance, the slope was calculated from the value closest to 100 to the lowest value. Recovery was measured using the slope of the linear regression over the data interval between the maximum value (minimal value for



For peer review only - http://bmjopen.bmj.com/site/about/guidelines.xhtml

the economic indicator) and the minimal subsequent value. A higher slope value (in %) represents a faster level of recovery.

## 2.5 Statistical analysis

We conducted an evaluation of association among the measurements of resilience across multiple systems. Firstly, we calculated Pearson correlation coefficients for each system to examine the potential correlation between resistance, recovery, and overall resilience. While we successfully computed an overall resilience measure for each country across the three systems, certain combinations of systems and countries did not yield statistically significant results ($p > .05$) regarding the correlation between resistance, recovery, and overall resilience. Consequently, these countries were excluded from the calculation of associations between resilience and resistance/recovery within the specific system. Secondly, we calculated Pearson correlation coefficients to assess whether overall resilience within one system exhibited correlations with resilience in other systems. To facilitate further comparison of resilience across the different systems, we utilized z-scores to derive two additional metrics. The first metric quantifies the disparity between excess mortality and response stringency, providing an indication of the appropriateness of the response relative to the magnitude of the problem. The second metric measures the deviation from the conventional approach to governance by assessing the difference between an index measuring the state of liberal democracy and the stringency index. The methodology employed for deriving these metrics can be found in supplementary material 1.

# 3 Results

## 3.1 Health system resilience

During the first wave in Europe, up to 31 August 2020, six countries exhibited a cumulative excess mortality that was an outlier compared to 2015-2019 (supplementary material 2 and 3) and were considered as not robust. While the remaining countries were considered as robust during the first wave, three of them experienced peaks above 40% (Netherlands 75%, Switzerland 45%, and Portugal 44%). Several countries reported at least two non-consecutive values above 10% of excess mortality. Among them only Portugal had two statistically significant peaks. Eight of the nine countries with a mortality peak above 40% also had the highest cumulative excess mortality over the study period, suggesting a low overall health resilience. By contrast, five countries registered negative cumulative excess mortality. These countries were not affected by the first wave of the Covid-19 pandemic to the same extent as countries with high excess mortality. This was possibly due to greater geographical isolation, as in the case of Iceland, or a less important caseload before measures were implemented which has been found to be an important factor in the literature [19].

For the 10 countries for which significant values of both resistance and recovery could be calculated, the peak of excess mortality was reached in 4,8 weeks on average while the average recovery time was 7.7. Health resistance and recovery were negatively correlated: the lower the health resistance (higher peak), the faster the recovery ($r(10) = .97$, $p < .001$). Spain showed the lowest resistance (45.28%) with excess mortality rates increasing by 6.0-fold in the week of the 9$^{th}$ of March and then almost tripling to 158.25% within two weeks. Yet, the country also showed the highest recovery rate (-26.23%). Both low level of resistance ($r(10) = .86$, $p < .001$), and high level of recovery ($r(10) = .80$, $p < .01$), were associated with overall cumulative excess mortality. Spain, with the lowest resistance and highest recovery, also had the lowest expressed health system resilience (653.18%). Yet, low



For peer review only - http://bmjopen.bmj.com/site/about/guidelines.xhtml

15.06.2023

resistance does not necessarily imply low overall resilience. Switzerland exhibited a mortality peak and rather low resistance of 14.5% but with a cumulative excess mortality of only 108.57%, similar to other non-peaking countries like Austria (109.00%) or Greece (119.68%). This can be explained by the country's high recovery rate (-9.60%). With a resistance close to Switzerland's, Sweden (13.8%) had a lower recovery rate (-3.74%) as well as higher cumulative excess mortality (278.98%), compatible with the implementation of less stringent government interventions.

## 3.2 Resilience of fundamental rights and freedoms

Given the unprecedented level of restrictions implemented in all countries during the first wave of the COVID-19 pandemic in Europe, none can be considered as robust in terms of fundamental rights and freedoms. This is corroborated by further data which showed a further decline in democracy compared to previous years [20, 21]. As of 24 August 2020, no country had returned to its pre-pandemic situation. Italy, Portugal, United Kingdom, and Spain exhibited the highest cumulative stringency over the study period, while Sweden, Estonia, Iceland, and Finland exhibited the lowest cumulative stringency (supplementary material 4). The average cumulative fundamental rights and freedoms resilience for all 22 countries was 1478,72. Countries often maintained a high stringency level regardless of their epidemiological situation during the first wave, as reflected in the low fundamental rights and freedoms recovery average of -3.20 points compared to health average recovery rate of -9.36% (for countries with a statistically significant value for the calculation of health system recovery).

No relationship was found between resilience of fundamental rights and freedom and neither resistance ($r(22) = .21$, $p = .34$) or recovery ($r(22) = .16$, $p = .49$). Furthermore, no relationship was found between resistance and recovery of fundamental rights and freedoms ($r(22) = .31$, $p = .17$). In other words, low fundamental rights and freedoms resistance corresponded to a wide range of recovery rates. Various countries reacted strongly at first, suggesting weak resistance, but were then quick to remove their restrictions, such as Estonia which had the lowest resistance rate (the fastest escalation of strict measures), as well as a high recovery rate and the second-highest overall fundamental rights and freedoms resilience. This was reflected by positively skewed data, evident for France, Norway, Estonia, and the Czech Republic who adopted more stringent measures earlier on but were quick to remove them by the summer. In contrast, Sweden which recorded the highest cumulative resilience value showed a moderate resistance rate (9.10%) principally due to late measure introduction and a low recovery rate (-0.62%). Negatively skewed data was evident for Great Britain, Germany, Spain, Greece, and Sweden who imposed and maintained more stringent measures later in the period. Other countries escalated quickly and maintained stringent government interventions during the whole period, indicating a low recovery, such as Portugal or Denmark. These mixed results reflect the different strategies adopted by countries to deal with the COVID-19 during the first wave.

## 3.3 Economic system resilience

All countries experienced an economic decline, followed by recovery during the first wave, although to varying extents. These losses in turn translated in negative gross rate and a departure from the previous years. In this case, no country experienced similar values in the past five years suggesting that the robustness threshold was crossed for all 22 countries (supplementary material 5 and 6), even though governments usually supported their economy. The cumulative resilience average for all 22 countries is -225.78. For the 20 countries that had both significant values for economic resilience and recovery (all except Slovenia and Slovakia), economic decline took on average 14 weeks to reach the



For peer review only - http://bmjopen.bmj.com/site/about/guidelines.xhtml

economic trough. ’Economic resistance ($r(20) = .89$, $p < .001$) and recovery ($r(20) = .86$, $p < .001$) were found to be associated with overall economic resilience: lower resistance and faster recovery resulted in higher cumulative GDP losses. Countries with the highest resistance such as Denmark (-0.30%), Norway (-0.40%), Finland (-0.44%) also had some of the highest overall economic resilience. Economic resistance and economic recovery were negatively correlated meaning that lower resistance was associated with higher recovery rate ($r(20) = .84$, $p < .001$).

## 3.4 Multisystemic resilience

Cross-system comparisons of resilience results across the health, fundamental rights and freedoms, and economic systems allowed us to identify potential linkages and trade-offs between systems and thereby derive a multisystemic understanding of resilience. Only nine countries (Spain, United Kingdom, Italy, Belgium, Switzerland, Netherlands, Portugal, France, and Sweden) had a peak above 40% during the first wave. Although several countries were robust at the health system level, none of the 22 European countries were robust across all systems, as all 22 countries crossed economic and fundamental rights and freedoms robustness thresholds. This aligns with the idea that when resilience is provided by intervention (and not by design), there is transfer of capacities from other systems [22].

Health system resilience was associated with economic system resilience ($r(22) = .69$, $p < .001$); in other words, countries with higher excess mortality also suffered greater economic losses. Health system resilience appeared to be positively associated with overall fundamental rights and freedoms resilience but to a lesser extent ($r(22) = .56$, $p = .007$). This may be explained by the fact that many countries implemented stringent measures independently of their excess mortality trends. Finally, economic system resilience was found to be positively linked to fundamental rights and freedoms resilience ($r(22) = .67$, $p < .001$). In other words, the more restrictions on individual rights and freedoms, the more impact on the economy.

Some countries may exhibit a high level of resilience in one system but low resilience in another. When looking at the overall ranking based on countries’ resilience in each system (figure 2), four countries were in the first quartile – Norway, Iceland, Estonia, and Finland – and expressed high multisystemic resilience. On the other hand, Spain, the United Kingdom, Italy, Portugal, France and Belgium were in the third quartile and expressed lower multisystemic resilience. We further established three main categories of country during the first wave of the COVID-19 pandemic. Figure 3 provides a graph for Norway, Germany, and Spain to illustrate the three categories. It should be noted that these categories are not prescriptive nor rigid divisions, but rather constantly evolving trends as resilience is an adaptive capacity. There are indications that countries changed category as the pandemic evolves.

A group of six countries (Hungary, Iceland, Norway, Slovakia, Denmark, and Estonia) exhibited high health system resilience (except for Estonia, their excess mortality was negative). Estonia and Norway showed high level of resilience in all three systems, while Slovakia and Hungary had moderate resilience of fundamental rights and freedoms and economic resilience. Iceland adopted the second least stringent measures of the group yet also suffered moderate economic losses, potentially due to its greater dependence on the tourism industry [23]. By contrast, Denmark had a moderate resilience of fundamental rights and freedoms that was comparable to Hungary, yet better economic resilience.

A group of eight countries (Germany, Czechia, Slovenia, Finland, Poland, Austria, Switzerland, and Greece) exhibited moderate health system resilience comprised between 43.68% and 119.68% of


For peer review only - http://bmjopen.bmj.com/site/about/guidelines.xhtml

cumulative excess mortality. Except for Finland which demonstrated high resilience of fundamental rights and freedoms, all of them exhibited moderate resilience in this system. There was more variability regarding economic resilience with high resilience in Switzerland, Poland and Slovenia and moderate resilience in the rest of the group. In other words, this group of countries usually suffered more severe economic downturns resulting from both stricter governmental measures and moderate health resilience. The heavier economic losses may also be related to greater dependence on tourism, as in Greece, Austria, and Slovenia [23], as well as greater levels of economic dependence on foreign trade and exports to other European countries hit by less demand from abroad, as with Germany, the largest European exporter.

Finally, eight countries (France, Sweden, Portugal, Netherlands, Belgium, Italy, United Kingdom, and Spain) were part of a group with lower health system resilience with a cumulative excess mortality comprised between 251.33% to 653.18%. Five out of these eight countries were also countries with the lowest economic resilience. Except for Sweden, all countries exhibited either moderate (n=2) or low resilience (n=5) in fundamental rights and freedoms. Sweden's unique no-lockdown model resulted in the highest fundamental rights and freedoms resilience. By contrast, Italy, Portugal, and the UK proved to be the less resilient in terms of fundamental rights and freedoms due in part to weaker crisis preparedness and slow reaction in the UK and potentially the balance of power towards the executive for Portugal and Italy.

# 4 Discussion

## 4.1 Contribution to our understanding of resilience

This study operationalized the measurement of a multisystemic expression of resilience by assessing 22 European countries' resilience in three systems – health, economy, and fundamental rights and freedoms. Our approach helps understand how countries are affected and respond to systemic crises by managing different societal objectives between systems [24]. The comparative analysis shows a significant tendency among all 22 European countries to favor health and economic systems over fundamental rights and freedoms during the first wave of the COVID-19 pandemic in Europe in 2020. Furthermore, while the recovery time has been used by various authors as the main resilience indicator [4], our operationalization of robustness, resistance and recovery in addition to an overall measure of resilience provide a more granular understanding of how countries were impacted and reacted to the first wave of the COVID-19 pandemic. For example, when considering only the recovery rate, the country with the most resilience health system would also be the one with the lowest resistance and the lowest overall health system resilience in our dataset (Spain).

Our results provide further evidence that the COVID-19 pandemic tested not only the resilience of health systems but also economic resilience and the resilience of fundamental rights and freedoms. The resilience capacities of one system may depend on and impact the others. It is not surprising that the implementation of stringent containment measures negatively impacted a country's economic performance. Nevertheless, several countries enforced relatively strict measures while limiting economic consequences. In contrast, the effect of stringent government responses on health resilience is not as straightforward. Irrespective of their epidemiological situation, all governments adopted varying degrees of measures in early 2020, related to the novel character of the pandemic and precautionary principles. Except for Sweden, the 22 European countries prioritized their health and economic systems over fundamental rights and freedoms in the first wave. By the end of August,



For peer review only - http://bmjopen.bmj.com/site/about/guidelines.xhtml

15.06.2023

no government could restore pre-crisis governance operating modes. Lenton *et al.* found that prolonged stringent measures may not necessarily lead to improved health outcomes and may even reduce health system resilience in the long term due to decreased trust in government over time [4]. Finally, most countries in the dataset were more stringent than could be expected based on how liberal they usually are (supplementary material 7 and 8). Whether this led to a "pendulum policy effect" that ultimately delayed responses during the second wave should be investigated.

## 4.2 Limitations

The paper has some limitations regarding the conceptualization of resilience. One of the main limitations of assessing resilience across countries is the issue of comparability; countries were not equally exposed to COVID-19, and did not start with the same context, and therefore results showing high overall resilience do not necessarily imply the highest resilience capacity. In other words, our study measures in some ways how resilience capacities were expressed across different systems in relation to how they were exposed to the shock. Why and how it was expressed this way is another question that deserves more scrutiny. While stressing the multisystemic nature of resilience, we have limited our analysis to resilience in health, fundamental rights and freedoms and economic systems. These systems were selected because they were more easily discerned and measurable, but systemic effects generated by the COVID-19 crisis have also disrupted social, governance and environmental systems amongst others [1]. Furthermore, our approach explores only limited aspects of the multiple ways resilience can be expressed. The use of time series data highlights the capacity to cope and recover while overlooking the process of adaptation and transformation that is intrinsically important to the study of resilience [1].

The proxy Indicators used to measure health, fundamental rights and freedoms, and economic resilience have shortcomings. Regarding the GDP ratio-to-trend, the OECD stresses that it is more suitable for qualitative purposes to visualize the fluctuations of economic activity. Excess mortality was selected as it overcomes several issues related to the reporting of COVID-19 related deaths such as miscounting from misdiagnosis or under-reporting. However, it does not only include 'direct' COVID-19 mortality but also 'collateral damage' from other health conditions left untreated when the health system is overwhelmed. It also relies on the accuracy of mortality data from the five-year period before the crisis, which is more or less accurate from country to country. The OxCGRT's stringency index to measure the resilience of fundamental rights and freedoms reflects the breaches in civil liberties that are intrinsic to the values of liberal democracies. However, the indicator does not reflect the respect of democratic processes nor the rule of law, essential components to assess broader democratic resilience. Limitations also arise as this indicator is a composite of additive un-weighted indices that abstract away from nuances or any heterogeneity in the country responses. Its reliance on third party sources may also result in measurement bias [25]. Furthermore, unlike health and economic resilience, the stringency of measures does not simply reflect the 'country's inability to absorb and cope with the shock but also its ability to implement and modify measures quickly.

Additional limitations are related to measurements. First, resistance and recovery were estimated by using linear regression, but nonlinear regression functions might provide a better fit to the data. Second, our analysis does not account for resilience overshoot (for example, when the measures adopted result in a highly negative excess mortality). Third, we did not compute a unique multisystemic measure of resilience encompassing all systems as this would have required a normative judgment on whether one system should be considered more important than another. This



For peer review only - http://bmjopen.bmj.com/site/about/guidelines.xhtml

is fundamentally a political choice. While we recognize that directly comparing resilience in health, fundamental rights and freedom, and the economy can be challenging from a public health perspective, we believe that it is important to look at difficult questions of trade-offs. Such assessment can help foster a democratic debate about the values in our societies and help select where investment needs to be made to increase our capacities to prevent such shocks.

Finally, some reflections are needed about the scope and applicability of the methodological approach. Given the fact that the COVID-19 pandemic has not been a single event but recurring waves of different magnitude, we believe that our approach should be applied to study resilience to the different waves of the pandemic. This will lead to an understanding of whether and how countries adapt their priorities and strategies over time. Ideally, assessing resilience should be based on several indicators in each system. Given the effectiveness of immunization, one needs indicators that reflect the decoupling between mortality and infection. Another concern will be to expand the number of countries covered in the analysis. While the selected indicators are available for applicable to all countries, interpretations of the results should also consider the variety of cultural values and political system in the world. There also opportunities to use complementary methodological approaches such as interrupted times series to better relate what happened during the first wave of the COVID-19 pandemic and previous trends [26].

# 5 Conclusion

This study examines the multifaceted nature of resilience by investigating the interrelationships between health, fundamental rights and freedoms and economic systems. Our findings stress the limited pandemic preparedness capacities globally, as no country demonstrated robustness in all three systems. Countries with lower capacity to rapidly control the pandemic compensated by implementing stringent measures, often at the expense of individual rights and freedoms. Notably, economic resilience was found to be closely intertwined with health outcomes and the stringency of measures, highlighting the interdependence and significance of resilience across systems. Furthermore, the recovery process exhibited substantial variations across the three systems and among different countries. This underscores the complexity and diversity of recovery efforts, suggesting that interventions aimed at enhancing resilience necessitate trade-offs between systems with differing objectives. While further research is needed to deepen our understanding of multisystemic resilience, this study emphasizes the importance of resilience as a focal point for improving governance capacities to effectively prevent, respond to, and recover from current and future systemic shocks [24].

## 5.1 Ethics Statement

This study is based on online search of publicly available data. No patient, nor the public were involved in the design of the study. No ethical approval was sought as per our university requirements.

## 5.2 Contributors:

DW, MC and AR conceptualized the multisystemic measurement approach to resilience. MC and AR analysed the data and developed the successive drafts of the paper. MC, AR and DW designed the figures, tables and supplementary material. MC, AR, FT, CM, YK, NL and DW contributed content and comments to the paper. DW and NL are both principal investigators within the COVID-19 systemic crisis project.



For peer review only - http://bmjopen.bmj.com/site/about/guidelines.xhtml

15.06.2023

## 5.3 Competing Interests:

None declared.


## 5.4 Funding:

This research was funded by the Swiss National Science Foundation (SNSF), within the scope of the COVID-19 systemic crisis project (Grant 31CA30_196396, https://data.snf.ch/covid-19/snsf/196396)


## 5.5 Data Sharing Statement

All data used for this study are available in a public, open access repository. The sources of the data are the following 1) health resilience: the Human Mortality Database [Reference 16], 2) resilience of fundamental rights and freedoms: Oxford COVID-19 Government Response Tracker, Blavatnik School of Government, University of Oxford (CC BY License) [Reference 17], 3) economic resilience: OECD Main Economic Indicators Publication [Reference 18].

For peer review only

15.06.2023

# 6 References


1. Wernli, D., et al., *Building a multisystemic understanding of societal resilience to the COVID-19 pandemic.* BMJ Glob Health, 2021. **6**(7): p. e006794.
2. Linkov, I., et al., *Changing the resilience paradigm.* Nature Climate Change, 2014. **4**(6): p. 407-409.
3. Wernli, D., et al., *Governance in the age of complexity: building resilience to COVID-19 and future pandemics*. 2021, Geneva Science Policy Interface, University of Geneva, Switzerland.
4. Lenton, T.M., C.A. Boulton, and M. Scheffer, *Resilience of countries to COVID-19 correlated with trust.* Scientific Reports, 2022. **12**(1): p. 75.
5. Haldane, V., et al., *Health systems resilience in managing the COVID-19 pandemic: lessons from 28 countries.* Nature Medicine, 2021.
6. Arsenault, C., et al., *COVID-19 and resilience of healthcare systems in ten countries.* Nature Medicine, 2022.
7. Saulnier, D.D., et al., *A health systems resilience research agenda: moving from concept to practice.* BMJ Global Health, 2021. **6**(8): p. e006779.
8. Helbing, D., *Globally networked risks and how to respond.* Nature, 2013. **497**(7447): p. 51-59.
9. Ungar, M., *Multisystemic resilience: Adaptation and transformation in contexts of change*. 2021: Oxford University Press, USA.
10. Folke, C., et al., *Resilience thinking: integrating resilience, adaptability and transformability.* Ecol. Soc., 2010. **15**: p. 20.
11. Pimm, S.L., et al., *Measuring resilience is essential to understand it.* Nature Sustainability, 2019. **2**(10): p. 895-897.
12. Grafton, R.Q., et al., *Realizing resilience for decision-making.* Nature Sustainability, 2019. **2**(10): p. 907-913.
13. Galaitsi, S.E., et al., *The Need to Reconcile Concepts that Characterize Systems Facing Threats.* Risk Analysis, 2021. **41**(1): p. 3-15.
14. Schiller, C., et al., *Just How Resilient are OECD and EU Countries? Sustainable Governance in the Context of the COVID-19 Crisis*. 2021, Bertelsmann Stiftung.
15. von Elm, E., et al., *Strengthening the Reporting of Observational Studies in Epidemiology (STROBE) statement: guidelines for reporting observational studies.* Bmj, 2007. **335**(7624): p. 806-8.

[dataset]16. *Human Mortality Database [Internet]*. [cited 2021 16 August]; Available from: https://www.mortality.org/.

[dataset] 17. *COVID-19 Government Response Tracker [Internet]*. [cited 2021 16 August]; Available from: https://www.bsg.ox.ac.uk/research/research-projects/covid-19-government-response-tracker.

[dataset]18. *Main Economic Indicators Publication [Internet]*. [cited 2021 16 August]; Available from: https://stats.oecd.org/Index.aspx?DataSetCode=MEI.

19. Haug, N., et al., *Ranking the effectiveness of worldwide COVID-19 government interventions.* Nature Human Behaviour, 2020.
20. Youngs, R., *COVID-19 and democratic resilience.* Glob Policy, 2022.
21. Lewkowicz, J., M. Woźniak, and M. Wrzesiński, *COVID-19 and erosion of democracy.* Econ Model, 2022. **106**: p. 105682.
22. Hynes, W., et al., *Systemic resilience in economics.* Nature Physics, 2022. **18**(4): p. 381-384.
23. OECD, *OECD Tourism Trends and Policies 2020*. 2020.
24. Wernli, D., et al., *Understanding and governing global systemic crises in the 21st century: A complexity perspective.* Global Policy, 2023. **n/a**(n/a).
25. Hale, T., et al., *Government responses and COVID-19 deaths: Global evidence across multiple pandemic waves.* PLOS ONE, 2021. **16**(7): p. e0253116.




For peer review only - http://bmjopen.bmj.com/site/about/guidelines.xhtml

15.06.2023

26. Wambua, S., et al., *The indirect impact of COVID-19 pandemic on inpatient admissions in 204 Kenyan hospitals: An interrupted time series analysis.* PLOS Global Public Health, 2021. **1**(11): p. e0000029.

Figure legends/caption

Figure 1 Conceptual framework of resilience and its sub-components: robustness, resistance, and recovery inspired by Grafton et al. [12]

Figure 2 Comparison of resilience of health, fundamental rights and freedoms, and economic systems

Figure 3 Time series of selected countries representing the 3 categories and combining resilience measurements of the health, fundamental rights and freedoms, and economic systems

For peer review only

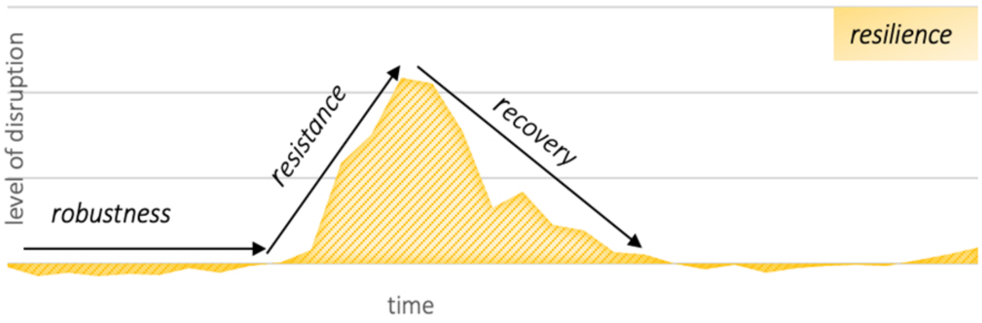


Conceptual framework of resilience and its sub-components: robustness, resistance, and recovery inspired by Grafton et al. [11]

402x139mm (57 x 57 DPI)

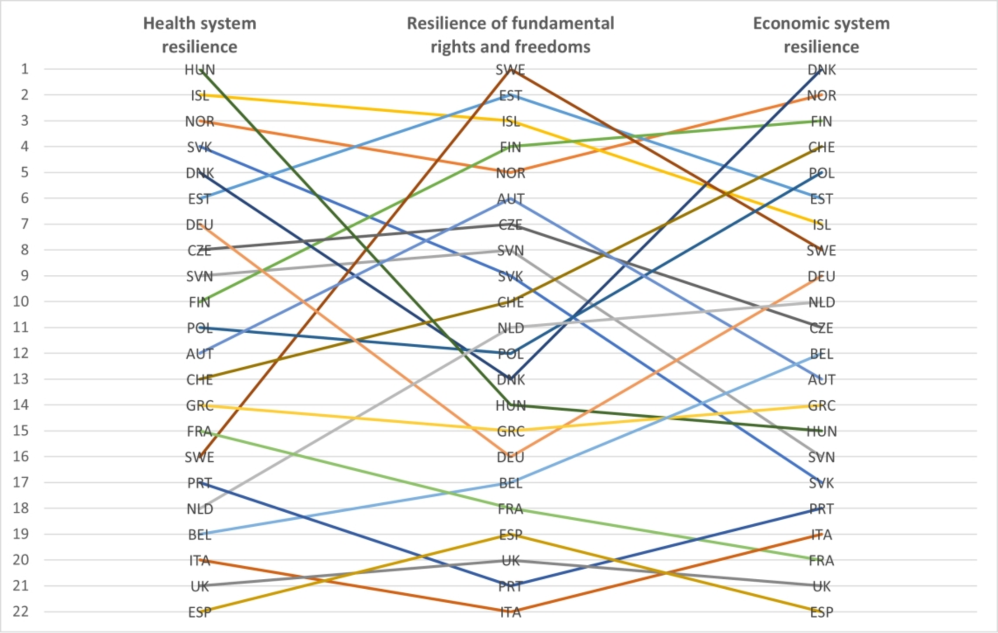


Figure 2 Comparison of resilience of health, fundamental rights and freedoms, and economic systems

488x308mm (130 x 130 DPI)

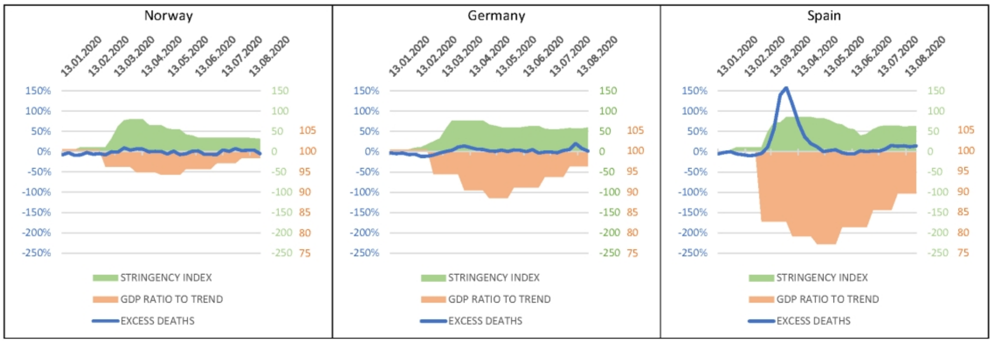


Time series of selected countries representing the 3 categories and combining resilience measurements of the health, fundamental rights and freedoms, and economic systems

697x243mm (79 x 79 DPI)

15.06.2023

# Multisystemic resilience to shocks: a temporal analysis of health, fundamental rights and freedoms, and economic resilience during the first wave of COVID-19's in 22 European countries

Mia Clausin, Alicia Rieckhoff, Fabrizio Tediosi, Chantal Morel, Yuliya Kaspiarovich, Nicolas Levrat, Didier Wernli

## Supplementary material

**Supplementary material 1 – Method used to derive metrics about multisystemic resilience**

We calculated several Z-scores to compare the overall balance in terms of resilience in different systems. Regarding excess mortality (1), the Z-score for 2020 was calculated as the value for 2020 (x) minus the mean for the period 2015 – 2020 (μ). The difference was then divided by the standard deviation (σ). Regarding stringency (2), we calculated a Z-score for the 2020 period to provide information about the stringency of a country's response compared to other countries. Finally, a Z-score was calculated for the Liberal Democracy Index (3) from V-Dem (https://v-dem.net/) from 2015 to 2020 to evaluate how liberal countries were before the pandemic.

Two different multisystemic resilience metrics were derived from these three Z-scores. To avoid computing metrics with negative Z-score values, the lowest value of each Z-score was set to 0 and all scores were modified accordingly. For example, regarding excess mortality, the lowest excess mortality of -1.350 for Iceland was set to zero. For the liberal democracy score, we also inverted the sign of each value meaning that a low z-score corresponds to a more liberal democracy than a high score and then put the lowest value to zero (Denmark and Sweden). The numbers are reported in supplementary material 4.

The first metric is the *difference between z-scores for stringency of the response and for excess mortality* which gives an indication of the adequation between the response and the problem based on a comparison between countries during the first wave of the COVID-19 pandemic in Europe. A negative score suggests that overall, the response may have been insufficient compared to the magnitude of the problem while a highly positive score suggests that the response may have been overtly stringent to the magnitude of the problem experienced by the country. It relies on the assumption that a higher stringency index is politically more justifiable in case of higher excess mortality (it must however be noted that the precautionary principle may impose to take some measures to protect public health early on).

The second metric is the *difference between z-scores for the state of (il)liberal democracy and for the stringency of the response* which gives an idea about the departure from the traditional way of governing during the first wave of the COVID-19 pandemic in Europe in early 2020. This score assumes that a liberal country may be more likely to protect fundamental rights and freedoms even in times of emergency. A negative score suggests that a country that is traditionally liberal was more stringent than one would have expected to be compared to other European countries during the first wave of the COVID-19. Supplementary material 5 shows a plot of the two metrics against each other.



For peer review only - http://bmjopen.bmj.com/site/about/guidelines.xhtml

15.06.2023

**Supplementary material 2 – Box plots and whisker for robustness in health systems in 22 countries based on excess mortality during week 3 to 35 for the years 2015 – 2020.**

We used the 1.5 interquartile range method to detect outliers (lower bound: (Q1 - 1.5 * IQR) and upper bound: (Q3 + 1.5 * IQR). The outlier value of excess mortality observed for Denmark and Germany was not in 2020. This method for the detection of outliers has an important limitation as it depends on the variability of excess mortality in the previous years. For example, the Netherlands had an excess mortality of 10,05% in 2020 but this value was not an outlier according to the 1.5 IQR rule due to variability of excess mortality during the 2015 – 2020 period.

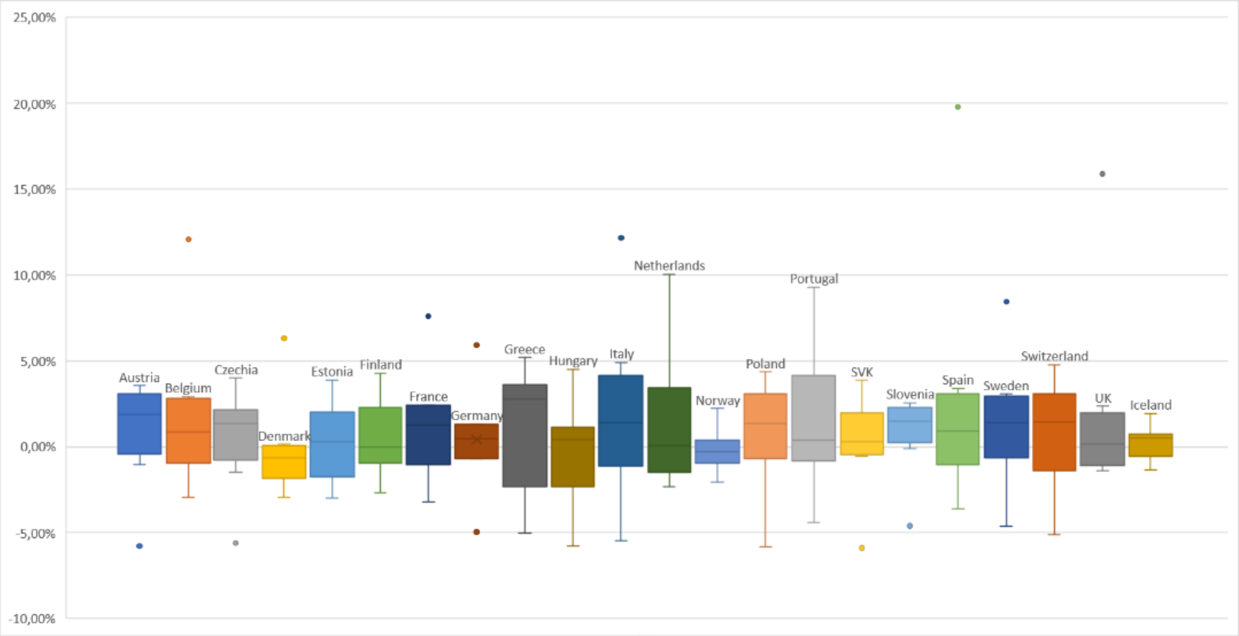


**Supplementary material 3 - Health system resilience, resistance, recovery and robustness**

| Countries | Resilience (cumulative excess mortality) | Resistance rate | Recovery rate | Robustness |
|---|---|---|---|---|
| Austria | 109.00% | 3.16% (>0.05) | -4.86% (<0.01) | Yes |
| Belgium | 398.35% | 29.89% (<0.01) | -14.67% (<0.01) | No |
| Czechia | 45.39% | 2.84% (<0.05) | -2.23% (>0.05) | Yes |
| Germany | 43.68% | 4,96% (<0.05) | -1.73% (<0.01) | Yes |
| Denmark | -3.49% | 5.33% (n/a) | -9.18% (n/a) | Yes |
| Spain | 653.18% | 45.28% (<0.05) | -26.23% (<0.001) | No |
| Estonia | 24.27% | 4,94% (>0.05) | -5,37 (>0.05) | Yes |
| Finland | 99.68% | 2,5% (>0.05) | -9.34% (n/a) | Yes |
| France | 251.33% | 17.29% (<0.01) | -8.30% (<0.01) | No |
| Greece | 119.68% | 7.99% (>0.05) | -8.22% (>0.05) | Yes |
| Hungary | -100.28% | 10.23% (n/a) | -6.22 (n/a) | Yes |
| Iceland | -43.83% | 9.45% (>0.05) | 44.63% (n/a) | Yes |
| Italy | 401.12% | 23.68% (<0.01) | -10.77% (<0.001) | No |
| Netherlands | 331.56% | 20.52% (<0.01) | -10.73% (<0.001) | Yes |
| Norway | -37.99% | 10,30% (n/a) | -5,37% (n/a) | Yes |
| Poland | 103.42% | 2.47% (>0.05) | -11.68% (n/a) | Yes |
| Portugal | 307.00% | 5.11% (<0,01) | -3.35% (<0.01) | Yes |
| Slovakia | -7.74% | 5.64% (>0.05) | -9.68% (n/a) | Yes |
| Slovenia | 84.56% | 19.55% (n/a) | -5.69% (<0.01) | Yes |
| Sweden | 278.98% | 13.80% (<0.05) | -3.74% (<0.001) | No |
| Switzerland | 108.57% | 14.50% (<0.01) | -9.60% (<0.001) | Yes |
| United Kingdom | 523.97% | 28.23% (<0.01) | -12.63% (<0.001) | No |



For peer review only - http://bmjopen.bmj.com/site/about/guidelines.xhtml

15.06.2023

**Supplementary material 4 – Resilience, resistance, recovery and robustness of fundamental rights and freedoms**

| Countries | Cumulative resilience | Resistance | Recovery | Robustness |
|---|---|---|---|---|
| Austria | 1346,44 | 19.47 (<0.05) | -3.75 (<0.001) | No |
| Belgium | 1665,90 | 7.68 (<0.01) | -2.54 (<0.001) | No |
| Czechia | 1356,05 | 7.51 (<0.001) | -3.51 (<0.001) | No |
| Germany | 1638,86 | 6.61 (<0.001) | -1.05 (<0.01) | No |
| Denmark | 1531,12 | 16.53 (<0.001) | -1.17 (<0.001) | No |
| Spain | 1748,22 | 8.64 (<0.001) | -5.42 (<0.05) | No |
| Estonia | 1082,11 | 20.24 (<0.05) | -4.14 (<0.001) | No |
| Finland | 1234,17 | 6.54 (<0.001) | -2.51 (<0.001) | No |
| France | 1694,83 | 8.52 (<0.001) | -4.98 (<0.001) | No |
| Greece | 1590,63 | 17.06 (<0.001) | -4.59 (<0.001) | No |
| Hungary | 1533,83 | 15.08 (<0.001) | -1.33 (<0.001) | No |
| Iceland | 1137,53 | 4.25 (<.001) | -2.79 (0.001) | No |
| Italy | 1899,23 | 8.65 (<0.001) | -5.96 (<0.05) | No |
| Netherlands | 1478,14 | 15.59 (<0.01) | -3.60 (<0.001) | No |
| Norway | 1286,62 | 8.02 (<0.001) | -2.22 (<0.001) | No |
| Poland | 1517,30 | 7.31 (<0.001) | -4.63 (<0.01) | No |
| Portugal | 1842,75 | 7.66 (<0.001) | -1.80 (<0.001) | No |
| Slovakia | 1396,71 | 9.03 (<0.001) | -2.87 (<0.001) | No |
| Slovenia | 1370,70 | 19.91 (<0.001) | -6.47 (<0.001) | No |
| Sweden | 991,859 | 9.10 (<0.001) | -0.62 (<0.01) | No |
| Switzerland | 1417,79 | 15.54 (<0.01) | -3.64 (<0.001) | No |
| United Kingdom | 1771,10 | 6.09 (<0.01) | -0.76 (<0.05) | No |

**Supplementary material 5 – Box plots and whisker for robustness in economic system in 22 European countries based on GDP growth (annual %) from 2015 – 2020**

Data were collected from https://data.worldbank.org/indicator/NY.GDP.MKTP.KD.ZG. The method used for the detection of outliers is the same as for supplementary material 2. All countries had an outlier value for 2020 based on the 1,5 interquartile range method.

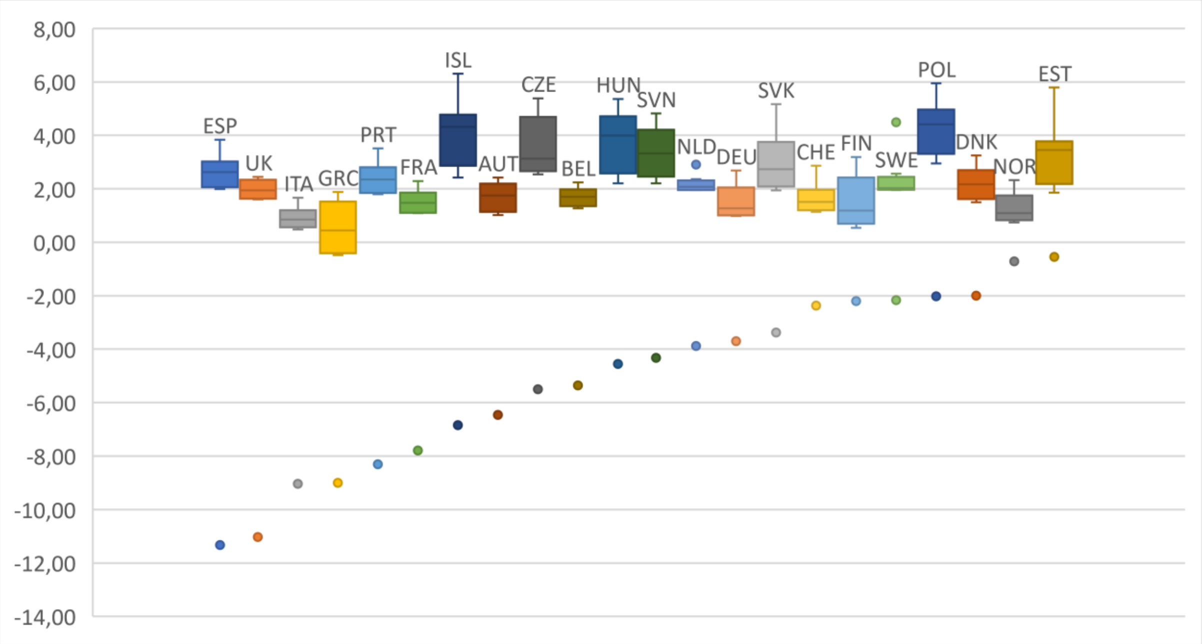


Abbreviations: Austria AUT, Belgium BEL, Czechia CZE, Denmark DNK, Estonia EST, Finland FIN, France FRA, Germany DEU, Greece GRC, Hungary HUN, Iceland ISL, Italy ITA, Netherlands NLD, Norway NOR, Poland POL, Portugal PRT, Slovak Republic SVK, Slovenia SVN, Spain ESP, Sweden SWE, Switzerland CHE, United Kingdom UK.

15.06.2023

## Supplementary material 6 - Economic system resilience, resistance, recovery and robustness

| Economic system (points/week) | | | | |
|---|---|---|---|---|
| Countries | Cumulative resilience | Resistance rate | Recovery rate | Robustness |
| Austria | -223,93 | -0.84 (<0.001) | 0.69 (<0.001) | No |
| Belgium | -221,94 | -0.91 (<0.001) | 0.82 (<0.001) | No |
| Czechia | -217,82 | -1.15 (<0.001) | 0.43 (<0.001) | No |
| Germany | -189,65 | -0.94 (<0.001) | 0.59 (<0.001) | No |
| Denmark | -93,66 | -0.30 (<0.001) | 0.27 (<0.001) | No |
| Spain | -450,65 | -1.37 (<0.01) | 0.94 (<0.001) | No |
| Estonia | -144,06 | -0.61 (<0.001) | 0.26 (<0.001) | No |
| Finland | -105,27 | -0.44 (<0.001) | 0.29 (<0.001) | No |
| France | -342,62 | -1.64 (<0.001) | 1.13 (<0.001) | No |
| Greece | -246,33 | -1.00 (<0.001) | 0.30 (<0.001) | No |
| Hungary | -251,55 | -1.51 (<0.001) | 0.66 (<0.001) | No |
| Iceland | -153,08 | -0.76 (<0.001) | 0.33 (<0.001) | No |
| Italy | -341,61 | -1.57 (<0.001) | 0.98 (<0.001) | No |
| Netherlands | -205,86 | -0.85 (<0.001) | 0.46 (<0.001) | No |
| Norway | -95,82 | -0.40 (<0.01) | 0.32 (<0.001) | No |
| Poland | -127,16 | -0.76 (<0.001) | 0.47 (<0.001) | No |
| Portugal | -325,42 | -1.21 (<0.05) | 0.89 (<0.001) | No |
| Slovakia | -284,89 | -13.76 (n/a) | 0.37 (<0.001) | No |
| Slovenia | -269,03 | -17.17 (n/a) | 0.55 (<0.001) | No |
| Sweden | -172,21 | -0.71 (<0.001) | 0.44 (<0.001) | No |
| Switzerland | -116,29 | -0.68 (<0.001) | 0.44 (<0.001) | No |
| United Kingdom | -388,33 | -1.68 (<0.001) | 1.01 (<0.001) | No |

For peer review only

15.06.2023

**Supplementary material 7 – Table of Z-scores for liberal democracy index, stringency of the response, and excess mortality and two metrics based on these three Z-scores**

The methodology used to produce this table is described in supplementary material 1.

| Country | Excess-mortality | Stringency index | Liberal democracy index | Difference between stringency index and excess mortality | Difference between liberal democracy and stringency index |
|---|---|---|---|---|---|
| Austria | 2.097 | 1.449 | 1.086 | -0.648 | -0.363 |
| Belgium | 3.205 | 2.755 | 0.545 | -0.450 | -2.210 |
| Czechia | 1.654 | 1.489 | 1.426 | -0.166 | -0.062 |
| Denmark | 1.307 | 2.204 | 0.000 | 0.898 | -2.204 |
| Estonia | 1.550 | 0.369 | 0.404 | -1.181 | 0.035 |
| Finland | 2.292 | 0.991 | 0.559 | -1.302 | -0.432 |
| France | 3.013 | 2.874 | 0.712 | -0.140 | -2.161 |
| Germany | 1.582 | 2.645 | 0.471 | 1.063 | -2.173 |
| Greece | 1.990 | 2.448 | 1.196 | 0.458 | -1.252 |
| Hungary | 0.610 | 2.215 | 4.526 | 1.606 | 2.310 |
| Iceland | 0.000 | 0.595 | 0.967 | 0.595 | 0.371 |
| Italy | 3.012 | 3.709 | 0.899 | 0.697 | -2.810 |
| Netherlands | 3.117 | 1.988 | 0.567 | -1.129 | -1.421 |
| Norway | 0.674 | 1.205 | 0.280 | 0.531 | -0.924 |
| Poland | 2.026 | 2.148 | 2.815 | 0.122 | 0.667 |
| Portugal | 2.921 | 3.478 | 0.426 | 0.557 | -3.053 |
| Slovak Republic | 1.260 | 1.655 | 1.615 | 0.395 | -0.040 |
| Slovenia | 2.091 | 1.549 | 1.060 | -0.543 | -0.488 |
| Spain | 3.301 | 3.092 | 1.090 | -0.209 | -2.002 |
| Sweden | 2.940 | 0.000 | 0.000 | -2.940 | 0.000 |
| Switzerland | 2.073 | 1.741 | 0.306 | -0.332 | -1.435 |
| United Kingdom | 3.343 | 3.185 | 0.883 | -0.158 | -2.302 |



For peer review only - http://bmjopen.bmj.com/site/about/guidelines.xhtml

15.06.2023

**Supplementary material 8 – Difference between liberal democracy and stringency index (y axis) as a function of the difference between stringency index and excess mortality (x axis) for 22 European countries during the first wave of the COVID-19 pandemic in early 2020.**

The methodology used to produce this figure is covered in supplementary materials 1 and 4.

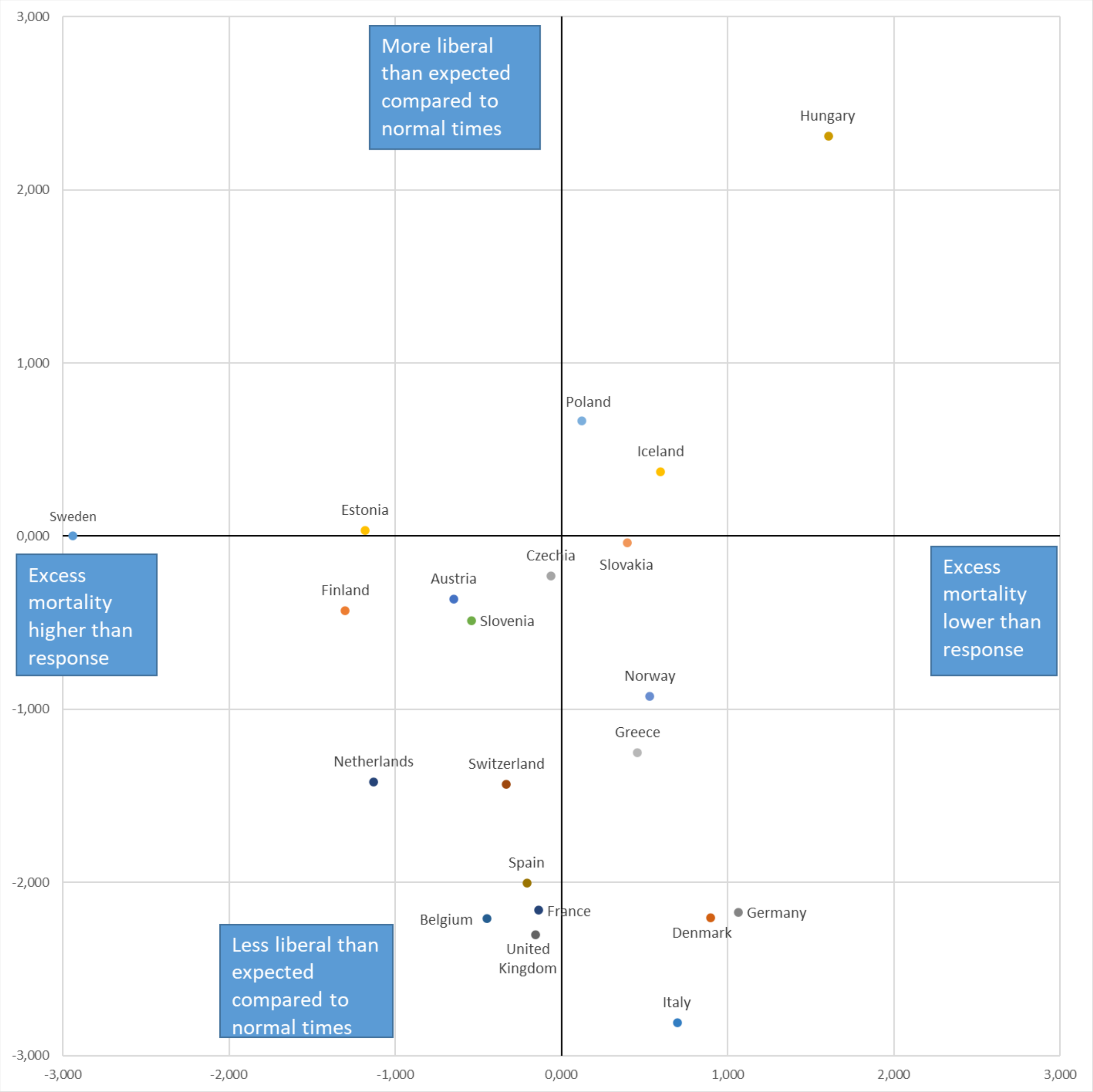

STROBE Statement—checklist of items that should be included in reports of observational studies

| | Item No. | Recommendation | Page No. | Relevant text from manuscript |
|---|---|---|---|---|
| **Title and abstract** | 1 | (*a*) Indicate the study's design with a commonly used term in the title or the abstract | 1 | Temporal analysis |
| | | (*b*) Provide in the abstract an informative and balanced summary of what was done and what was found | 1 | This study uses time series data to assess resilience in health, fundamental right and freedoms and economic systems. An overall resilience was estimated, as well as three of its sub-components: robustness, resistance, and recovery. |
| **Introduction** | | | | |
| Background/rationale | 2 | Explain the scientific background and rationale for the investigation being reported | 2 | While current research on resilience to COVID-19 primarily focuses on health systems, [4-6], there is a need to assess resilience beyond health systems [7]. Considering resilience as a multi-objective societal problem, the concepts of 'systemic resilience' [8] and 'multisystemic resilience' [9] have emerged to better understand the impact of shocks across highly interconnected systems. |
| Objectives | 3 | State specific objectives, including any prespecified hypotheses | 2 | this paper aims to provide a practical application of how to measure multisystemic resilience and its dynamics over the first wave of the COVID-19 pandemic by assessing three components of resilience – robustness, resistance, and recovery [12]. |
| **Methods** | | | | |
| Study design | 4 | Present key elements of study design early in the paper | 2 | This is a cross-country study depicting the first wave of the COVID-19 pandemic in 22 European countries measuring health resilience, economic resilience and resilience of fundamental rights and freedoms. This study provides insights about how different systems are affected by the COVID-19 pandemic. |
| Setting | 5 | Describe the setting, locations, and relevant dates, including periods of recruitment, exposure, follow-up, and data collection | 3 | This study investigates the temporal dynamics of resilience of three systems, i.e., health, individual rights and freedoms, and the economy, during the initial wave of the COVID-19 pandemic in Europe. An extensive review of academic and intergovernmental sources was conducted to identify relevant indicators of resilience. This search encompassed sources such as the World Bank, World Health Organization, OECD, and European Union. Indicators were selected based on two key criteria: availability of weekly or monthly data capturing temporal evolution, and relevance to the specific type of resilience being examined (see the measurements |

For peer review only

| | | | | |
|---|---|---|---|---|
| | | | | section below). Data collection spanned from week 3, before the implementation of any COVID-19 measures, to week 35, when COVID-19 deaths approached their lowest levels across European countries (January 13, 2020, to August 31, 2020). The analysis focuses on twenty-two European countries chosen based on the availability of data pertaining to the three selected systems. Countries lacking data for any of the three systems at the time of data collection were excluded from the analysis. The collected data for each indicator was systematically compiled in separate spreadsheets for each country. |
| Participants | 6 | (*a*) *Cohort study*—Give the eligibility criteria, and the sources and methods of selection of participants. Describe methods of follow-up<br>*Case-control study*—Give the eligibility criteria, and the sources and methods of case ascertainment and control selection. Give the rationale for the choice of cases and controls<br>*Cross-sectional study*—Give the eligibility criteria, and the sources and methods of selection of participants | NA | NA |
| | | (*b*) *Cohort study*—For matched studies, give matching criteria and number of exposed and unexposed<br>*Case-control study*—For matched studies, give matching criteria and the number of controls per case | NA | NA |
| Variables | 7 | Clearly define all outcomes, exposures, predictors, potential confounders, and effect modifiers. Give diagnostic criteria, if applicable | 4-5 | Table 1 outlines the definitions used for resilience and its 3 components across the different systems and Figure 1 provides a visual representation of these concepts. Table 2 outlines the criteria used to measure health, fundamental rights and freedoms, and economic resilience, robustness, resistance, and recovery. Overall resilience in health, fundamental rights and freedoms and economic systems was estimated using the cumulative value for the selected measurements. + section 2.4 |
| Data sources/ | 8* | For each variable of interest, give sources of | 3 | Section 2.3 of the manuscript. For example regarding excess mortality: The indicator |


For peer review only - http://bmjopen.bmj.com/site/about/guidelines.xhtml

| | | | | |
|---|---|---|---|---|
| measurement | | data and details of methods of assessment (measurement). Describe comparability of assessment methods if there is more than one group | | selected for measuring health system resilience was weekly excess mortality, from the Human Mortality Database [16]. A death is recorded by week of occurrence, apart from the UK, where death by week of registration is used. Excess mortality was selected as it helps overcome several issues related to the reporting of COVID-19 related deaths such as miscounting from misdiagnosis or under-reporting. It also includes 'collateral damage' from other health conditions left untreated if the health system is overwhelmed [6]. Excess mortality was defined as the number of deaths registered in excess of the five-year average from 2015 to 2019. The calculations for Germany and Greece were based on the four-year average (2016-2019) due to unavailable data for 2015. A weekly P-score, which was chosen as it allows for robust country comparisons, was calculated as the percentage difference between the reported and projected number of deaths. Health system resilience was interpreted as the inverse of excess mortality. The lower the excess mortality, the larger health system resilience. |
| Bias | 9 | Describe any efforts to address potential sources of bias | 4 + 11-12 | Results were then used to conduct a comparative analysis across systems and identify general trends and potential trade-offs between them. This allowed us to analyse the resilience level relative to other systems' results and minimise bias caused by different time exposure to the virus from one country population to another. |
| Study size | 10 | Explain how the study size was arrived at | 3 | The analysis focuses on twenty-two European countries chosen based on the availability of data pertaining to the three selected systems. Countries lacking data for any of the three systems at the time of data collection were excluded from the analysis. The collected data for each indicator was systematically compiled in separate spreadsheets for each country. |

Continued on next page


For peer review only - http://bmjopen.bmj.com/site/about/guidelines.xhtml

| | | | | |
|---|---|---|---|---|
| Quantitative variables | 11 | Explain how quantitative variables were handled in the analyses. If applicable, describe which groupings were chosen and why | 3 | The collected data for each indicator was systematically compiled in separate spreadsheets for each country. |
| Statistical methods | 12 | (*a*) Describe all statistical methods, including those used to control for confounding | 6 | We conducted an evaluation of association among the measurements of resilience across multiple systems. Firstly, we calculated Pearson correlation coefficients for each system to examine the potential correlation between resistance, recovery, and overall resilience. While we successfully computed an overall resilience measure for each country across the three systems, certain combinations of systems and countries did not yield statistically significant results ($p > .05$) regarding the correlation between resistance, recovery, and overall resilience. Consequently, these countries were excluded from the calculation of associations between resilience and resistance/recovery within the specific system. Secondly, we calculated Pearson correlation coefficients to assess whether overall resilience within one system exhibited correlations with resilience in other systems. To facilitate further comparison of resilience across the different systems, we utilized z-scores to derive two additional metrics. The first metric quantifies the disparity between excess mortality and response stringency, providing an indication of the appropriateness of the response relative to the magnitude of the problem. The second metric measures the deviation from the conventional approach to governance by assessing the difference between an index measuring the state of liberal democracy and the stringency index. The methodology employed for deriving these metrics can be found in supplementary material 1. |
| | | (*b*) Describe any methods used to examine subgroups and interactions | 6 | CF text above about the mesure of association |
| | | (*c*) Explain how missing data were addressed | NA | NA |
| | | (*d*) *Cohort study*—If applicable, explain how loss to follow-up was addressed<br>*Case-control study*—If applicable, explain how matching of cases and controls was addressed<br>*Cross-sectional study*—If applicable, describe analytical methods taking account of sampling | NA | NA |



For peer review only - http://bmjopen.bmj.com/site/about/guidelines.xhtml

| | | | | |
|---|---|---|---|---|
| | | strategy | | |
| | | (*e*) Describe any sensitivity analyses | NA | NA |
| **Results** | | | | |
| Participants | 13* | (a) Report numbers of individuals at each stage of study—eg numbers potentially eligible, examined for eligibility, confirmed eligible, included in the study, completing follow-up, and analysed | NA | NA |
| | | (b) Give reasons for non-participation at each stage | NA | NA |
| | | (c) Consider use of a flow diagram | NA | NA |
| Descriptive data | 14* | (a) Give characteristics of study participants (eg demographic, clinical, social) and information on exposures and potential confounders | NA | NA |
| | | (b) Indicate number of participants with missing data for each variable of interest | NA | NA |
| | | (c) *Cohort study*—Summarise follow-up time (eg, average and total amount) | NA | NA |
| Outcome data | 15* | *Cohort study*—Report numbers of outcome events or summary measures over time | NA | NA |
| | | *Case-control study*—Report numbers in each exposure category, or summary measures of exposure | NA | NA |
| | | *Cross-sectional study*—Report numbers of outcome events or summary measures | NA | NA |
| Main results | 16 | (*a*) Give unadjusted estimates and, if applicable, confounder-adjusted estimates and their precision (eg, 95% confidence interval). Make clear which confounders were adjusted for and why they were included | NA | NA |
| | | (*b*) Report category boundaries when continuous variables were categorized | NA | NA |
| | | (*c*) If relevant, consider translating estimates of | NA | NA |



For peer review only - http://bmjopen.bmj.com/site/about/guidelines.xhtml

| | | | | |
|---|---|---|---|---|
| | | relative risk into absolute risk for a meaningful time period | | |

Continued on next page

For peer review only



For peer review only - http://bmjopen.bmj.com/site/about/guidelines.xhtml

| | | | | |
|---|---|---|---|---|
| Other analyses | 17 | Report other analyses done—eg analyses of subgroups and interactions, and sensitivity analyses | NA | NA |
| **Discussion** | | | | |
| Key results | 18 | Summarise key results with reference to study objectives | P.12 | This study examines the multifaceted nature of resilience by investigating the interrelationships between health, fundamental rights and freedoms and economic systems. Our findings stress the limited pandemic preparedness capacities globally, as no country demonstrated robustness in all three systems. Countries with lower capacity to rapidly control the pandemic compensated by implementing stringent measures, often at the expense of individual rights and freedoms. Notably, economic resilience was found to be closely intertwined with health outcomes and the stringency of measures, highlighting the interdependence and significance of resilience across systems. |
| Limitations | 19 | Discuss limitations of the study, taking into account sources of potential bias or imprecision. Discuss both direction and magnitude of any potential bias | p.11-12 | The whole section is dedicated to these limitations |
| Interpretation | 20 | Give a cautious overall interpretation of results considering objectives, limitations, multiplicity of analyses, results from similar studies, and other relevant evidence | p.11 (discussion)<br><br>p.12 (conclusion) | Our results provide further evidence that the COVID-19 pandemic tested not only the resilience of health systems but also economic resilience and the resilience of fundamental rights and freedoms. The resilience capacities of one system may depend on and impact the others.<br><br>Our findings stress the limited pandemic preparedness capacities globally, as no country demonstrated robustness in all three systems. Countries with lower capacity to rapidly control the pandemic compensated by implementing stringent measures, often at the expense of individual rights and freedoms. Notably, economic resilience was found to be closely intertwined with health outcomes and the stringency of measures, highlighting the interdependence and significance of resilience across systems. Furthermore, the recovery process exhibited substantial variations across the three systems and among different countries. This underscores the complexity and diversity of recovery efforts, suggesting that interventions aimed at enhancing resilience necessitate trade-offs between systems with differing objectives. While |


For peer review only

| | | | | |
|---|---|---|---|---|
| | | | | further research is needed to deepen our understanding of multisystemic resilience, this study emphasizes the importance of resilience as a focal point for improving governance capacities to effectively prevent, respond to, and recover from current and future systemic shocks [24]. |
| Generalisability | 21 | Discuss the generalisability (external validity) of the study results | p.12 | Finally, some reflections are needed about the scope and applicability of the methodological approach. Given the fact that the COVID-19 pandemic has not been a single event but recurring waves of different magnitude, we believe that our approach should be applied to study resilience to the different waves of the pandemic. This will lead to an understanding of whether and how countries adapt their priorities and strategies over time. Ideally, assessing resilience should be based on several indicators in each system. Given the effectiveness of immunization, one needs indicators that reflect the decoupling between mortality and infection. Another concern will be to expand the number of countries covered in the analysis. While the selected indicators are available for applicable to all countries, interpretations of the results should also consider the variety of cultural values and political system in the world. There also opportunities to use complementary methodological approaches such as interrupted times series to better relate what happened during the first wave of the COVID-19 pandemic and previous trends [26]. |
| **Other information** | | | | |
| Funding | 22 | Give the source of funding and the role of the funders for the present study and, if applicable, for the original study on which the present article is based | p.13 | This research was funded by the Swiss National Science Foundation (SNSF), within the scope of the COVID-19 systemic crisis project (Grant 31CA30_196396, https://data.snf.ch/covid-19/snsf/196396) |

*Give information separately for cases and controls in case-control studies and, if applicable, for exposed and unexposed groups in cohort and cross-sectional studies.

**Note:** An Explanation and Elaboration article discusses each checklist item and gives methodological background and published examples of transparent reporting. The STROBE checklist is best used in conjunction with this article (freely available on the Web sites of PLoS Medicine at http://www.plosmedicine.org/, Annals of Internal Medicine at http://www.annals.org/, and Epidemiology at http://www.epidem.com/). Information on the STROBE Initiative is available at www.strobe-statement.org.



For peer review only - http://bmjopen.bmj.com/site/about/guidelines.xhtml
